# Bridge editing of spin-flip emitters gives insight into excited state energies and dynamics†

**DOI:** 10.1039/d4sc05860g

**Published:** 2024-11-11

**Authors:** Florian Reichenauer, Robert Naumann, Christoph Förster, Winald R. Kitzmann, Antti-Pekka M. Reponen, Sascha Feldmann, Katja Heinze

**Affiliations:** a Department of Chemistry, Johannes Gutenberg University Mainz Duesbergweg 10-14 55128 Mainz Germany Katja.Heinze@uni-mainz.de; b Rowland Institute, Harvard University 100 Edwin H. Land Boulevard Cambridge MA 02142 USA; c Institute of Chemical Sciences and Engineering, École Polytechnique Fédérale de Lausanne Lausanne Switzerland

## Abstract

Six-coordinate chromium(iii) complexes with high spin-flip (SF) photoluminescence quantum yields and lifetimes (molecular rubies) have attracted huge interest in the past years due to their applicability in sensing, photocatalysis or circularly polarised emission. However, clearcut design rules for high quantum yields and lifetimes are still lacking due to the multidimensional problem of the non-radiative decay of the SF states. Based on an isostructural series of complexes differing in the ligand backbone, we disentangle decisive structural and electronic features for SF excited state energies and non-radiative decays promoted by spin–orbit coupling, Jahn–Teller distortions and (thermally activated) multiphonon relaxation. This analysis goes beyond the classical increasing of the ligand field strength or the metal–ligand covalency to reduce non-radiative decay or to tune the SF energy. The results underscore the utility of the combination of near-infrared absorption, variable temperature emission and fs-transient absorption spectroscopy as well as photolysis and high-level quantum chemical calculations to obtain a comprehensive picture of the excited dynamics on ultrafast and long timescales.

## Abstract 2

The bridge X of the chelate ligand modifies doublet and quartet state energies and the excited state dynamics of luminescent chromium(iii) complexes.

## Graphical abstract

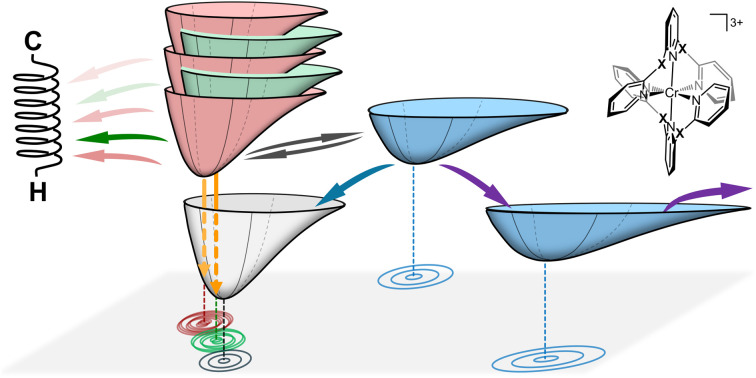

## 1 Introduction

Understanding and ultimately rationally modulating energies of metal-centered (MC) and charge-transfer (CT) excited states as well as their dynamics after light absorption are at the heart of contemporary design of transition metal complex photosensitisers and photocatalysts based on abundant elements.^1–14^ Yet, design rules for MC states typically rely on qualitative arguments based on the ligand field splitting Δ_o_, *i.e.* the energy gap between t_2g_ and e_g_ orbitals at the ground state geometry. This splitting is defined by the metal ion, the ligands and the coordination geometry according to well-known rules.^15–17^ For CT excited states, ligand modification can shift the CT states to higher or lower energies based on clear-cut substituent effects, although this chemical modification might also indirectly affect the d orbital energies.^2–4^ The dynamics of excited states^18,19^ are in general governed by their relative energies, their different geometries and electronic couplings, yet in most cases a comprehensive picture for transition metal complexes is unavailable or still subject of debate.^20–23^

Tuning of excited state energies and dynamics of metal complexes with nested spin-flip (SF)^1,4,8,9^ states as lowest energy excited states is even less understood. Yet, several high-performing 3d^3^-chromium(iii) based complexes possessing high phosphorescence quantum yields and long excited state lifetimes have been developed in recent years.^24–30^ Their useful excited state reactivities have been exploited in various applications, from sensing and photoredox catalysis to circularly polarised emission.^31–42^

The energies of the intraconfigurational excited states ^2^E/^2^T_1_ with t^3^_2g_ electron configuration in (pseudo-octahedral) transition metal complexes are essentially independent of Δ_o_.^8,43,44^ Their energies rather depend on the electron–electron repulsion as described by the Racah parameters *B* and *C* (with *C* being often approximated as multiple of *B*) in ligand field theory.^8,15,16^ Qualitatively, these parameters can be related to the nephelauxetic (cloud-expanding) effect which describes the covalent *vs.* ionic character of the bonds between the metal and the ligands.^5,8^ The expansion of the orbitals onto the ligands reduces the repulsion between individual d electrons and hence gives in principle a synthetic handle to modify SF state energies, although straightforward design rules are lacking.^45–52^

**Scheme 1:**
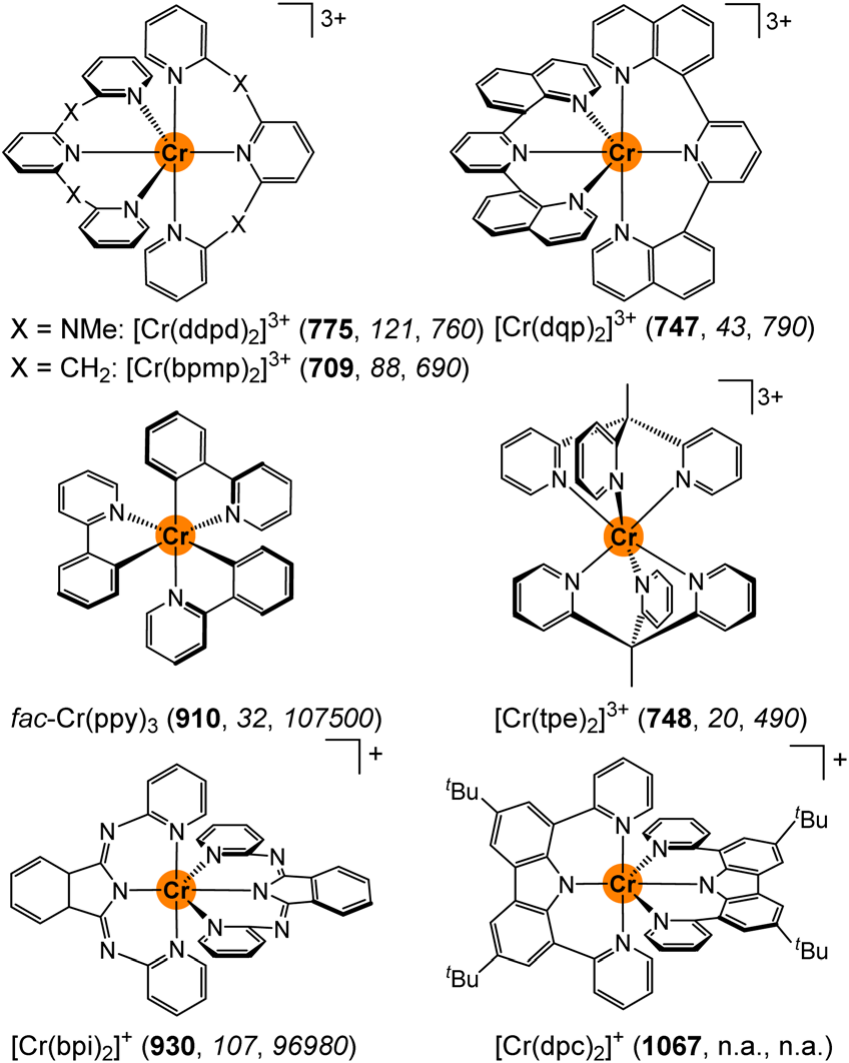
Molecular structures of [Cr(ddpd)_2_]^3+^ (ddpd = *N*,*N*′-dimethyl-*N*,*N*′-dipyridine-2-yl-2,6-diamine),^24,25^ [Cr(bpmp)_2_]^3+^ (bpmp = 2,6-bis(2-pyridylmethyl)pyridine),^29^ [Cr(dqp)_2_]^3+^ (dqp = 2,6-di(quinoline-8-yl)pyridine),^28^*fac*-Cr(ppy)_3_ (Hppy = 2-phenylpyridine),^47^ [Cr(tpe)_2_]^3+^ (tpe = 1,1,1-tris(pyrid-2-yl)ethane),^27^ [Cr(bpi)_2_]^+^ (Hbpi = 1,3-bis(2′-pyridylimino)-isoindoline),^50^ and [Cr(dpc)_2_]^+^ (Hdpc = 3,6-di-*tert*-butyl-1,8-di(pyridine-2-yl)-carbazole),^46^ along with their emission wavelengths (*λ*_em_/nm) in bold and the radiative and non-radiative rate constants (*k*_r_/s^−1^, *k*_nr_/s^−1^) in italics in solution at room temperature (n.a. = not available).

With pyridine and amine based ligands, chromium(iii) complexes typically emit between *ca.* 680–780 nm (14 700–12820 cm^−1^).^1^ π-Electron donating amido (carbazolato or isoindolinato) donors^46,50^ and cyclometalating ligands^45,47^ shift the ruby-like luminescence above 900 nm (Scheme 1).^1^ Apart from changing the donors from amines/pyridines to anionic donors, even the bridging unit between coordinating pyridines affects the SF energies. In particular, the highly emissive polypyridyl chromium(iii) complexes [Cr(ddpd)_2_]^3+^ ([1^NMe^]^3+^) and [Cr(bpmp)_2_]^3+^ ([1^CH2^]^3+^) (molecular rubies) with NMe and CH_2_ bridges emit at 775/738 and 709 nm, respectively, although both complexes share a very similar six-fold pyridine coordination environment (Scheme 1).^24,29^ While the correct energy level ordering of the two complexes could be predicted by high-level quantum chemical calculations, the underlying origin of the 1200 cm^−1^ emission energy difference remained unclear.^29^ Under hydrostatic pressure, the emission energy of [1^NMe^]^3+^ shifts by 13.9 ± 0.9 cm^−1^ kbar^−1^ to lower energy slightly depending on the environment.^33^ Compared to the pressure sensitivity of the ^2^E emission of Al_2_O_3_ : Cr with 0.7 cm^−1^ kbar^−1^, this sensitivity is much more pronounced.^53^ A compression of the Cr–N bonds and a co-planarisation of the central pyridine rings (Py_c_) of the tridentate ligands leads to better overlap with ligand orbitals and thus to “cloud expansion”.^54^ The lowest doublet state is of ^2^T_1_ parentage with two paired electrons in a t_2g_-derived orbital showing the larger shift under pressure as compared to the classical lowest doublet states of ^2^E parentage with singly occupied t_2g_ orbitals.^1,8,9^

Beyond the energies of the emissive SF states, the excited state dynamics are of particular importance for the overall performance of a SF luminescent complex. This includes the efficiency of the population of the doublet states *via* intersystem crossing (ISC),^55–57^ as well as the radiative and non-radiative decay to the ground state (*k*_r_ and *k*_nr_). The radiative rate constant *k*_r_ of the Laporte-forbidden^58^ emission depends on the symmetry of the complex, with centrosymmetric complexes such as [Cr(tpe)_2_]^3+^ (Scheme 1) or [Cr(CN)_6_]^3–^ exhibiting very small radiative rate constants down to 20 and 0.4 s^−1^, respectively.^1,27^

Several processes contribute to the non-radiative rate *k*_nr_. One reason for the small *k*_nr_ is the comparably large Δ_o_ at the Franck-Condon geometry which likely mitigates back-ISC from the SF states to the non-emissive ^4^T_2_ states with t^2^_2g_e^1^_g_ electron configuration. However, the relaxed ^4^T_2_ states are strongly Jahn–Teller distorted and experience a huge stabilisation upon distortion.^59–61^ Hence, for the excited state decay *via* back-ISC, distortional coordinates play major roles in addition to Δ_o_ at the ground state geometry. These back-ISC dynamics could be severely affected by subtle changes in the complex geometry and ligand properties, yet this has not been fully appreciated in the literature of molecular chromium(iii) emitters. For ISC in general and the ^2^E/^2^T_1_ → ^4^T_2_ back-ISC processes in particular, spin–orbit coupling (SOC) and/or spin-vibronic coupling is required.^55,56^

A further non-radiative decay path of low-energy emitters is provided through overtones of nearby CH groups as energy acceptors.^62,63^ This multiphonon relaxation depends on the distance of the CH oscillators to the metal confined excited state wave function and the spectral overlap integral of the luminescence bands with absorption bands of CH overtones.^62,63^ For most chromium(iii)-based SF emitters with pyridine ligands, the 4th aromatic CH overtone of a pyridine *ν̃*_CH_^4^ = 14 065 cm^−1^ is in the region of the emission energy providing a non-radiative decay path.^25^

In this study, we shed light on the specific factors that (i) determine the SF state energies and (ii) the population and decay (*k*_nr_) of the SF states of molecular rubies. To this end, we expand the complex series [1^NMe^]^3+^ and [1^CH2^]^3+^ by two isolobal complexes with chalcogen bridges [1^O^]^3+^ and [1^S^]^3+^. Elucidation of the ground state geometries by X-ray diffraction (XRD) analyses and excited state properties by UV/Vis/NIR absorption and emission spectroscopy is combined with high level quantum chemical modelling ((time-dependent) density functional theory, (TD)DFT and multi-reference, CASSCF-SC-NEVPT2). The excited state dynamics are probed by fs-transient absorption (TA) spectroscopy, variable-temperature (VT) emission spectroscopy, photolysis experiments and quantum chemical modelling of excited states. The combined information draw a consistent picture of excited state energies and dynamics of molecular rubies and informs about future design strategies to tune the thermodynamics and kinetics after light excitation.

## 2 Results and discussion

### 2.1 Syntheses, structures and ground state properties

The known tridentate pyridyl thioether ligand bptp (2,6-bis(pyridine-2-ylthio)pyridine)^64,65^ was prepared from 2-mercaptopyridine in 79% yield. Crystals suitable for single crystal XRD were obtained by crystallisation at low temperature. Expectedly, the C–S–C angle is with 102.7° much smaller than the angles observed at CH_2_, NMe and O bridged pyridines with 111.7°, 122.9° and 120.3°, respectively^66–68^ (ESI, Fig. S1†). The analogous synthetic route for the pyridyl ether ligand 2,6-bis(pyridine-2-yloxy)pyridine (bpop) starting from 2-hydroxypyridine (2-pyridone), however, yields mainly 2,6-bis(2-oxopyridin-1(2*H*)-yl)pyridine^69^ due to the lower nucleophilicity of oxygen compared to sulphur and the higher resonance contribution of the keto form of the deprotonated 2-pyridone compared to its enolate form.^70,71^ After extensive optimisation using various bases and solvents at different temperatures, the oxygen bridged ligand bpop could be obtained in low yields *via* substitution of 2,6-dihydroxypyridine with 2-bromopyridine besides 2,6-bis(2-oxopyridin-1(2*H*)-yl)pyridine as the major product. Both tripyridine pincer ligands bpop and bptp are fully characterised by elemental analyses, ESI^+^ mass spectrometry (ESI, Fig. S2–S4†), as well as ^1^H/^13^C NMR (ESI, Fig. S5–S15†), IR (ESI, Fig. S16–S18†) and optical spectroscopy (ESI, Fig. S19–S21†).

The orange complex salts [Cr(bpop)_2_][OTf]_3_[1^O^][OTf]_3_ and [Cr(bptp)_2_][OTf]_3_[1^S^][OTf]_3_ were prepared in 24% and 31% isolated yields, respectively, by heating an acetonitrile solution of anhydrous chromium(iii) triflate (see ESI† for a convenient route and characterisation, Fig. S22–S24†)^72^ and the corresponding ligand (Scheme 1). The purity and composition of the complexes are confirmed by elemental analyses, ESI^+^ mass spectrometry (ESI, Fig. S25 and S26†), IR spectroscopy (ESI, Fig. S27 and S28†), optical spectroscopy (ESI, Fig. S29–S32†) and electrochemistry (ESI, Fig. S33–S36†).

Electrochemical experiments reveal irreversible reduction waves at *E*_p_ = −0.48, −1.91 V and −0.40, −1.52 V *vs.* ferrocene for [1^O^]^3+^ and [1^S^]^3+^, respectively (ESI, Fig. S33–S36†), while [1^NMe^]^3+^ and [1^CH2^]^3+^ are reversibly reduced at −1.11 V and −0.81 V *vs.* ferrocene.^24,29^ DFT calculations on the dications [1^O^]^2+^ and [1^S^]^2+^ and monocations [1^O^]^+^ and [1^S^]^+^ suggest a metal centered first reduction event forming chromium(ii) species followed by a second reduction with significant ligand localisation. As chromium(ii) complexes are typically very labile,^73,74^ ligand loss or partial ligand dissociation leading to solvent coordination similar to analogous ruthenium(ii) complexes might account for the irreversibility of the reduction processes.^65^

**Fig. 1:**
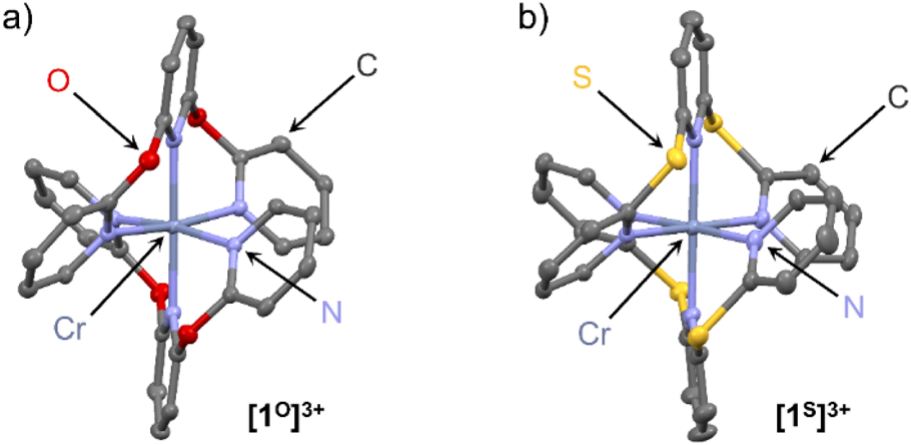
Molecular structures of the trications of the chromium complexes (a) [1^O^][OTf]_3_ and (b) [1^S^][OTf]_3_ determined by XRD. Hydrogen atoms, counter ions and co-crystallised solvent molecules omitted. Thermal ellipsoids at 50% probability level.

Single crystals of the complex salts [1^O^][OTf]_3_ and [1^S^][OTf]_3_ suitable for XRD were obtained from acetonitrile solutions at 5 °C confirming the constitution and meridional configuration (Fig. 1, ESI, Fig. S37, S38 and Tables S1, S2†). The [CrN_6_] core of the complexes is highly octahedral with N–Cr–N bond angles close to 90°/180° and almost uniform Cr–N bond lengths analogous to [1^NMe^][BF_4_]_3_ (CCDC 1059802†) and [1^CH2^][OTf]_3_ (CCDC 1989537†).^24,29^ Distinct differences are apparent in the four complex cations [1^X^]^3+^, which arise from the different bridging units X. The sp^3^-atom bridging units X = NMe, O, CH_2_, S with their averaged C–X–C angles decreasing from 122°, 121°, 115° to 104° are responsible for the chelate ring conformations and the pyridine orientations.

**Scheme 2:**
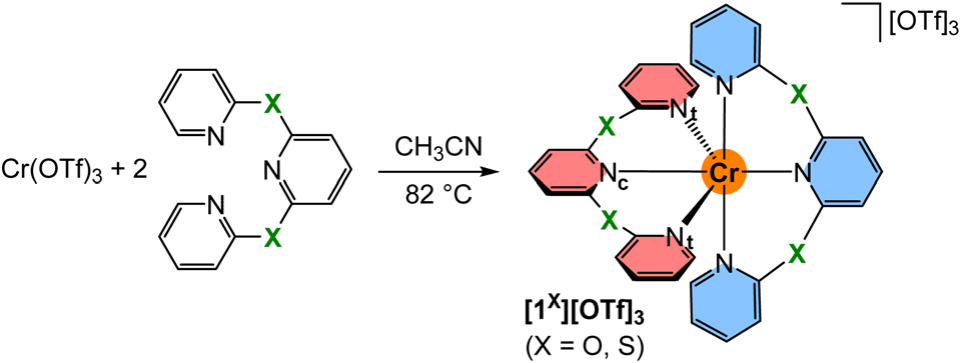
Synthesis of chromium(iii) complexes with tripyridine ligands with chalcogen atoms X = O, S in the six-membered chelate rings. Coordinating terminal and central pyridine nitrogen atoms are denoted N_t_ and N_c_, respectively. The two tridentate ligands (1) and (2) are highlighted in red and blue, respectively.

For a detailed structure description, we denote the atoms of two tridentate ligands L^X^ of [1^X^]^3+^ with (1) and (2) shown in red and blue, respectively (Scheme 2). The atoms of the central and terminal pyridines L^X^ are denoted with subscripts c and t, respectively (Scheme 2). The averaged Cr–N_t_ distances *d*_CrNt_ (Table S1†) increase from 2.042(4), 2.047(6) to 2.075(3)/2.076(3) Å for X = NMe, O, S, CH_2_. The averaged Cr–N_c_ distances *d*_CrNc_ increase from 2.038(3), 2.038(4), 2.069(2) to 2.094(2) Å for X = NMe, O, CH_2_, S. The overall size of the [CrN_6_] coordination polyhedron thus increases in the series [1^X^]^3+^ with X = NMe, O, CH_2_ and S. The N_t_ atoms of the tridentate ligands in [1^O^]^3+^ and [1^NMe^]^3+^ form N_t_(1)–Cr–N_t_(1) angles *α* (Table S1†) below 180° (*α* = 172.6(2) and 171.9(1)°), while *α* of [1^S^]^3+^ and [1^CH2^]^3+^ is closer to 180° (*α* = 177.8(1) and 177.6(1)°). The smallest N_t_(1)–Cr–N_t_(2) angle *β* between the N_t_ atoms of the two different ligands (Table S1†) is close to 90° for [1^O^]^3+^ and [1^NMe^]^3+^ (*β* = 89.7(2) and 89.8(1)°), but below 90° for [1^S^]^3+^ and [1^CH2^]^3+^ (*β* = 85.3(1) and 84.9(1)°). The angles between the planes of the terminal pyridines within a ligand are denoted by the torsion angles *δ*^1^/*δ*^2^ C^alpha^–N_t_(1)–N_t_(1)–C^alpha^ and C^alpha^–N_t_(2)–N_t_(2)–C^alpha^, respectively (Table S2†). These angles *δ*^1^ and *δ*^2^ increase from 73.5/69.8°, 72.3/71.2°, 79.1/80.6° to 81.7/86.3° for X = O, CH_2_, NMe and S approaching a more orthogonal orientation. The angles between the planes of the central pyridines of the two ligands *ϕ*, described by the torsion angle C^alpha^–N_c_(1)–N_c_(2)–C^alpha^ (Table S2†), decrease from 29.5°, 21.8°, 18.5° to 18.3° for X = CH_2_, O, S, NMe. The structural parameters *d*_CrN_, *α* and *β* of the coordination polyhedron and the orientation of the pyridines *δ*^1,2^ and *ϕ* serve to identify the most relevant structural aspects for the SF state energies in the quantum chemical modeling. The optical properties and the excited state dynamics of the complexes will be discussed next.

### 2.2 Excited state energies

To understand the optical properties of the complexes, we first describe the properties of the chalcogen bridged pyridine ligands bpop and bptp (ESI, Fig. S19–S21†). The S_0_ → S_1_ absorption bands at *λ*_max_ = 267/308 nm and the corresponding broad S_1_ → S_0_ fluorescence bands at *λ*_emis_ = 307/349 nm show large Stokes shifts of 4880 and 3810 cm^−1^, respectively. This agrees with the X → pyridine CT character of the S_1_ state assigned by TDDFT calculations (ESI, Fig. S19 and S20†) and the higher-energy sulphur lone pairs as compared to the oxygen lone pairs.

**Fig. 2:**
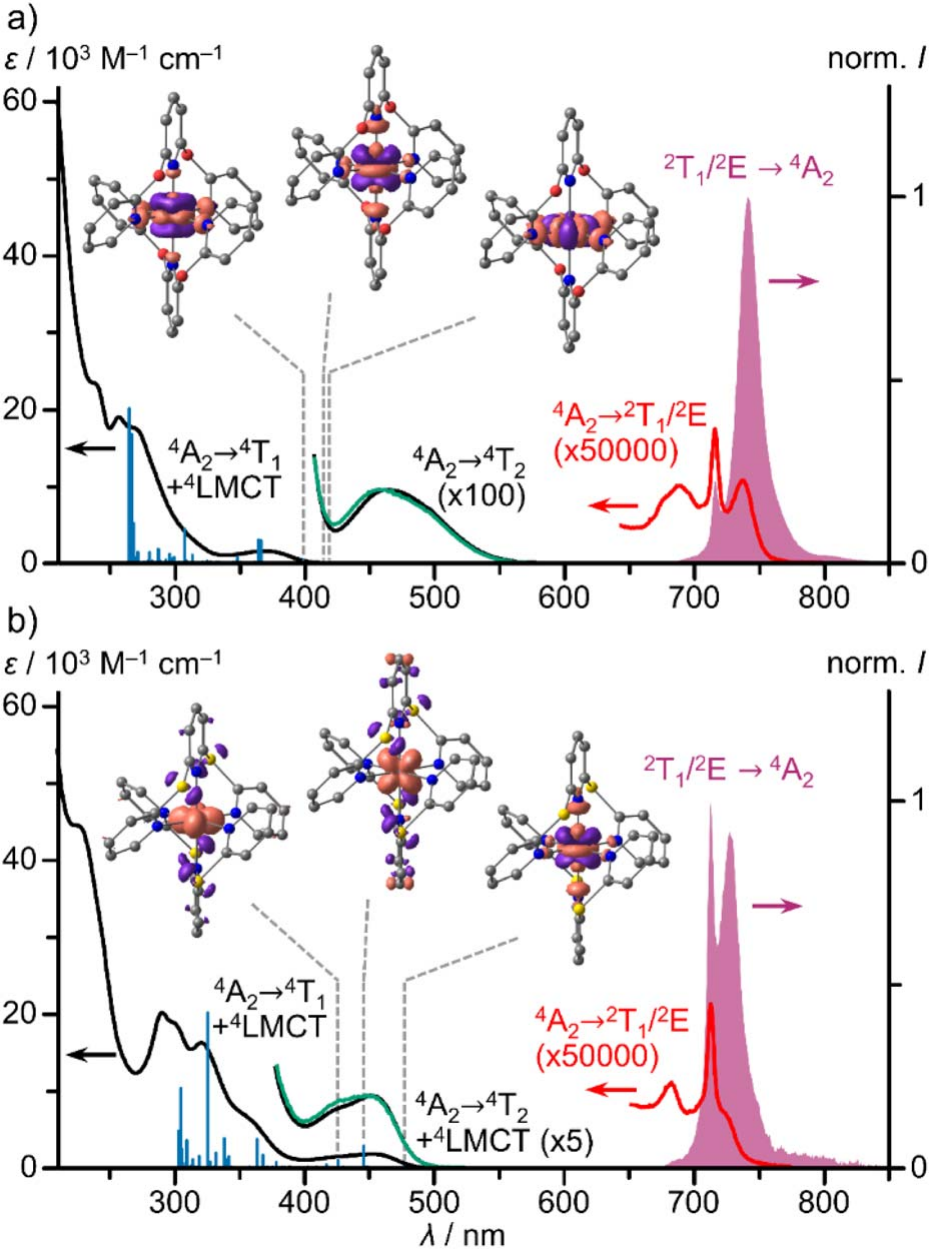
UV/Vis/NIR absorption spectra (black, red), excitation spectra (*λ*_em_ = 714 and 742 nm, green) and emission spectra (*λ*_exc_ = 450 nm, purple) of (a) [1^0^]^3+^ and (b) [1^S^]^3+^ in deaerated acetonitrile at room temperature, TDDFT calculated oscillator strengths (blue sticks) and difference electron densities of three low-energy transitions of ^4^LMCT and ^4^MC character. The regions of the spin-forbidden absorption bands, the lowest energy spin allowed/LMCT absorption band and the excitation spectrum are scaled with the indicated factors.

In the absorption spectra of the chromium(iii) complexes [1^O^]^3+^ and [1^S^]^3+^, high intensity bands of ^4^LMCT/^4^(π–π*) character, weak spin-allowed, Laporte-forbidden ^4^A_2_ → ^4^T_2_ bands and very weak spin- and Laporte-forbidden ^4^A_2_ → ^2^T_1_/^2^E bands are present in the UV, visible and NIR spectral regions, respectively (Fig. 2; ESI, Fig. S29 and S30†). The ^4^A_2_ → ^4^T_2_ bands of [1^O^]^3+^ appear isolated according to TDDFT calculations (ESI, Table S3†) at 463 nm with *ε* = 100 M^−1^ cm^−1^ (Fig. 2a), similar to ^4^A_2_ → ^4^T_2_ bands of [1^CH2^]^3+^ (465 nm; *ε* = 70 M^−1^ cm^−1^; ESI, Fig. S39†).^29^ In contrast, the analogous dd band of [1^S^]^3+^ is hidden by low energy S → Cr LMCT transitions according to TDDFT calculations (ESI, Table S4†), which increases the intensity of this band at 452 nm (Fig. 2b; *ε* = 1880 M^−1^ cm^−1^). This is analogous to the NMe → Cr LMCT transition in [1^NMe^]^3+^ at around 435 nm (*ε* = 4100 M^−1^ cm^−1^) overlapping the ^4^A_2_ → ^4^T_2_ dd transitions (ESI, Fig. S40†).^9,24^

Due to the superimposed LMCT bands and the symmetry deviating from octahedral, the ligand field splitting Δ_o_ can only roughly be estimated from the dd bands as 21 510, 21 600, 22 120 and 22 990 cm^−1^ for [1^X^]^3+^ with X = CH_2_, O, S and NMe, respectively. For a more reliable ordering of the complexes in a spectrochemical series we resorted to quantum chemical calculations. TDDFT calculated lowest energy ^4^A_2_ → ^4^T_2_ transitions at the optimised geometries, which can be assigned to the ligand field splitting Δ_o_, increase from 20 960, 23 120, 23 790 to 23 870 cm^−1^ for [1^X^]^3+^ with X = S, CH_2_, NMe and O, respectively. CASSCF(7,12)-SC-NEVPT2 calculations at the same geometries deliver 20 290, 21 740, 22 660 and 23 220 cm^−1^ for [1^X^]^3+^ with X = S, CH_2_, O and NMe, respectively (ESI, Table S5, Fig. S41†). Hence, we suggest a weaker ligand field in the [1^S^]^3+^ derivative, a medium field in [1^CH2^]^3+^ and stronger fields in the [1^O^]^3+^/[1^NMe^]^3+^ complexes. Yet, all ligand field strengths can be classified as very strong.

**Fig. 3:**
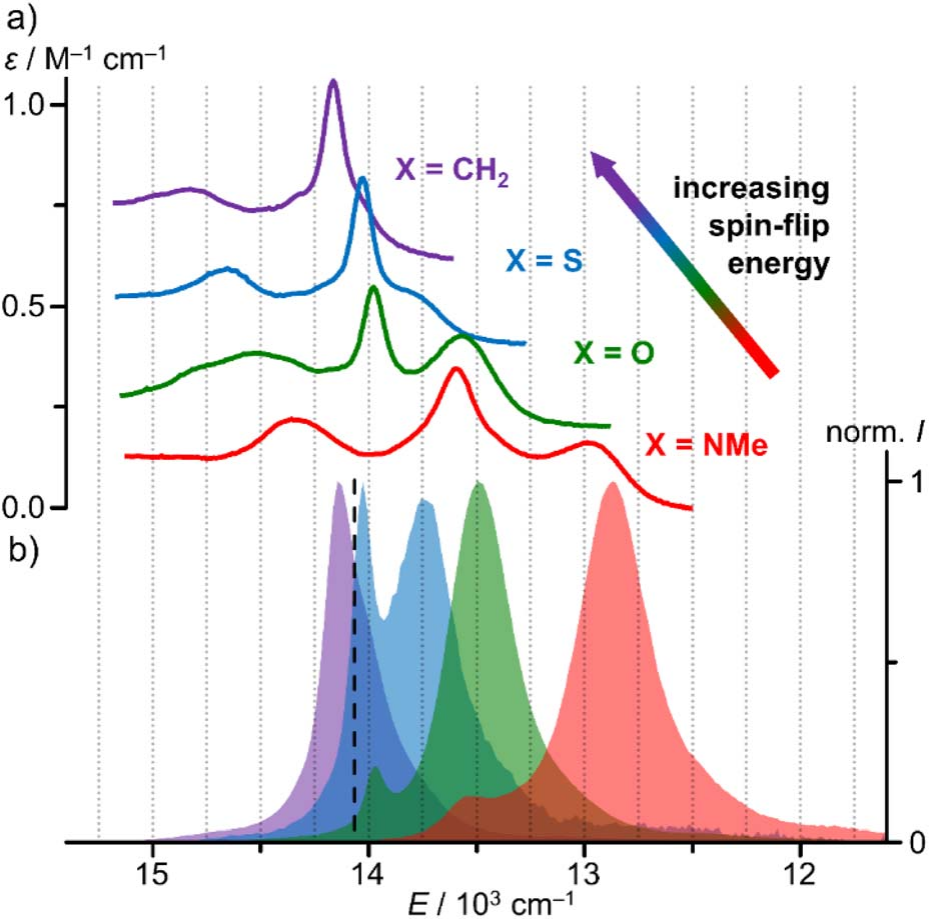
(a) NIR absorption spectra and (b) normalised emission spectra of [1^CH2^]^3+^ (purple, *λ*_exc_ = 460 nm), [1^S^]^3+^ (blue, *λ*_exc_ = 450 nm), [1^O^]^3+^ (green, *λ*_exc_ = 450 nm) and [1^NMe^]^3+^ (red, *λ*_exc_ = 435 nm) in CH_3_CN at room temperature. For clarity, the absorption spectra of [1^X^]^3+^ with X = O, S, CH_2_ were shifted vertically by +0.2, +0.4 and +0.6 M^−1^ cm^−1^. The dashed black line marks the energy of the 4th pyridine CH overtone *ν̃*_CH_4__.^25^

The NIR spectral region shows a characteristic absorption band pattern consisting of three weak bands with discernible maxima at 689, 716, 737 nm and at 683, 713, 724 (sh) nm for [1^O^]^3+^ and [1^S^]^3+^, respectively. Similar absorption band patterns of 697, 736, 771 nm and 674, 699, 706 nm were found for [1^NMe^]^3+^ and [1^CH2^]^3+^, respectively (Fig. 3a, ESI, Fig. S39 and S40†).^24,29^

As the NIR absorption bands correspond to five SF transitions (^2^E, ^2^T_1_ in octahedral symmetry), we fitted the spectral patterns with five Voigt functions each (after baseline correction as described in the ESI; Fig. S42–S45†). Indeed, these five Voigt functions excellently reproduce the experimental band patterns. The data is compiled in Table 1.

**Table 1:** Five band maxima/cm^−1^ from deconvoluted SF absorption bands of [1^X^]^3+^ (X = CH_2_, S, O, NMe), CASSCF(7,12)-SC-NEVPT2 calculated transitions (in italics; scaled by 0.89) and assignment according to the calculations. Selected energy differences/cm^−1^

No.	#5	#4	#3	#2	#1	Δ*E*(#5–#1)	Δ*E*(#2–#1)
**X = CH**_**2**_							
Exp.	15 060	14 780	14 320	14 160	14 050	1010	110
Calcd	*14 660*	*14 640*	*14 430*	*14 140*	*13 970*	*690*	*170*
Term	^2^T_1_(3)	^2^E(2)	^2^T_1_(2)	^2^E(1)^a^	^2^T_1_(1)		

**X = S**							
Exp.	14 770	14 630	14 210	14 030	13 800	970	230
Calcd	*14 750*	*14 720*	*14 500*	*14 200*	*14 000*	*750*	*200*
Term	^2^T_1_(3)	^2^E(2)	^2^T_1_(2)	^2^E(1)^a^	^2^T_1_(1)		

**X = O**							
Exp.	14 780	14 520	14 360	13 980	13 560	1220	420
Calcd	*14 510*	*14 420*	*14 160*	*14 000*	*13 590*	*920*	*410*
Term	^2^E(2)	^2^T_1_(3)	^2^T_1_(2)	^2^E(1)^a^	^2^T_1_(1)		

**X = NMe**							
Exp.	14 430	14 300	13 600	13 530	12 960	1470	570
Calcd	*14 400*	*14 250*	*13 880*	*14 180*	*13 400*	*1000*	*480*
Term	^2^E(2)	^2^T_1_(3)	^2^E(1)^a^	^2^T_1_(2)	^2^T_1_(1)		

a This is the band with a smaller experimental full width at half maximum (FWHM).

From the CASSCF(7,12)-SC-NEVPT2 calculations of all [1^X^]^3+^ complexes at their respective DFT-optimised ground state geometries we obtained the five lowest doublet state energies (ESI, Table S5, Fig. S41†). With a common scaling factor of 0.89 these energies excellently fit to the experimentally derived energies allowing a detailed assignment of the individual states (Table 1). Importantly, the calculations reproduce the experimental nephelauxetic series of the complexes [1^X^]^3+^ with X = CH_2_, S, O and NMe derived from the SF energies, the increased lifting of the degeneracies Δ*E*(#5–#1) in the series CH_2_/S < O < NMe and the energy difference of the two lowest energy SF absorptions Δ*E*(#2–#1) (Table 1). The very sharp central band (doublet state #3 for X = NMe, #2 for the other complexes) reflects a miniscule geometric distortion of an excited state and can most likely be assigned to a (0,0) transition of a nested, *i.e.* undistorted, microstate of ^2^E character.^1,8^ The tentative assignment of the SF state #2 as ^2^E(1) is confirmed by the (7,12)-SC-NEVPT2 calculations (Table 1). Based on these agreements, we attest a high fidelity to the quantum chemical calculations and assignments. In all cases, the lowest doublet states #1 and #2 are of ^2^T_1_ and ^2^E character, respectively.

Irradiation of a solution of [1^O^]^3+^ and [1^S^]^3+^ at room temperature in acetonitrile with *λ*_exc_ = 450 nm gives rise to two sharp emission bands peaking at 716/741 nm and 713/727 nm with FWHM of 140/350 and 150/420 cm^−1^, respectively (Fig. 3b, ESI, Fig. S31†). The energies of the two phosphorescence bands of all complexes [1^X^]^3+^ match the energies of the two lowest-energy absorption bands (Fig. 3; ESI, Fig. S39 and S40†).^9,24,29^ Small Stokes shifts of 80, 60, 70 and 70 cm^−1^ are observed for the lowest energy emission band (^2^T_1_(1) → ^4^A_2_), while even smaller Stokes shifts <40 cm^−1^ are determined for the second lowest energy emission band (^2^E(1) → ^4^A_2_) in agreement with slightly distorted ^2^T_1_(1) states and almost perfectly nested ^2^E(1) states (Fig. 3).

Doublet states of ^2^E and ^2^T_1_ parentage could be localised by excited state geometry optimization *via* TDDFT. The Cr–N distances *d*_CrN_ slightly decrease from *d*_CrNc_/*d*_CrNt_ = 2.051/2.071 Å (^4^A_2_ ground state) to 2.044/2.064 Å (^2^E) and 2.042/2.057 Å (^2^T_1_) for X = O and from *d*_CrNc_/*d*_CrNt_ = 2.140/2.118 Å (^4^A_2_ ground state) to 2.103/2.087 Å (^2^E) and 2.098/2.079 Å (^2^T_1_) for X = S. In both cases, the compression is larger in the ^2^T_1_(1) excited states than in the ^2^E(1) states. This further confirms that the broader low-energy bands with a larger Stokes shifts arise from the slightly more distorted ^2^T_1_(1) states.

In order to correlate structural effects of the hexapyridine chromium(iii) complexes with their doublet state energies, we first disentangle primary electronic from secondary structural effects (*d*_CrN_, *α*, *β*, *δ*^1,2^ and *ϕ*) of the bridging unit X. In a first series of calculations, the bridging unit was substituted by a different bridge while retaining the geometry (ESI, Fig. S46, Table S6†). For example, the NMe unit of [1^NMe^]^3+^ was replaced by X = O and CH_2_, while the original geometry of [1^NMe^]^3+^ was retained. According to the CASSCF(7,12)-SC-NEVPT2 calculations, NMe → O replacement increases the SF energy, while NMe → CH_2_ replacement decreases it (ESI, Fig. S46, Table S6†). The latter is at odds with the experiment, suggesting that electronic substituent effects (at the *ortho* positions) alone do not correctly describe the SF energy shifts. Furthermore, the energy variation at fixed geometries but with different X bridges is smaller than the energy variation due to geometric distortions (ESI, Fig. S46†). Hence, the indirect effects of structural modification (*d*_CrN_, *α*, *β*, *δ*^1,2^ and *ϕ*) exerted by the bridge X are more relevant for the SF energies than primary electronic effects.

**Scheme 3:**
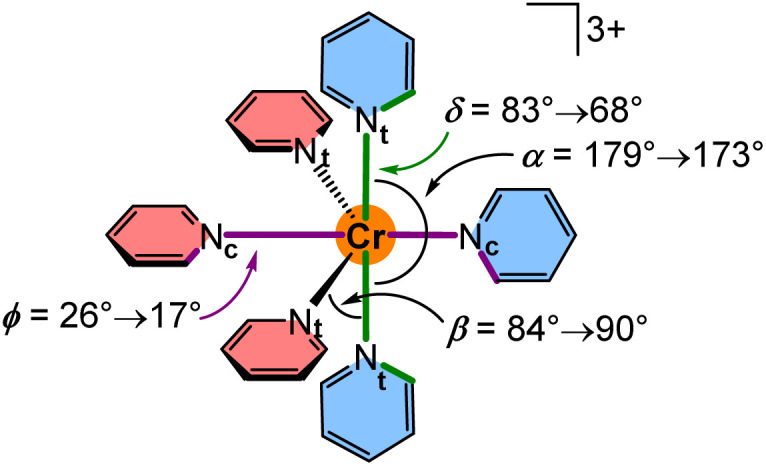
Structure of the model system [Cr(py)_6_]^3+^ and the definition and relevant ranges of the bond angles *α*/*β* and the dihedral angles *δ*/*ϕ*.

To explore the effects of the geometry on the SF energies, we employed a model system [Cr(py)_6_]^3+^ where contributions from the bridging atoms are excluded (Scheme 3). The parameters *d*_CrN_, *α*, *β*, *δ* and *ϕ* were varied within the value ranges as determined from XRD analyses and DFT optimisations and also extrapolated to smaller and larger values (ESI, Fig. S47–S57†). Within this set of parameters, decreasing the Cr–N distances *d*_CrNc_ = 2.140 → 2.046 Å and *d*_CrNt_ = 2.118 → 2.058 Å shifts the SF energies to lower energy by 225 and 228 cm^−1^, respectively, at fixed *α*, *β*, *δ* and *ϕ* angles in the calculations (Fig. S58†). Hence, symmetric compression of the [CrN_6_] coordination polyhedron in local *D*_4h_ symmetry results in bathochromic shifts of the SF energies. This bathochromic shift is also observed for ruby^53^ and molecular rubies under pressure (ESI, Fig. S48–S51†).^35,54^ Shorter Cr–N bonds enhance the covalency of the bonds, thereby increasing the electron delocalization and hence the nephelauxetic effect resulting in lower SF energies. Expectedly the energetic order of the five individual SF microstates is essentially unaffected by the symmetrical Cr–N modes in the observed distance range. Decreasing the angle *α* in the model [Cr(py)_6_]^3+^ (ESI, Fig. S52†) from 179° (as found in [1^S^]^3+^) to 173° (as found in [1^NMe^]^3+^) at fixed Cr–N distances and *ϕ* angles (*β* and *δ* had to be adjusted in order to avoid close H⋯H contacts) lowers the SF energy by 75 cm^−1^ (ESI, Fig. S58†). This unsymmetrical distortion also changes the ^2^T_1_/^2^E contributions to the individual microstates. Expansion of *β* from 84° (as found in [1^CH2^]^3+^) to 90° (as found in [1^O^]^3+^) lowers the SF energy by 28 cm^−1^ (ESI, Fig. S58†). The torsional modes *δ* (ESI, Fig. S54 and S55†) and *ϕ* (ESI, Fig. S56 and S57†) exert weaker effects. Creating a less orthogonal orientation of terminal pyridines, *i.e.* adjusting *δ* = 83 → 68° ([1^S^]^3+^ → [1^O^]^3+^) and more co-planar orientations of the central pyridines, *i.e.* adjusting *ϕ* = 26 → 17° ([1^CH2^]^3+^ → [1^S^]^3+^) leads to very small bathochromic shifts of 9 and 5 cm^−1^, respectively (ESI, Fig. S58†).

Consequently within the present complex series [1^X^]^3+^, the bridging unit X indirectly affects both the average energy of the ^2^T_1_/^2^E manifold and the relative energies of the five doublet microstates with ^2^T_1_ and ^2^E parentage. Bond compression exerts the largest bathochromic effect, followed by lowering the N_t_–Cr–N_t_ bite angle *α*, although the angular modifications are not independent from each other in the chelate complexes. Having assigned the character of the lowest energy excited states and the structural origins of the relative energies, we now turn to the excited state dynamics.

### 2.3 Excited state dynamics

Excitation spectra of [1^O^]^3+^ and [1^S^]^3+^ (*λ*_em_ = 742 and 714 nm) closely follow the respective absorption spectra in the region of the lowest spin-allowed ^4^A_2_ → ^4^T_2_ and ^4^LMCT transitions (*ca.* 380–600 nm, Fig. 3). This substantiates that both types of quartet states efficiently populate the SF states *via* ISC without branching to other decay channels.

Femtosecond TA spectroscopy on [1^O^]^3+^ and [1^S^]^3+^ confirms that the vibrationally cold doublet states are populated within *τ*_1_,*τ*_2_ = 1.0, 47 ps and *τ*_1_,*τ*_2_ = 1.2, 90 ps (ESI, Fig. S59–S64†) after excitation at 343 nm ([1^O^]^3+^) and 515 nm ([1^S^]^3+^), respectively. In both complexes [1^O^]^3+^ and [1^S^]^3+^, ISC appears to occur on an ultrafast time scale. This rapid ISC likely arises from the high density of doublet states both for ^4^LMCT ([1^O^]^3+^) and ^4^MC/^4^LMCT ([1^S^]^3+^) excitation. For example the ^2^T_2_ derived states are close in energy to the ^4^T_2_ states according to CASSCF calculations (ESI, Table S6, Fig. S41†). No loss channels are apparent on the fast time scale and hence the decisive non-radiative and radiative decay occurs from the lowest energy SF states, similar to [1^NMe^]^3+^ and [1^CH2^]^3+^.^24,29^

The photoluminescence quantum yield *Φ* = 11.5% and SF excited state lifetime *τ*_P_ = 836 μs of [1^O^]^3+^ in deaerated CH_3_CN are in very high ranges, similar to the record values of [1^NMe^]^3+^ and [1^CH2^]^3+^.^24,29^ The observed excited state lifetime corresponds to the common lifetime of the equilibrating emissive lowest energy doublet states ^2^T_1_(1) and ^2^E(1).^31^ The radiative and non-radiative rate constants *k*_r_ = 138 s^−1^ and *k*_nr_ = 1059 s^−1^ are similar to the values of [1^NMe^]^3+^ and [1^CH2^]^3+^ (Scheme 1). On the other hand, the values of the sulphur derivative [1^S^]^3+^ are lower by orders of magnitude with *Φ* = 0.01% and *τ*_P_ = 1.65 μs. This gives a somewhat smaller *k*_r_ = 61 s^−1^, while the non-radiative rate constant *k*_nr_ = 606 000 s^−1^ increased by orders of magnitude.

**Fig. 4:**
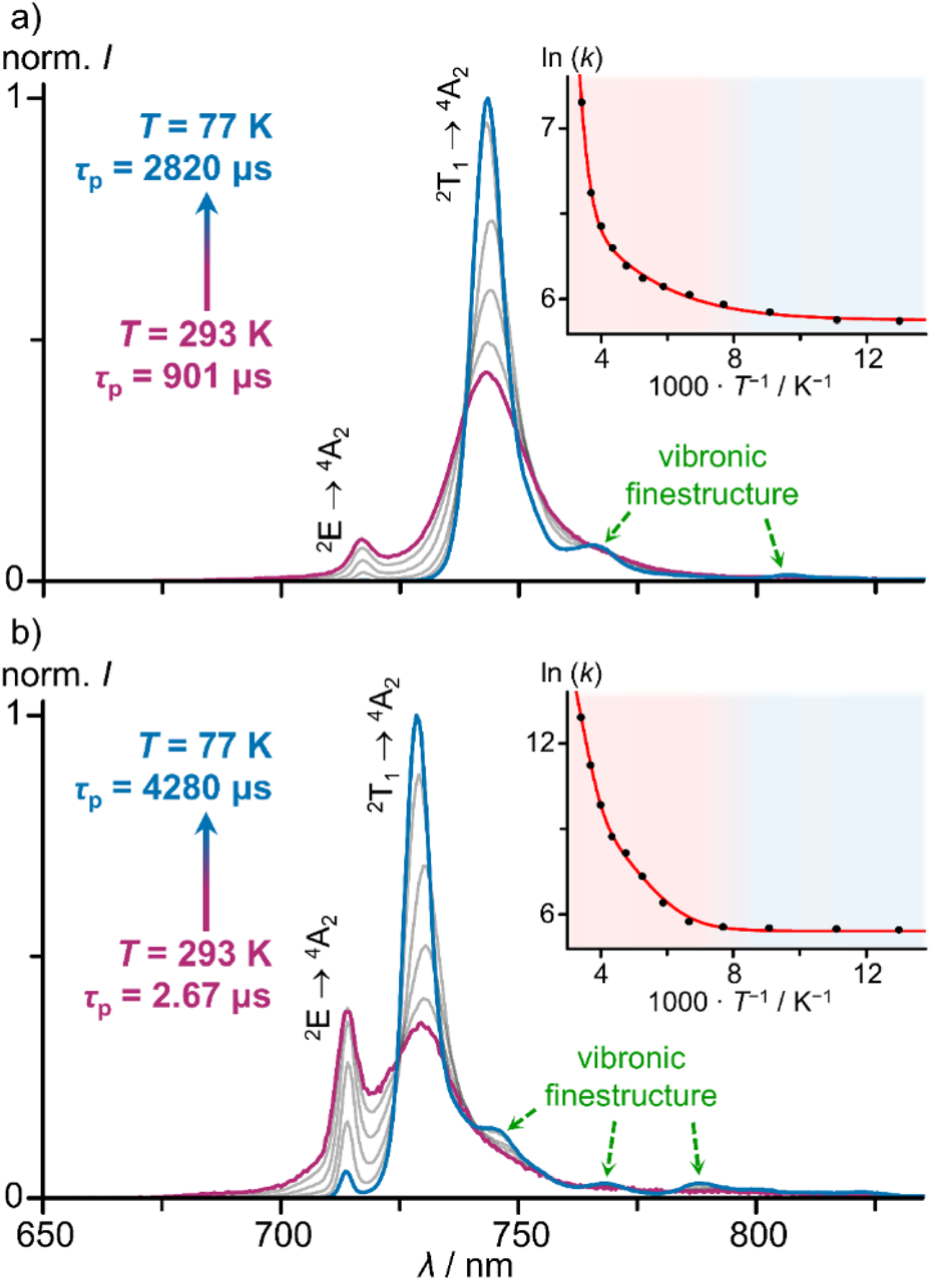
Integration area normalised low temperature emission spectra (*λ*_exc_ = 450 nm) of (a) [1^0^]^3+^ and (b) [1^S^]^3+^ in a mixture of ethanol/methanol (3 : 2 v/v) between 77 and 293 K under deaerated conditions. Insets show corresponding Arrhenius plots of the photoluminescence rate constants *k*(*T*) with fit and coloured backgrounds for temperature ranges of liquid (red) and frozen solvent mixture (blue).

As this huge difference of *k*_nr_ is completely unexpected in light of the similar ground state geometries and excited state energies, we investigated the excited decay of [1^O^]^3+^ and [1^S^]^3+^ by variable-temperature (time-resolved) emission spectroscopy in order to identify thermally accessible loss channels. Emission spectra and lifetimes of [1^O^]^3+^ and [1^S^]^3+^ were determined between 293 and 77 K in ethanol/methanol (3 : 2 v/v) solution, which freezes around 130 K (Fig. 4).

Temperature-dependent emission spectra clearly reveal thermal population of the two lowest energy SF states for all complexes under study and even population of the next higher energy SF state #3 ^2^T_1_(2) to a small degree (ESI, Fig. S65–S68†). At lower temperatures, the lowest SF state ^2^T_1_(1) is increasingly populated at the expense of the other SF states. The ^2^T_1_(1) → ^4^A_2_ emission bands develop vibrational fine structure at lower temperature with maxima centred at 13 050, 12 390, 12 210, 11 830 cm^−1^ and 13 440, 13 020, 12 690, 12 480, 12 150 cm^−1^ for [1^O^]^3+^ and [1^S^]^3+^, respectively (ESI, Fig. S65 and S66†). Similar vibrational progressions have been reported for [1^NMe^]^3+^ and [1^CH2^]^3+^ (ESI, Fig. S67 and S68†).^24,29^ The energy differences are compatible with Cr–N vibrational modes.^75^

Cooling the solution of [1^O^]^3+^ from 293 K to 77 K sharpens the low-energy ^2^T_1_(1) → ^4^A_2_ band (FWHM = 350 → 150 cm^−1^) and increases the lifetime *τ*_P_ = 901 → 2820 μs (Fig. 4a; ESI, Fig. S69†). At 250 K, the quantum yield reaches approximately *Φ* = 22%. Similarly, cooling a solution of [1^S^]^3+^ from 293 K to 77 K sharpens the low-energy ^2^T_1_(1) → ^4^A_2_ band (FWHM = 420–140 cm^−1^) and drastically increases the lifetime *τ*_P_ = 2.67 → 4280 μs (Fig. 4b, ESI, Fig. S70†). Concomitantly, the integrated luminescence intensity increases 900-fold, *i.e.* approaching approximate values of *Φ* = 9% at 77 K (assuming a temperature independent absorbance at the excitation energy).

The thermally activated decay of [1^O^]^3+^ and [1^S^]^3+^ was successfully modelled with an Arrhenius-like behaviour according to eqn (1) (Fig. 4). The photoluminescence rate constants *k*(*T*) = 1/*τ*_p_(*T*) of the complexes (ESI, Fig. S69 and S70†) were fitted as a sum of a *T*-independent rate constant *k*_0_, describing non-thermally activated radiative and non-radiative processes, and two *T*-dependent rate constants *k*_1_(*T*) and *k*_2_(*T*) (eqn (1)).1
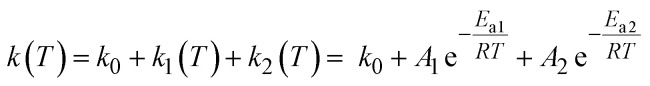


Two thermally activated processes (*A*_1_, *E*_a1_; *A*_2_, *E*_a2_) were required to satisfactorily fit the experimental data (Table 2). *k*_1_ (*A*_1_, *E*_a1_) and *k*_2_ (*A*_2_, *E*_a2_) dominate the high and low temperature regions, respectively.

**Table 2:** Fit parameters for the emission decays of solutions of [1^O^]^3+^ and [1^S^]^3+^ obtained from Arrhenius plots according to eqn (1)

	[1^O^]^3+^	[1^S^]^3+^
*k*_0_/s^−1^	356	225
*E*_a1_/cm^−1^ (eV)	3360 (0.417)	4320 (0.536)
*E*_a2_/cm^−1^ (eV)	370 (0.046)	1090 (0.135)
*A*_1_/s^−1^	9.18 × 10^9^	6.27 × 10^14^
*A*_2_/s^−1^	1.80 × 10^3^	4.65 × 10^6^

Based on the two experimentally determined barriers *E*_a1_ and *E*_a2_ for thermally activated decay pathways with high and small frequency factors *A*_1_ and *A*_2_ (Table 2), we now propose a kinetic model with physical assignments to the processes. The larger barriers *E*_a1_ of 3360 and 4320 cm^−1^ for [1^O^]^3+^ and [1^S^]^3+^ are likely associated with the thermally activated back-ISC from the ^2^T_1_(1)/^2^E(1) levels to the quartet levels, possibly along several conceivable Jahn–Teller modes associated with the ^4^T_2_ or ^4^LMCT states similar to ^3^MC states of d^6^ metal complexes such as [Ru(bpy)_3_]^2+^ (bpy = 2,2′-bipyridine).^76–79^

Excited state geometry optimisations of the complex cations by TDDFT (ESI, Table S7†) delivered quartet excited states with ^4^T_2_ character and one or two elongated Cr–N_c_ distances. The symmetric N_c_–Cr–N_c_ breathing elongation amounts to 9% and 6–8% for [1^O^]^3+^ and [1^S^]^3+^, while the more asymmetric Cr–N_c_ elongation reaches 22% and 13% for [1^O^]^3+^ and [1^S^]^3+^, respectively. Asymmetric ^4^T_2_ states were also localised for X = [1^CH2^]^3+^ and [1^NMe^]^3+^ where a single Cr–N_c_ bond is elongated by 20% and 13%, respectively. In addition to the distorted metal-centered ^4^T_2_ states of [1^S^]^3+^, two distorted ^4^LMCT states are located at similar energies. One optimised ^4^LMCT coordination polyhedron is elongated along the symmetric N_c_–Cr–N_c_ breathing mode (10–15% elongation) and the other optimised state exhibits a pincer-like deformation with a 14% increase of two Cr–N_t_ bond lengths of a single bptp ligand. In essence, all complexes [1^X^]^3+^ possess low-energy distorted metal-centered quartet states, while [1^S^]^3+^ additionally possesses distorted low-energy ^4^LMCT states with significant S → Cr CT character.

The experimental barrier *E*_a1_ of the oxygen derivative [1^O^]^3+^ is smaller than that of the sulphur derivative [1^S^]^3+^. This suggests favoured thermally activated non-radiative decay for [1^O^]^3+^, which is at odds with the experimentally observed lower *k*_nr_ of [1^O^]^3+^ at first sight. However, the frequency factor *A*_1_ determined for [1^S^]^3+^ is higher than that for [1^O^]^3+^ by orders of magnitude, which overcompensates the somewhat higher barrier of [1^S^]^3+^ (Table 2). In a picture of semi-classical Marcus theory, larger frequency factors are associated with a stronger electronic coupling of the involved states.^80^ In other words, although [1^O^]^3+^ has smaller barriers, its electronic coupling is much weaker leading to a smaller ISC transition probability. For [1^S^]^3+^, the large frequency factor *A*_1_ enables rapid excited state decay. The larger *A*_1_ might arise from a higher density of accessible distorted quartet states in [1^S^]^3+^ (Table S7†) and/or higher SOC, *i.e.* higher electronic coupling. We hypothesise that the heavier sulphur atoms invoke larger SOCs^16,81^ as compared to the lighter bridging carbon, nitrogen or oxygen atoms of [1^CH2^]^3+^, [1^NMe^]^3+^ and [1^O^]^3+^ and hence promote the ^2^T_1_(1)/^2^E(1) → ^4^T_2_/^4^LMCT back-ISC process *via* the heavy atom effect.^82–85^ The sulphur orbital contributions to the ^4^LMCT state could further assist the ^2^T_1_(1)/^2^E(1) → ^4^LMCT back-ISC process *via* SOC.^55,56^

Following this simple argument, the lower *Φ* of [1^S^]^3+^ at room temperature is associated with a heavy-atom promoted thermally activated back-ISC to ^4^T_2_/^4^LMCT levels. As these distorted ^4^T_2_/^4^LMCT states can cross the ^4^A_2_ ground state potential surface or can lead to ligand dissociation (see below), this back-ISC process can be irreversible.

The second barriers *E*_a2_ of a few hundred wavenumbers might be associated with the thermal population of the five lowest doublet states (Table 1 and Fig. 4). This reversible equilibration of doublet states, however, should not promote non-radiative decay *per se* as these doublet states are essentially nested states. Yet, multiphonon relaxation, which requires a resonant energy transfer to a vibrational overtone, might become relevant upon populating higher energy SF states. The 4th aromatic CH overtone of a pyridine of *ν̃*_CH_4__ = 14 065 cm^−1^ is in the region of the SF states for both complexes (Table 1 and Fig. 3b).^25^ Accidently, the ^2^E(1) state of [1^S^]^3+^ is even almost resonant with *ν̃*_CH_4__. Hence, a diminished ^2^E(1) population of [1^S^]^3+^ at lower temperature will mitigate this pathway significantly. Inspection of Fig. 4b shows that the ^2^E(1) → ^4^A_2_ emission band of [1^S^]^3+^ dramatically diminishes in intensity at 77 K relative to the ^2^T_1_(1) → ^4^A_2_ emission band. This effect is much less pronounced for [1^0^]^3+^ due to its larger *E*(^2^E(1)–^2^T_1_(1)) energy difference (Table 1). In addition, its ^2^E(1) emission band has a much smaller spectral overlap integral with the CH overtone anyway thanks to the larger energy gap *E*(^2^E(1)–*ν̃*_CH_4__). Consequently, we propose that multiphonon relaxation *via* CH overtones is more relevant for [1^S^]^3+^ and that this pathway is *T*-dependent due to thermally activated population of its (accidently resonant) ^2^E(1) state.

The extremely efficient non-radiative decay of [1^S^]^3+^ with *k*_nr_(293 K) = 606 000 s^−1^*via* the ^4^T_2_/^4^LMCT and ^2^E(1) levels is strongly diminished at 77 K with *k*_nr_(77 K) = 213 s^−1^. The first decay path is associated with the presence of a sulphur atom with larger SOC for the ^2^T_1_(1)/^2^E(1) → ^4^T_2_/^4^LMCT ISC (internal heavy atom effect) and the second decay path is associated with the thermally populated ^2^E(1) level that is accidently resonant with a CH overtone (thermally activated multiphonon relaxation).

### 2.4 Excited state reactivity

The photostability of the complexes [1^X^]^3+^ was probed by irradiating them in deaerated CH_3_CN (X = CH_2_, S, O, NMe). High light intensities are required for significant photochemical responses which is why we used a 460 nm UHP-LED with an output power of 1.1 W for these experiments. Under these conditions, which also might include a rise in temperature, the absorption spectra change and the luminescence intensities decrease in all cases (Fig. S71–S74†). The resulting spectra are compatible with (partial) ligand dissociation with the dissociated tripyridine ligands possibly undergoing follow-up reactions such as protonation (from the environment) or oxidation by excited chromium complexes.^67^

The ligand photodissociation is not very efficient for [1^O^]^3+^, [1^NMe^]^3+^ and [1^CH2^]^3+^, but appears more efficient for [1^S^]^3+^, although the rates are not entirely comparable due to the different absorption changes during the photolysis. As discussed above, the heavy-atom effect of sulphur^82–85^ and the higher density of states might again be made responsible for the more efficient ^2^E/^2^T_1_ → ^4^T_2_(1)/^4^LMCT back-ISC of [1^S^]^3+^ and hence the more efficient unimolecular ligand dissociation. A potentially dissociative state could be the ^4^LMCT state with a pincer-like deformation of one bptp ligand (ESI, Table S7†).

For all complexes, the excited state lifetimes are sufficiently high (>1.5 μs) at room temperature to allow for bimolecular quenching. The doublet states of all sensitisers [1^X^]^3+^ are quenched by Dexter energy transfer to triplet oxygen with rate constants *k*_q_ = 0.17 × 10^7^ M^−1^ s^−1^, 0.29 × 10^7^ M^−1^ s^−1^,^29^ 1.77 × 10^7^ M^−1^ s^−1^,^36^ 8.55 × 10^7^ M^−1^ s^−1^ for X = O, CH_2_, NMe, S, respectively (Fig. S75 and S76†). This series might be associated with the different accessibility of the Cr centre by O_2_ due to the different bridges X. Singlet oxygen quantum yields were already reported for [1^NMe^]^3+^ and [1^CH2^]^3+^ as 61% (DMF) and 55% (DMF/HClO_4_), respectively.^29,32^

Based on the high excited state reduction potentials of [1^O^]^3+^ and [1^S^]^3+^ of *E*(*[1^X^]^3+^/[1^X^]^2+^) = 1.25 and 1.34 V (derived from the ^2^E(1) energies at 293 K), these sensitisers should react with suitable redox-active quenchers. Anthracene (*E*_1/2_ = 0.69 V *vs.* ferrocene, *E*_T_ = 1.85 eV)^86,87^ and *trans*-stilbene (*E*_1/2_ = 1.03 V *vs.* ferrocene, *E*_T_ = 2.14 eV)^86,87^ should quench excited [1^O^]^3+^ and [1^S^]^3+^ exclusively by electron transfer, but not by energy transfer due to their high triplet energies *E*_T_. The larger driving force for the oxidation of anthracene compared to *trans*-stilbene gives quenching rate constants *k*_q_(anthracene) = 7.21 × 10^8^/44 × 10^8^ M^−1^ s^−1^ larger by two orders of magnitude than for *trans*-stilbene with *k*_q_(*trans*-stilbene) = 2.06 × 10^6^/27.3 × 10^6^ M^−1^ s^−1^ for [1^O^]^3+^ and [1^S^]^3+^, respectively (Fig. S77–S80†). Both quenchers react faster with the sulphur derivative [1^S^]^3+^ than with [1^O^]^3+^ due to the higher excited state potential of [1^S^]^3+^ in agreement with Marcus theory.

## 4 Conclusions

Two new molecular rubies [1^X^]^3+^ with X = O, S bridging atoms in the tripyridine ligands were structurally and spectroscopically characterised complementing the analogous known spin-flip emitters with X = NMe, CH_2_. This series allows an unprecedented insight into excited state energies and dynamics. The energies of all five lowest doublet levels derived from ^2^E/^2^T_1_ terms in octahedral symmetry of all complexes were experimentally identified by absorption spectroscopy for the first time. High-level quantum chemical calculations reproduced these doublet state energies very well. Calculations on model systems revealed the underlying structure–property relationship with the dominant parameter for the emission energy being the Cr–N distances: shorter distances lead to bathochromic shifts, which can be explained on the basis of the nephelauxetic effect.

All four molecular rubies [1^X^]^3+^ undergo ultrafast ISC to the doublet manifold. The excited state dynamics of [1^X^]^3+^ with X = O, CH_2_, NMe on longer times scales are similar and high quantum yields and lifetimes are obtained. The sulphur derivative [1^S^]^3+^, however, experiences extremely rapid non-radiative decay *via* two pathways at room temperature: The first decay path is likely associated with stronger spin–orbit coupling caused by the sulphur atoms leading to faster doublet-quartet back-intersystem crossing (internal heavy atom effect). The second decay pathway is opened by thermal population of the ^2^E(1) level that is accidently resonant with a CH overtone enabling multiphonon relaxation. The efficient population of the distorted quartet states in [1^S^]^3+^ furthermore promotes more facile ligand dissociation. All sensitisers [1^X^]^3+^ engage in photoinduced energy and electron transfer reactions from their doublet levels thanks to their microsecond excited state lifetime.

Key results from this study are as follows: (i) the Cr–L distances appear as the most important parameter to tune the luminescence energies of spin-flip emitters (nephelauxetic effect); (ii) with a large ligand field splitting, the density of doublet states at the Franck-Condon geometry is high, which enables efficient ISC without requiring SOC by further heavy atoms; (iii) heavy atoms should indeed be avoided as they can promote thermally activated back-ISC to the quartet levels along Jahn–Teller modes, increasing *k*_nr_ (heavy atom effect); (iv) Jahn–Teller distortions in ^4^T_2_/^4^LMCT states are responsible for photodissociation and (v) CH overtones of the ligands (in particular CH oscillators those close to the metal centre) can define spectral regions of the luminescence with higher and lower non-radiative decay *via* (thermally activated) multiphonon relaxation. These key aspects will aid in future design concepts of improved molecular rubies.

## Data availability

The data supporting this article have been included as part of the ESI.†

## Author contributions

F. R. performed the syntheses and spectroscopic characterization as well as the quantum chemical calculations. R. N. and F. R. measured and interpreted the emission data. C. F. solved and refined the single crystal structures and assisted with the quantum chemical calculations. W. R. K., A. M. R. and S. F. conducted TA measurements. K. H. conceived and designed the project. F. R. and K. H. wrote the manuscript with contributions of all authors. K. H. supervised and C. F. co-supervised the project.

## Conflicts of interest

There are no conflicts to declare.

## Supplementary Material

SC-OLF-D4SC05860G-s001

SC-OLF-D4SC05860G-s002

SC-OLF-D4SC05860G-s003
