# Supplementary material for: Bridge editing of spin-flip emitters gives insight into excited state energies and dynamics
Source: Chem Sci. 2024 Nov 11;15(48):20251–62. doi: 10.1039/d4sc05860g (PMC11575611; doi:10.1039/d4sc05860g)
Supplement: SC-015-D4SC05860G-s001 [file SC-015-D4SC05860G-s001.pdf]

Supplementary Information (SI) for Chemical Science.
This journal is © The Royal Society of Chemistry 2024

Supplementary Information

Bridge Editing of Spin-Flip Emitters gives Insight into Excited State Energies and Dynamics

Florian Reichenauer,[a] Robert Naumann,[a] Christoph Förster,[a] Winald R. Kitzmann,[a] Antti-Pekka M. Reponen,[b] Sascha Feldmann[b] and Katja Heinze*[a]

[a] Department of Chemistry, Johannes Gutenberg University Mainz, Duesbergweg 10-14, 55128 Mainz, Germany

[b] Rowland Institute, Harvard University, 100 Edwin H. Land Boulevard, Cambridge, MA 02142

General Methods	S2
Synthetic Procedures	S5
Analytical data of the ligands and Cr precursor	S9
Analytical and DFT/TDDFT data of Cr complexes	S18
Computational studies on the doublet energies	S37
Computational studies on the quartet states	S44
fs-Transient absorption spectroscopy	S45
Variable-temperature emission spectroscopy	S49
Photolysis Experiments	S52
Quenching Experiments	S54
References	S57

General Methods

All reactions and measurements were performed under argon atmosphere unless otherwise noted. Gloveboxes (UniLab/MBraun − Ar 5.0, O_2 < 1 ppm, H_2O < 0.1 ppm) were used to store and weight sensitive compounds for synthesis as well as to prepare samples that require the absence of oxygen and/or water. The reagents were purchased from commercial suppliers (Acros Organics, Alfa Aesar, Fischer Scientific and Sigma Aldrich) and used without further purification. Acetonitrile and petroleum ether were dried and distilled from CaH_2. Anhydrous DMF (Sigma Aldrich) was used without further purification. Column chromatography was performed using aluminum oxide (neutral, Brockmann I). Analytical thin layer chromatography (TLC) was done on aluminum oxide F254 (Macherey-Nagel, coated on polyester sheets) and the spots were visualised by ultraviolet light.

NMR Spectra were recorded on a *Bruker Avance II 400* spectrometer at 400.42 MHz (^{1}H) and 100.70 MHz (^{13}C{^{1}H}) in deuterated acetonitrile (Deutero GmbH). All data were evaluated with the software *MestReNova 12.0.4-22023*. The resonances are reported in ppm versus the solvent signal as internal standard (^{1}H; (^{13}C) NMR: actonitrile-d_3: δ = 1.94, 2.13; (1.32, 118.26) ppm)[1] and J values are given in Hz. (s) = singlet, (d) = doublet, (t) = triplet, (q) = quartet.

IR spectra were recorded with a *Bruker Alpha FTIR* spectrometer with an ATR unit containing a diamond crystal.

ESI$^+$ mass spectra were recorded on an *Agilent 6545 HPLC-ESI-QTOF-MS* spectrometer.

Elemental analyses were performed by the central analytic service of the Department of Chemistry of the Johannes Gutenberg University Mainz using an *Elementar vario EL Cube* or by the Mikroanalytisches Labor Kolbe, c/o Fraunhofer Institut UMSICHT, Oberhausen, Germany.

Electrochemical experiments were carried out on a *BioLogic SP-200* voltammetric analyser using platinum wires as *counter* and working electrodes and a 0.01 M Ag/Ag(NO_3) electrode as reference electrode. Cyclic voltammetry and square wave measurements were carried out at scan rates of 100 mV s^{-1} using 0.1 M [^{n}Bu$_4$N][PF$_6$] in CH_3CN as supporting electrolyte. Potentials are referenced against the ferrocenium/ferrocene couple.

UV/VIS/NIR spectra were recorded on an *Agilent Cary 5000* spectrometer using gastight 1.00 cm quartz cuvettes with a Schott valve. Measurements were carried out in acetonitrile (Optima® LC/MS grade, Fisher Scientific). Molar absorption coefficients are given at maximum absorption and for shoulders (highlighted as sh). For deconvolution of NIR absorption band patterns, the spectra were baseline corrected using a biexponential function (eq. S1) to model the tailing of higher energy bands. Deconvolution of the baseline corrected spectra was achieved with five Voigt functions using the *Origin94* software package.

$$\varepsilon(\tilde{\nu}) = \varepsilon_0 + A_1 \mathrm{e}^{\frac{\tilde{\nu}-\tilde{\nu}_0}{t_1}} + A_2 \mathrm{e}^{\frac{\tilde{\nu}-\tilde{\nu}_0}{t_2}} \quad \text{(eq. S1)}$$

Steady-state emission spectra and photoluminescent decay curves of the complexes were measured with a *FLS1000 spectrometer* from *Edinburgh Instruments* equipped with a cooled photomultiplier detector PMT-980. A xenon arc lamp Xe2 (450 W) was used for excitation in steady-state measurements. Time-resolved luminescence experiments were conducted using a pulsed diode laser VPL-450 as excitation source. Absolute luminescence quantum yields Φ were determined using an integrating sphere from *Edinburgh Instruments* with a relative uncertainty estimated as ±10%. Room temperature measurements were done in acetonitrile (Optima® LC/MS grade, Fisher Scientific). Measurements at low temperature were carried out using a liquid nitrogen cooled cryostat *Optistat DN* from *Oxford Instruments* in a 2:3 mixture of methanol (Optima® LC/MS grade) and ethanol (Fisher Scientific). Biexponential Arrhenius fits were performed for the entire measured temperature range on basis of eq. 1 (see main text) justified by the absent phase transition of the solvent mixture.[2] Emission spectra of the ligands were measured with a *spectrofluorometer FS5* from *Edinburgh Instruments*.

fs-Transient absorption spectra were recorded using a setup based on modules supplied by *Light Conversion*. The seed laser of the setup (*Light Conversion* PHAROS, Yb:KGW lasing medium) generates 1030 nm pulses with energy 400 µJ and duration 200 fs at a repetition rate of 50 kHz. The pump beam was generated from the seed in a harmonic generation unit (*Light Conversion* HIRO) *via* nonlinear crystals (beta-barium borate, lithium triborate) with residual fundamental removed by dichroic mirrors within the unit. The pump was passed through an optical chopper (operating at 100 Hz) and a beamsplitter/photodiode combination was used to divide and sort the measurements into pumped and unpumped. The pump was focused onto the sample with an effective beam diameter of roughly 900 µm. The probe beam was generated from the seed laser using supercontinuum generation in a sapphire crystal. The pump-probe delay was controlled over a range of 8 ns by changing probe path length *via* a multipass delay stage. For changing the wavelength region of the generated probe, the seed can be passed through a second harmonic generating medium prior to focusing onto the sapphire. Residual fundamental was filtered out and the white light probe was then focused onto the sample with an effective beam diameter of roughly 400 µm. The probe beam was passed into a grating spectrograph (*Andor Kymera 193i*, grating blaze wavelength 800 nm, 150 lines/mm for VIS-NIR probe and 300 nm, 300 lines/mm for UV-VIS probe) and recorded using a Si NMOS photodiode array detector (256 pixels). A 343 nm pump and 515 nm pump with fluences of 70 µJ cm^{-2} and 3 mJ cm^{-2} were used for **[1^O][OTf]$_3$** and **[1^S][OTf]$_3$**, respectively, with a repetition rate of 1 kHz. Experiments were carried out using a probe generated with 1030 nm fundamental, spanning 500 to 920 nm. The probe pulse arrival time has slight wavelength dependence (roughly 0.5 ps from lowest to highest wavelength), which is

corrected for by applying a wavelength-dependent time offset determined by a polynomial fit to selected points in the coherent artifact at time zero. Samples were measured in 1 mm path length cuvettes, with an OD of OD of 1.29 at 343 nm for **[1^O][OTf]$_3$** and an OD of 0.07 at 515 nm for **[1^S][OTf]$_3$**. No decomposition of the samples due to the TA experiments could be observed via UV/Vis absorption spectroscopy. Chirp correction and global analysis of the TA data using a consecutive kinetic model was performed using the KiMoPack python package.[3] For **[1^S][OTf]$_3$**, the data at time delays <0.5 ps was not included in the global analysis because of the strong artifacts around time zero. For **[1^O][OTf]$_3$**, the ultrafast component with a time constant of 0.03 ps does not carry any physical meaning as it is faster than the excitation pulse width of ca. 200 fs.

Photolysis experiments were conducted by irradiating deaerated stirred solutions of the complexes in inert gas cuvettes. An *Ultra-High Power* collimated LED from *Prizmatix* (UHP-T-460-DI; output power (free): 5.5 W) with a maximum at 456 nm and the centroid at 460 nm was used. The output power was adjusted constant with 1.1 W (20% setting) for all experiments using a *UHPTLCC-02-USB Controller*. The beam of light was focused onto the cuvette with a plano convex lens. At the cuvette, the beam diameter was roughly 1 cm. The temperature of the irradiated solution was kept at 20 °C with a Peltier module. Due to the excitation source being non-monochromatic, the complexes exhibiting a different spectral absorption within the excitation range and the absorbance changing over time upon irradiation, the irradiation and decomposition times of the different complexes are not comparable and only a qualitative comparison can be made.

Stern-Volmer measurements were conducted by titration of deaerated solutions of *trans*-stilbene or anthracene in acetonitrile to deaerated solutions of the corresponding complex in the same solvent in inert gas cuvettes. The titration steps were done in the glovebox. The Stern-Volmer analyses were performed on the basis of eq. S2 and S3.[4]

$$\frac{\tau_0}{\tau} - 1 = K_{SV}[Q] \quad \text{(eq. S2)}$$

$$k_q = \frac{K_{SV}}{\tau_0} \quad \text{(eq. S3)}$$

DFT calculations were performed using the quantum computing suite ORCA 5.0.4.[5,6] Geometry optimisation was performed using (un-)restricted Kohn-Sham orbitals DFT (UKS/RKS) and the B3LYP functional[7-9] in combination with Ahlrich's split valance triple-zeta basis set ZORA-def2-TZVPP[10] for all atoms. Tight convergence criteria were chosen for DFT calculations (keywords *tightscf* and *tightopt*). All DFT calculations make use of the resolution of identity (Split-RI-J) approach for the Coulomb term in combination with the chain-of-spheres approximation for the exchange term (keyword *RIJCOSX*).[11,12] The zeroth order regular approximation was used to describe relativistic effects in all calculations (keyword *ZORA*).[13-19] To account for solvent effects, a conductor-like screening model (keyword *CPCM*(acetonitrile)) modeling acetonitrile was used in all calculations.[20,21] Atom-pairwise dispersion correction was performed with the Becke-Johnson damping scheme (keyword *D3BJ*).[22,23] A numerical frequency calculation confirmed that the optimised geometry corresponds to a minimum structure. Explicit counter ions and/or solvent molecules were neglected. Fifty vertical spin-allowed transitions were calculated by TD-DFT. The charge transfer number analyses of the TD-DFT calculated transitions were done using TheoDORE 2.4.[24] Exited state geometry optimisations were done using TD-DFT and the given state was selected according to the TD-DFT calculation at the initial geometry (keyword *iroot*). To prevent root flipping after a step during the optimization the total overlap between the excited state wavefunctions was calculated and compared with the previous one (keyword *followiroot*). The characters of the optimised geometries were assigned to $^4T_2$, ^{4}LMCT, $^2T_1$ and ^2E states according to the spin densities of the relaxed geometries. Starting from the optimised ground state geometries, distorted $^4T_2$ and ^{4}LMCT states (see main text) could be localised. All calculations were computed on the Elwetritsch supercomputer at RPTU Kaiserslautern-Landau (hpc.rz.rptu.de). This is a member of the AHRP (Alliance for High Performance Computing Rhineland-Palatinate).

CASSCF(x,y)-SC-NEVPT2 calculations of ground and excited state properties with respect to pure metal-centered (MC) states were performed using the complete-active-space self-consistent field (CASSCF) method[25,26] in conjunction with the strongly contracted N-electron valence perturbation theory to second order (SC-NEVPT2)[27-29] in order to recover missing dynamic electron correlation. All electronic states are classified by irreducible representations of the O point group, in spite of the lower actual symmetry of the considered complexes. To figure out the dominant bonding/antibonding orbitals describing the interaction between chromium and the ligand, an active space screening procedure was performed by comparing the results of four different active spaces with the experimental values. The active space (7,12) comprising of five d-orbitals, two occupied σ bonding orbitals and five unoccupied d orbitals of second d-shell[30] give results closest to the experimental values and was considered for all other CASSCF-calculations. 10 quartet and 10 doublet roots were calculated for all CAS calculations.

Active spaces for the active space screening procedure of the CASSCF calculations:

CAS(3,5)	five d orbitals
CAS(3,10)	five d orbitals + five unoccupied d orbitals of second d-shell
CAS(13,10)	five d orbitals + five occupied σ and π bonding orbitals
CAS(7,12)	five d orbitals + two occupied σ bonding orbitals + five unoccupied d orbitals of second d-shell

Crystal structure determinations. Intensity data were collected with a *STOE IPDS-2T* diffractometer and a *STOE STADIVARI* diffractometer from STOE & CIE GmbH with an Oxford cooling using Mo-K_α radiation (λ = 0.71073 Å). The diffraction frames were integrated using the STOE X-Area software package[31] and were corrected for absorption with MULABS[32] of the PLATON[33] software package or with the X-Area software package[31,34,35] (integration or semi-empirical from equivalents. The structures were solved with SHELXT[36] and refined by the full-matrix method based on F^2 using SHELXL[37] of the SHELX[38] software package and the ShelXle[39] graphical interface. All non-hydrogen atoms were refined anisotropically while the positions of all hydrogen atoms were generated with appropriate geometric constraints and allowed to ride on their respective parent atoms with fixed isotropic thermal parameters. Crystallographic data for the structures reported in this paper have been deposited with the Cambridge Crystallographic Data Centre as supplementary publication no. CCDC-2380708 for bptp, CCDC-2380706 for **[1^{O}][OTf]$_3$** and CCDC-2380707 for **[1^{S}][OTf]$_3$**.

Crystallographic Data of bptp: $C_{15}H_{11}N_3S_2$ (297.39); monoclinic; $C2/c$; a = 12.378(3) Å, b = 9.0617(18) Å, c = 12.393(3) Å; α = 90°; β = 99.98(3)°; γ = 90°; V = 1369.0(5) Å^3; Z = 4; density, calcd. = 1.443 g cm^{-3}, T = 120(2) K, μ = 0.380 mm^{-1}; F(000) = 616; crystal size 0.820 × 0.600 × 0.480 mm^3; θ = 3.338 to 27.841°; −16≤h≤14, −11≤k≤11, −16≤l≤16; reflections collected = 3504; reflections unique = 1621 [R(int) = 0.0181]; completeness to θ = 25.242°: 99.6%; absorption correction: integration; max. and min. transmission 0.8808 and 0.7687; data 1621; restraints 0, parameters 92; goodness-of-fit on F^2 = 1.055; final indices [$I > 2\sigma(I)$] R_1 = 0.0348, wR_2 = 0.0905; R indices (all data) R_1 = 0.0409, wR_2 = 0.0948; largest diff. peak and hole 0.419 and −0.284 e Å^{-3}.

Crystallographic Data of [1^{O}][OTf]$_3$: $C_{33}H_{24}CrF_9N_6O_{14}S_3$ (1047.76); monoclinic; $P2_1/n$; a = 15.110(3) Å, b = 13.307(3) Å, c = 19.705(4) Å; α = 90°; β = 98.83(3)°; γ = 90°; V = 3915.1(1) Å^3; Z = 4; density, calcd. = 1.778 g cm^{-3}, T = 120(2) K, μ = 0.573 mm^{-1}; F(000) = 2116; crystal size 0.260 × 0.133 × 0.070 mm^3; θ = 2.395 to 28.175°; −19≤h≤19, −17≤k≤17, −18≤l≤26; reflections collected = 31536; reflections unique = 9445 [R(int) = 0.0948]; completeness to θ = 25.242°: 99.5%; absorption correction: semi-empirical from equivalents; max. and min. transmission 1.0905 and 0.8732; data 9445; restraints 1, parameters 601; goodness-of-fit on F^2 = 1.112; final indices [$I > 2\sigma(I)$] R_1 = 0.0570, wR_2 = 0.1002; R indices (all data) R_1 = 0.1019, wR_2 = 0.1174; largest diff. peak and hole 0.888 and −0.475 e Å^{-3}.

Crystallographic Data of [1^{S}][OTf]$_3$: $C_{35.5}H_{27}CrF_9N_7O_{9.5}S_7$ (1151.06); monoclinic; $P2_1/n$; a = 14.7224(3) Å, b = 13.3391(3) Å, c = 22.8690(5) Å; α = 90°; β = 93.810(2)°; γ = 90°; V = 4481.17(17) Å^3; Z = 4; density, calcd. = 1.706 g cm^{-3}, T = 120(2) K, μ = 0.682 mm^{-1}; F(000) = 2328; crystal size 0.380 × 0.293 × 0.160 mm^3; θ = 2.062 to 31.145°; −19≤h≤20, −18≤k≤19, −30≤l≤32; reflections collected = 84775; reflections unique = 13233 [R(int) = 0.0475]; completeness to θ = 25.242°: 99.9%; absorption correction: semi-empirical from equivalents; max. and min. transmission 0.9168 and 0.0644; data 13233; restraints 139, parameters 793; goodness-of-fit on F^2 = 1.041; final indices [$I > 2\sigma(I)$] R_1 = 0.0420, wR_2 = 0.1142; R indices (all data) R_1 = 0.0618, wR_2 = 0.1213; largest diff. peak and hole 1.568 and −0.675 e Å^{-3}.

Synthetic Procedures

Synthesis of 2,6-bis(2-oxopyridin-1(2*H*)-yl)pyridine[40]

Potassium *tert*-butoxide (4.85 g, 43.2 mmol) was combined with an orange solution of 2-hydroxypyridine (4.11 g, 43.2 mmol) in anhydrous DMF (100 mL). The resulting brown solution was stirred for one hour at room temperature. 2,6-Dibromopyridine (5.12 g, 21.6 mmol) was added in portions and the mixture was heated at 145 °C for 24 hours. After cooling to room temperature, the solvent of the dark brown solution was removed under reduced pressure (5 mbar, 80 °C). The crude residue was suspended in water (100 mL) and the product was extracted with ethyl acetate (3 x 100 mL). The combined organic phases were washed with a saturated aqueous solution of sodium chloride (100 mL), dried with sodium sulfate and filtered. After removing the solvent under reduced pressure (200 mbar, 40 °C), the product was purified by column chromatography on alumina (3:1:0.05 ethyl acetate/methanol/triethylamine, R_f = 0.31) yielding the pale brown solid compound 2,6-bis(2-oxopyridin-1(2*H*)-yl)pyridine (4.19 g, 73%).

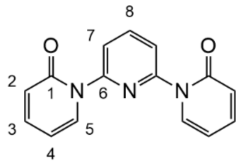

^{1}H NMR (CD_3CN): δ = 8.05 (1 H, dd, $^3J_{HH}$ = 8.4, $^3J_{HH}$ = 7.5, H^8), 7.86 (2 H, d, $^3J_{HH}$ = 7.9, H^7), 7.84 (2 H, ddd, $^3J_{HH}$ = 7.1, $^4J_{HH}$ = 2.1, $^5J_{HH}$ = 0.8, H^5), 7.47 (2 H, ddd, $^3J_{HH}$ = 9.4, $^3J_{HH}$ = 6.5, $^4J_{HH}$ = 2.1, H^3), 6.52 (2 H, ddd, $^3J_{HH}$ = 9.3, $^4J_{HH}$ = 1.3, $^5J_{HH}$ = 0.7, H^2), 6.32 (2 H, ddd, $^3J_{HH}$ = 7.1, $^3J_{HH}$ = 6.5, $^4J_{HH}$ = 1.3, H^4) ppm.

MS (ESI$^+$, CH_3CN): *m/z* (%) = 266.09 (53) [M+H]$^+$, 288.07 (100) [M+Na]$^+$, 553.16 (59) [2 M+Na]$^+$.

IR (ATR): $\tilde{\nu}_{max}$ = 3119 (w), 3089 (w), 1659 (vs, C=O), 1598 (vs), 1576 (vs), 1537 (vs), 1443 (s), 1384 (s), 1289 (s), 1257 (s), 1243 (s), 1145 (s), 1127 (s), 1088 (w), 1007 (w), 991(w), 880 (s), 854 (s), 815 (w), 779 (s), 763 (vs), 746 (s), 730 (w), 686 (w), 616 (w), 575 (s), 537 (w), 522 (s), 474 (w), 464 (w), 429 (w), 409 (w) cm^{-1}.

Synthesis of 2,6-bis(pyridin-2-yloxy)pyridine (bpop)

A solution of 2-bromopyridine (16.7 mL, 171 mmol) in anhydrous DMF (400 mL) was deaerated by purging with argon for half an hour and combined with 2,6-dihydroxypyridine hydrochloride (12.6 g, 85.4 mmol). Addition of potassium carbonate (35.4 g, 256 mmol) to the colourless solution yielded a deep blue suspension. The reaction mixture was heated at 145 °C for 24 hours. After cooling to room temperature, the blue suspension was filtered and the solvent was removed under reduced pressure (5 mbar, 80 °C). The crude residue was suspended in water (400 mL) and the product was extracted with ethyl acetate (3 x 200 mL). The combined organic phases were washed with a saturated aqueous solution of sodium chloride (200 mL), dried with sodium sulfate and filtered. After removing the solvent under reduced pressure (200 mbar, 40 °C), the product was purified by column chromatography on alumina (7:3:0.05 cyclohexane/ethyl acetate/triethylamine, R_f = 0.38) yielding the colourless solid ligand bpop (273 mg, 1.2%).

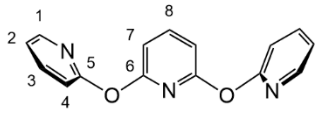

Elemental analysis calcd. for $C_{15}H_{11}N_3O_2$: C, 67.92; H, 4.18; N, 15.84. Found: C, 67.77; H, 4.42; N, 15.80.

^{1}H NMR (CD_3CN): δ = 8.20 (2 H, ddd, $^3J_{HH}$ = 5.0, $^4J_{HH}$ = 2.0, $^5J_{HH}$ = 0.8, H^1), 7.89 (1 H, t, $^3J_{HH}$ = 7.9, H^8), 7.77 (2 H, ddd, $^3J_{HH}$ = 8.2, $^3J_{HH}$ = 7.3, $^4J_{HH}$ = 2.0, H^3), 7.13 (2 H, ddd, $^3J_{HH}$ = 7.3, $^3J_{HH}$ = 4.9, $^4J_{HH}$ = 0.9, H^2), 7.01 (2 H, dt, $^3J_{HH}$ = 8.2, $^4J_{HH}$ = 0.9, H^4), 6.86 (2 H, d, $^3J_{HH}$ = 7.9, H^7) ppm.

^{13}C{^{1}H} NMR (CD_3CN): δ = 162.6 (s, C^5), 161.5 (s, C^6), 148.8 (s, C^1), 143.9 (s, C^8), 140.8 (s, C^3), 121.2 (s, C^2), 114.6 (s, C^4), 109.9 (s, C^7) ppm.

MS (ESI$^+$, CH_3CN): *m/z* (%) = 266.09 (100) [M+H]$^+$, 288.07 (6) [M+Na]$^+$, 553.16 (2) [2 M+Na]$^+$.

IR (ATR): $\tilde{\nu}_{max}$ = 3057 (w), 3009 (w), 1585 (vs), 1567 (vs), 1468 (s), 1441 (s), 1414 (vs), 1286 (s), 1260 (s), 1206 (vs), 1142 (s), 1097 (w), 1075 (w), 1044 (w), 1006 (vs), 991 (vs), 870 (s), 847 (w), 776 (vs), 740 (s), 627 (w), 589 (w), 506 (w), 454 (w), 412 (s) cm^{-1}.

UV/Vis (CH_3CN): λ_{max} (ε) = 267 (11430), 215 (18070) nm (M^{-1} cm^{-1}).

Emission (CH_3CN, room temperature): λ_{max} = 307 nm (λ_{exc} = 267 nm).

Synthesis of 2,6-bis(pyridin-2-ylthio)pyridine[41] (bptp)

Potassium *tert*-butoxide (5.23 g, 46.6 mmol) was combined with a yellow solution of 2-mercaptopyridine (5.18 g, 46.6 mmol) in anhydrous DMF (100 mL). The resulting red solution was stirred for one hour at room temperature. 2,6-Dibromopyridine (5.52 g, 23.3 mmol) was added in portions and the mixture was heated at 145 °C for 24 hours. After cooling to room temperature, the solvent of the dark red solution was removed under reduced pressure (5 mbar, 80 °C). The crude residue was suspended in water (100 mL) and the product was extracted with ethyl acetate (3 x 100 mL). The combined organic phases were washed with a saturated aqueous solution of sodium chloride (100 mL), dried with sodium sulfate and filtered. After removing the solvent under reduced pressure (200 mbar, 40 °C), the product was purified by column chromatography on alumina (7:3:0.05 cyclohexane/ethyl acetate/triethylamine, R_f = 0.28) yielding the pale yellow solid ligand bptp (5.49 g, 79%).

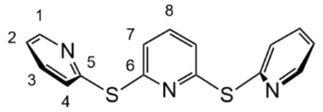

Elemental analysis calcd. for $C_{15}H_{11}N_3S_2$: C, 60.58; H, 3.73; N, 14.13. Found: C, 60.53; H, 3.95; N, 14.12.

^{1}H NMR (CD_3CN): δ = 8.46 (2 H, ddd, $^3J_{HH}$ = 4.8, $^4J_{HH}$ = 1.9, $^5J_{HH}$ = 0.9, H^1), 7.62 (2 H, td, $^3J_{HH}$ = 7.8, $^4J_{HH}$ = 1.9, H^3), 7.59 (1 H, t, $^3J_{HH}$ = 7.8, H^8), 7.40 (2 H, dt, $^3J_{HH}$ = 8.0, $^4J_{HH}$ = 1.0, H^4), 7.27 (2 H, d, $^3J_{HH}$ = 7.8, H^7), 7.21 (2 H, ddd, $^3J_{HH}$ = 7.5, $^3J_{HH}$ = 4.8, $^4J_{HH}$ = 1.1, H^2) ppm.

^{13}C{^{1}H} NMR (CD_3CN): δ = 158.2 (s, C^6), 156.7 (s, C^5), 151.1 (s, C^1), 139.2 (s, C^8), 138.3 (s, C^3), 127.2 (s, C^4), 124.2 (s, C^7), 123.2 (s, C^2) ppm.

MS (ESI^+, CH_3CN): *m/z* (%) = 298.05 (100) $[M+H]^+$, 320.03 (25) $[M+Na]^+$, 617.07 (12) $[2\ M+Na]^+$.

IR (ATR): $\tilde{\nu}_{max}$ = 3042 (w), 2987 (w), 1708 (w), 1571 (s), 1544 (vs), 1448 (s), 1409 (vs), 1279 (w), 1236 (w), 1162 (s), 1141 (s), 1113 (vs), 1083 (s), 1044 (s), 985 (s), 885 (w), 787 (s), 756 (vs), 736 (s), 719 (vs), 655 (w), 618 (w), 593 (w), 514 (w), 461 (w), 420 (w) cm^{-1}.

UV/Vis (CH_3CN): λ_{max} (ε) = 308 (10190), 285 (12600), 244 (16850) nm (M^{-1} cm^{-1}).

Emission (CH_3CN, room temperature): λ_{max} = 349 nm (λ_{exc} = 308 nm).

Synthesis of chromium(III) triflate[42]

Trimethylsilyl trifluormethansulfonate (50.0 g, 225 mmol) was poured into a suspension of purple chromium(III) chloride (3.56 g, 22.5 mmol) in dried acetonitrile (150 mL) and the heterogeneous mixture was heated to 82 °C. To start the reaction, chromium(II) chloride (50.0 mg, 0.407 mmol) was added, changing the colour of the solution to green. Heating the reaction mixture at 82 °C for 24 hours yielded a green-blue solution. After cooling to room temperature, the solvent was removed under reduced pressure (180 mbar, 40 °C). The residue was washed with dry petroleum ether (3 x 50 mL) and dried under reduced pressure (8 x 10^{-3} mbar) for three days. The absence of chloride ions in the green-blue chromium(III) triflate (11.1 g, 99%) was verified by a negative silver(I) salt precipitation with silver(I) triflate in dry acetonitrile.

MS (ESI^+, CH_3CN): *m/z* (%) = 431.90 (17) $[M–OTf+2\ CH_3CN]^+$, 472.92 (5) $[M–OTf+3\ CH_3CN]^+$, 513.95 (11) $[M–OTf+4\ CH_3CN]^+$, 598.88 (16) $[M+H_2O+2\ CH_3CN]^+$, 639.91 (100) $[M+H_2O+3\ CH_3CN]^+$.

IR (ATR): $\tilde{\nu}_{max}$ = 1344 (vs), 1247 (w), 1234 (s), 1178 (vs), 987 (vs), 955 (vs), 824 (w), 767 (w), 624 (vs), 605 (vs), 571 (w), 534 (s), 515 (s), 423 (vs) cm^{-1}.

UV/Vis (CH_3CN): λ_{max} (ε) = 706 (7.3), 592 (51), 430 (35), 311 (17), 222 (4920) nm (M^{-1} cm^{-1}).

Synthesis of [Cr(bpop$_2$)][OTf]$_3$ ([1^O][OTf]$_3$)

A pale yellow solution of bpop (240 mg, 0.905 mmol) in dry acetonitrile (10 mL) was added dropwise to a green-blue solution of chromium(III) triflate (226 mg, 0.453 mmol) in dry acetonitrile (15 mL) without a colour change. Heating the reaction mixture at 82 °C for 2 hours yielded a red solution. After cooling to room temperature, the solvent was removed under reduced pressure (180 mbar, 40 °C) and the red residue was dried under reduced pressure (8 x 10^{-3} mbar) for 24 hours. Washing the residue with THF (4 x 25 mL) by stirring for half an hour each time followed by decanting the solution, separated the yellow solid product from impurities in the red solution. The yellow powder was dried under reduced pressure (8 x 10^{-3} mbar) and dissolved in acetonitrile (2.5 mL). Slow diffusion of cooled diethyl ether (fridge) into the concentrated acetonitrile solution resulted in yellow crystals of **[1^O][OTf]$_3$** (113 mg, 24%) with diffraction quality.

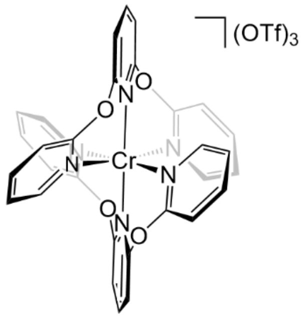

Elemental analysis calcd. for $C_{33}H_{22}CrF_9N_6O_{13}S_3$ x 2 H_2O: C, 37.19; H, 2.46; N, 7.89; S, 9.02. Found: C, 37.18; H, 2.31; N, 7.90; S, 9.03.

MS (ESI$^+$, CH_3CN): *m*/*z* (%) = 194.04 (21) [**1^O**]$^{3+}$, 365.53 (67) [**1^O**+OTf]$^{2+}$, 880.01 (100) [**1^O**+2 OTf]$^+$.

IR (ATR): $\tilde{\nu}_{max}$ = 3119 (w), 3072 (w), 3034 (w), 1612 (vs), 1569 (s), 1483 (s), 1455 (s), 1441 (s), 1425 (vs), 1309 (s), 1240 (vs), 1224 (vs), 1154 (vs), 1108 (s), 1084 (w), 1064 (s), 1028 (vs), 1007 (vs) 871 (s), 846 (s), 789 (s), 755 (w), 729 (w), 677 (w), 658 (w), 636 (vs), 573 (s), 517 (s), 464 (w), 447 (w), 423 (w), 406 (w) cm^{-1}.

UV/Vis (CH_3CN): λ_{max} (ε) = 737 (0.22), 716 (0.35), 689 (0.20), 463 (100), 369 (1640), 268 (17670, sh), 257 (19060), 238 (23480, sh) nm (M^{-1} cm^{-1}).

Emission (CH_3CN, room temperature): λ_{max} = 741, 716 nm (λ_{exc} = 450 nm).

Synthesis of [Cr(bptp$_2$)][OTf]$_3$ ([1^S][OTf]$_3$)

A pale yellow solution of bptp (530 mg, 1.78 mmol) in dry acetonitrile (20 mL) was added dropwise to a green-blue solution of chromium(III) triflate (445 mg, 0.891 mmol) in dry acetonitrile (30 mL) without a colour change. Heating the reaction mixture at 82 °C for 2 hours yielded a red solution. After cooling to room temperature, the solvent was removed under reduced pressure (180 mbar, 40 °C) and the red residue was dried under reduced pressure (8 x 10^{-3} mbar) for 24 hours. Washing the residue with THF (4 x 50 mL) by stirring for half an hour each time followed by decanting the solution separated the yellow solid product from impurities in the red solution. The yellow powder was dried under reduced pressure (8 x 10^{-3} mbar) and dissolved in acetonitrile (5.0 mL). Slow diffusion of cooled diethyl ether (fridge) into the concentrated acetonitrile solution resulted in yellow crystals of **[1^S][OTf]$_3$** (304 mg, 31%) with diffraction quality.

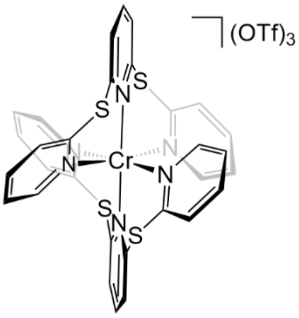

Elemental analysis calcd. for $C_{33}H_{22}CrF_9N_6O_9S_7$: C, 36.23; H, 2.03; N, 7.68; S, 20.51. Found: C, 36.13; H, 2.06; N, 7.66; S, 20.43.

MS (ESI$^+$, CH_3CN): *m*/*z* (%) = 215.34 (37) [**1^S**]$^{3+}$, 397.49 (96) [**1^S**+OTf]$^{2+}$, 943.92 (100) [**1^S**+2 OTf]$^+$.

IR (ATR): $\tilde{\nu}_{max}$ = 3107 (w), 3081 (w), 1593 (s), 1570 (s), 1560 (s), 1544 (w), 1469 (s), 1419 (s), 1378 (w), 1253 (vs), 1224 (vs), 1153 (vs), 1108 (w), 1092 (w), 1067 (w), 1028 (vs), 1009 (s), 808 (w), 776 (s), 756 (w), 739 (w), 725 (s), 690 (w), 636 (vs), 573 (s), 517 (s), 474 (w), 447 (w), 432 (w), 406 (w) cm^{-1}.

UV/Vis (CH_3CN): λ_{max} (ε) = 724 (0.14, sh), 713 (0.43), 683 (0.22), 452 (1880), 426 (1560, sh), 353 (7000, sh), 321 (16290), 299 (18920, sh), 290 (20200), 225 (44560, sh) nm (M^{-1} cm^{-1}).

Emission (CH_3CN, room temperature): λ_{max} = 727, 713 nm (λ_{exc} = 450 nm).

Analytical data of the ligands and precursor

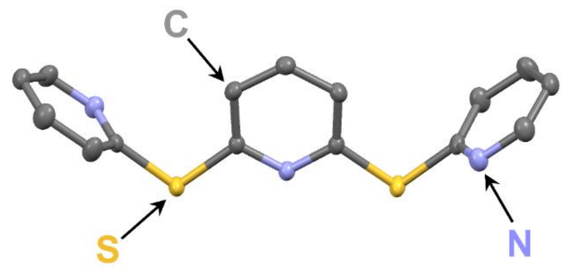

Fig. S1 Molecular structure of bptp in the solid state. Hydrogen atoms omitted, thermal ellipsoids at 50% probability level.

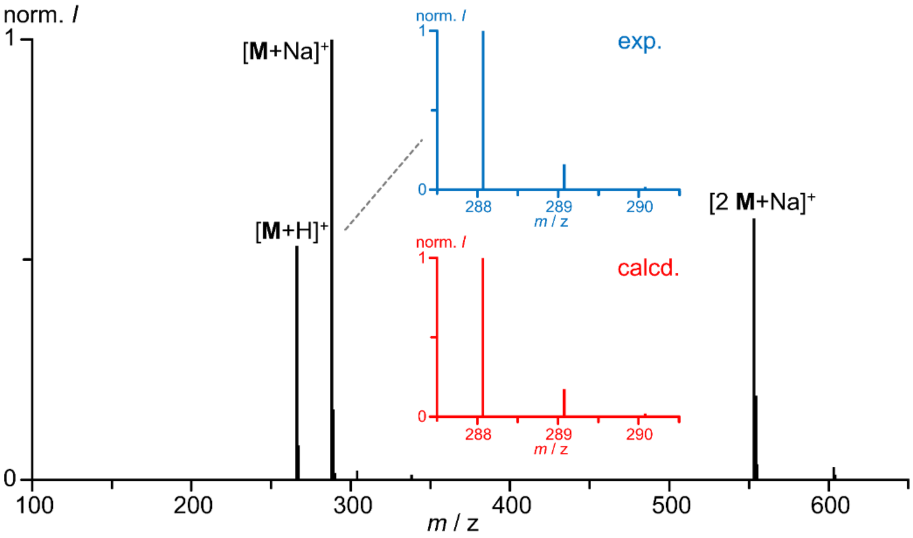

Fig. S2 ESI$^+$ mass spectrum of 2,6-bis(2-oxopyridin-1(2H)-yl)pyridine in CH_3CN with insets of the experimentally found (blue) and calculated (red) isotope pattern for $[M+Na]^+$ ($C_{15}H_{11}N_3NaO_2$).

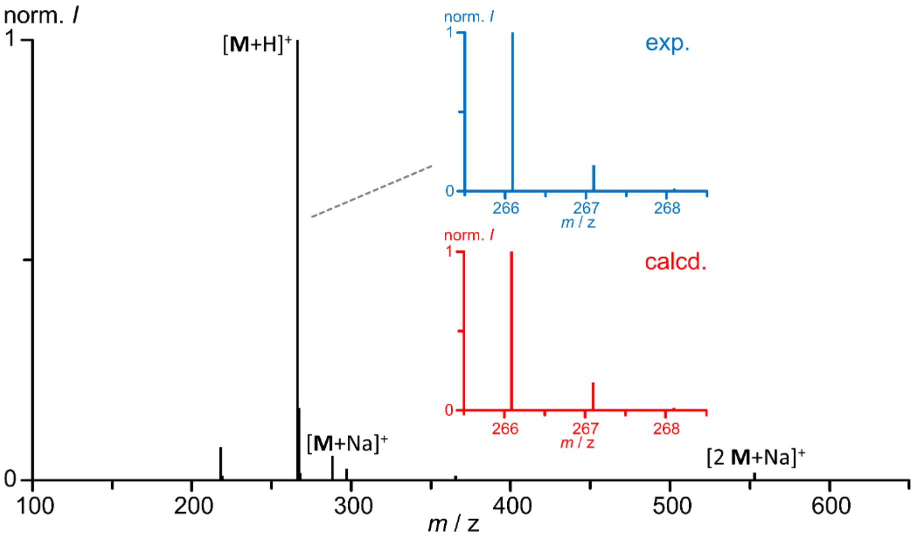

Fig. S3 ESI$^+$ mass spectrum of bpop in CH_3CN with insets of the experimentally found (blue) and calculated (red) isotope pattern for $[M+H]^+$ ($C_{15}H_{12}N_3O_2$).

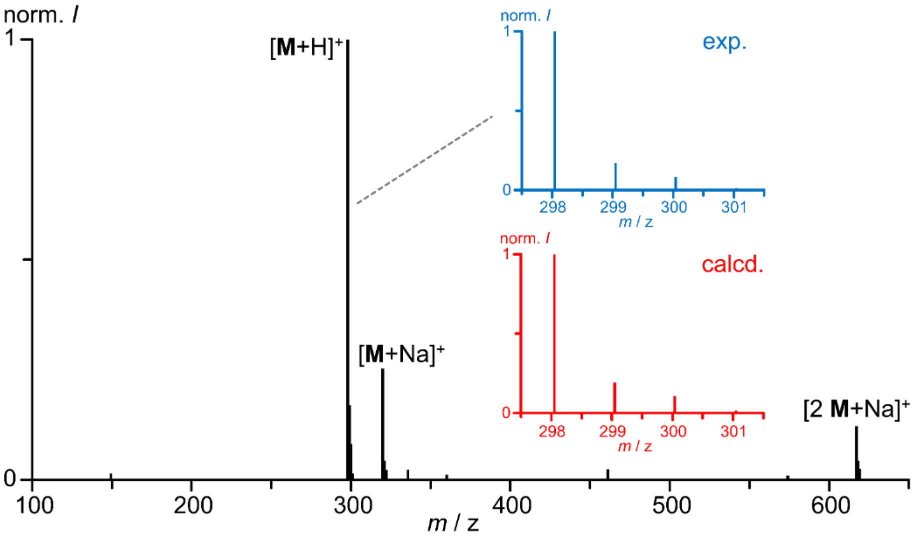

Fig. S4 ESI$^+$ mass spectrum of bptp in CH_3CN with insets of the experimentally found (blue) and calculated (red) isotope pattern for $[M+H]^+$ ($C_{15}H_{12}N_3S_2$).

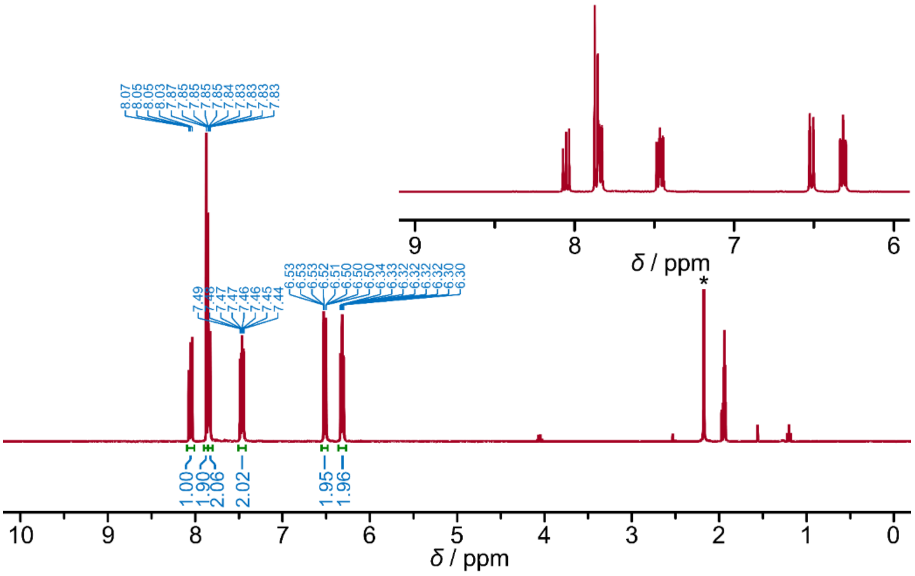

Fig. S5 ^1H NMR spectrum of 2,6-bis(2-oxopyridin-1(2H)-yl)pyridine in CD_3CN with inset of the aromatic region. The asterisk denotes the water peak.

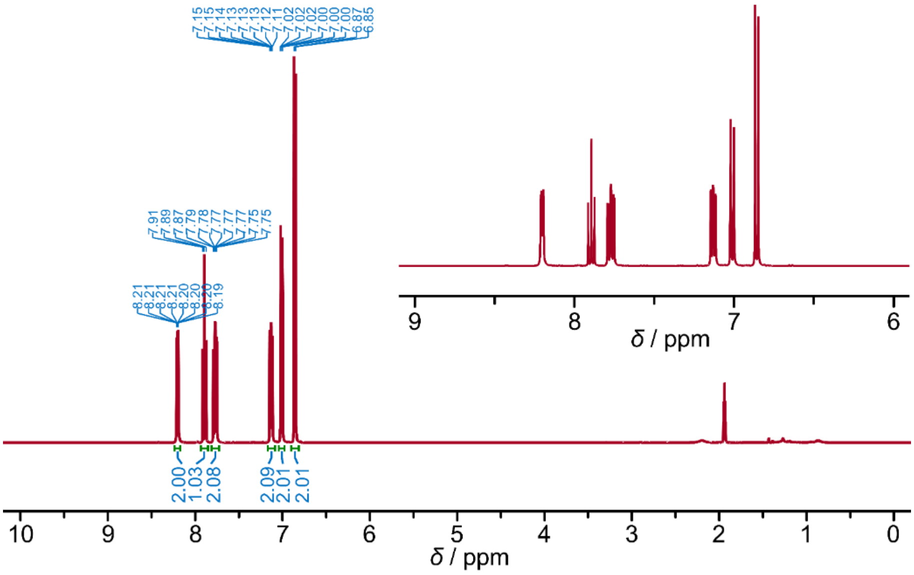

Fig. S6 ^{1}H NMR spectrum of bpop in CD_3CN with inset of the aromatic region.

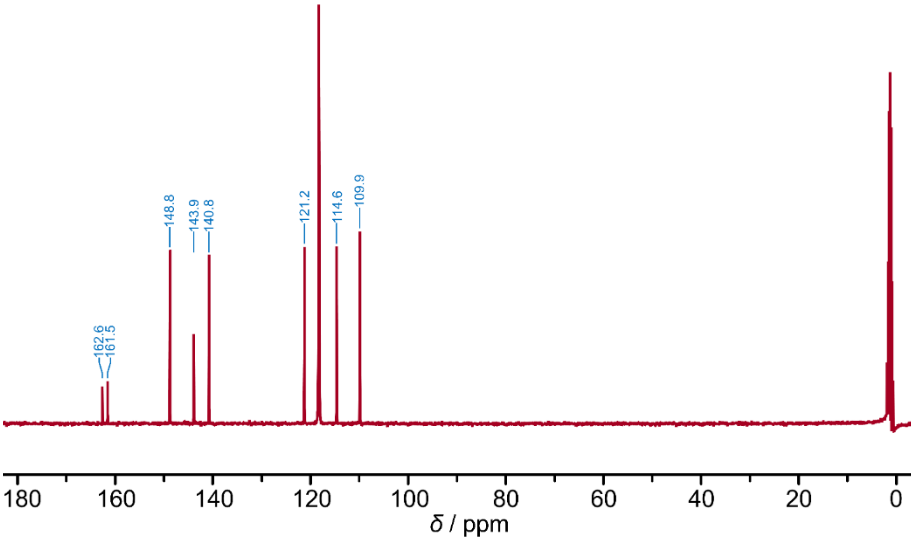

Fig. S7 $^{13}C\{^{1}H\}$ NMR spectrum of bpop in CD_3CN.

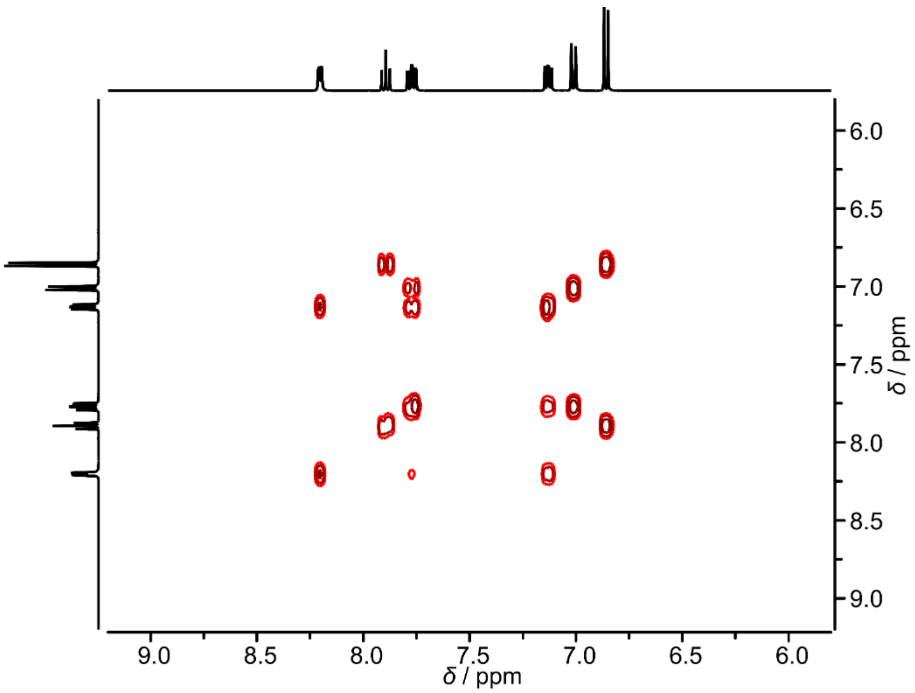

Fig. S8 $^{1}H,^{1}H$ COSY of bpop in CD_3CN.

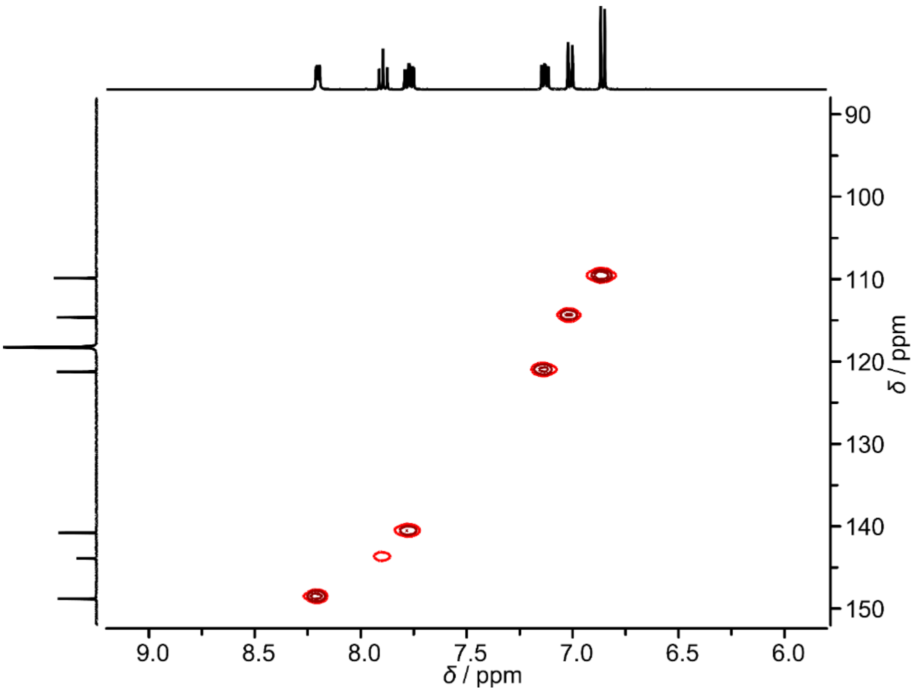

Fig. S9 $^{13}C,^{1}H$ HSQC of bpop in CD_3CN.

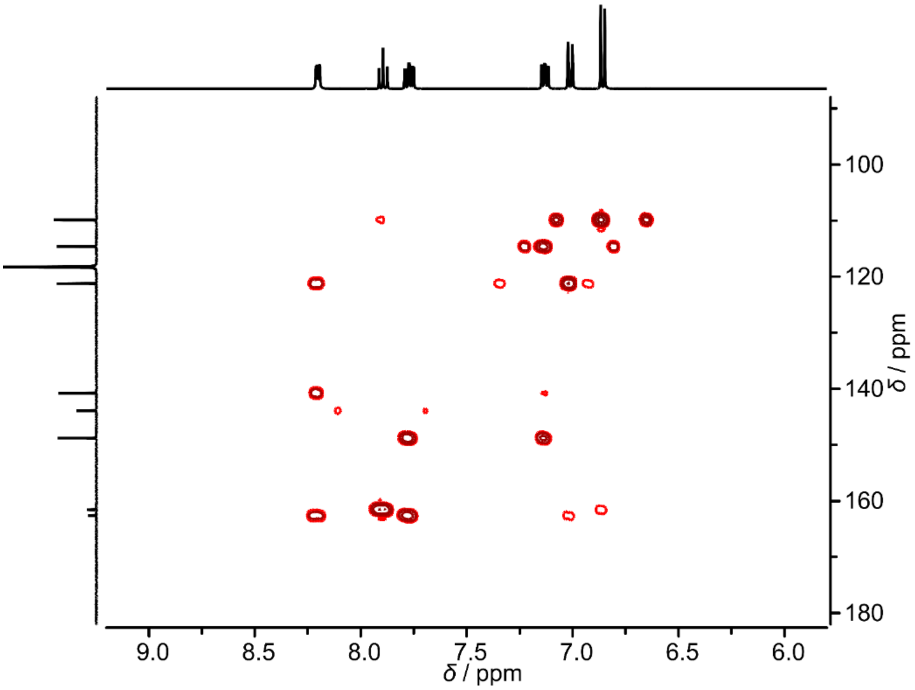

Fig. S10 $^{13}C,^{1}H$ HMBC of bpop in CD_3CN.

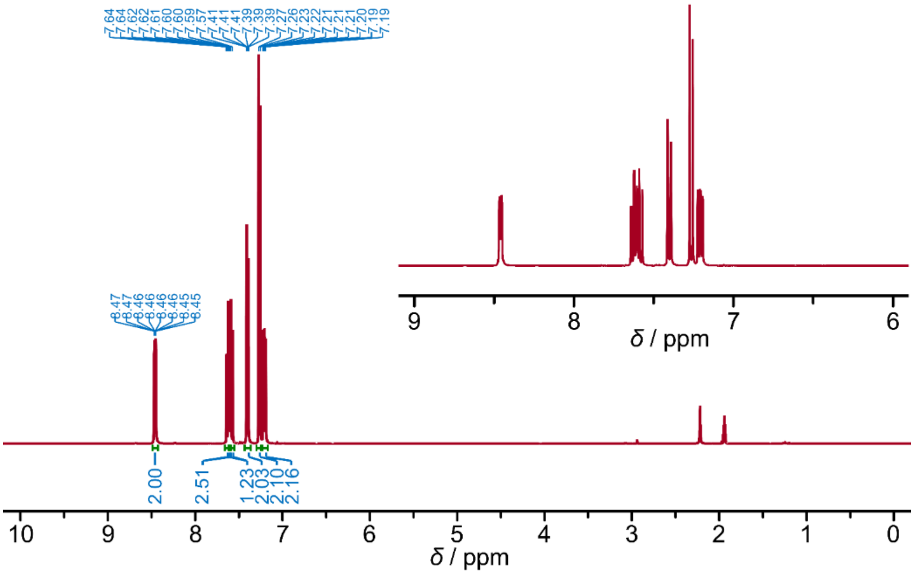

Fig. S11 ^{1}H NMR spectrum of bptp in CD_3CN with inset of the aromatic region.

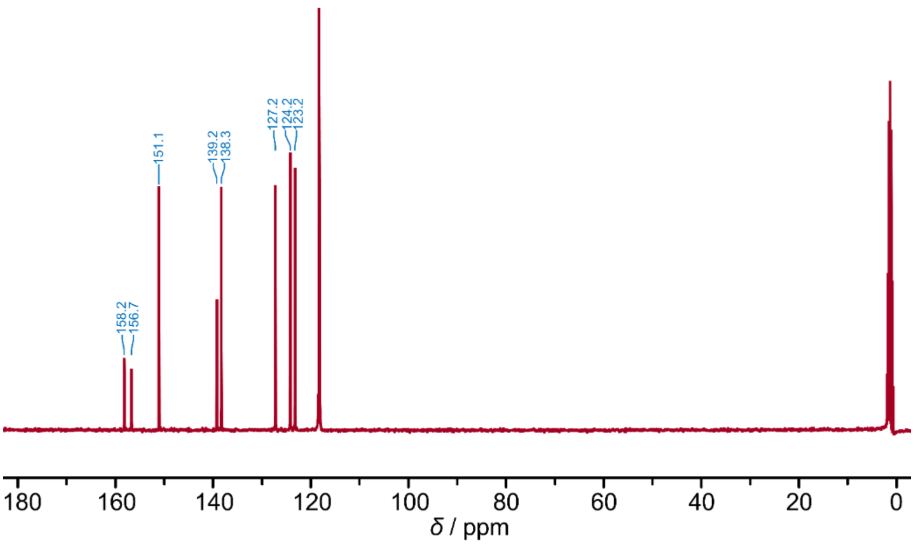

Fig. S12 $^{13}C\{^{1}H\}$ NMR spectrum of bptp in CD_3CN.

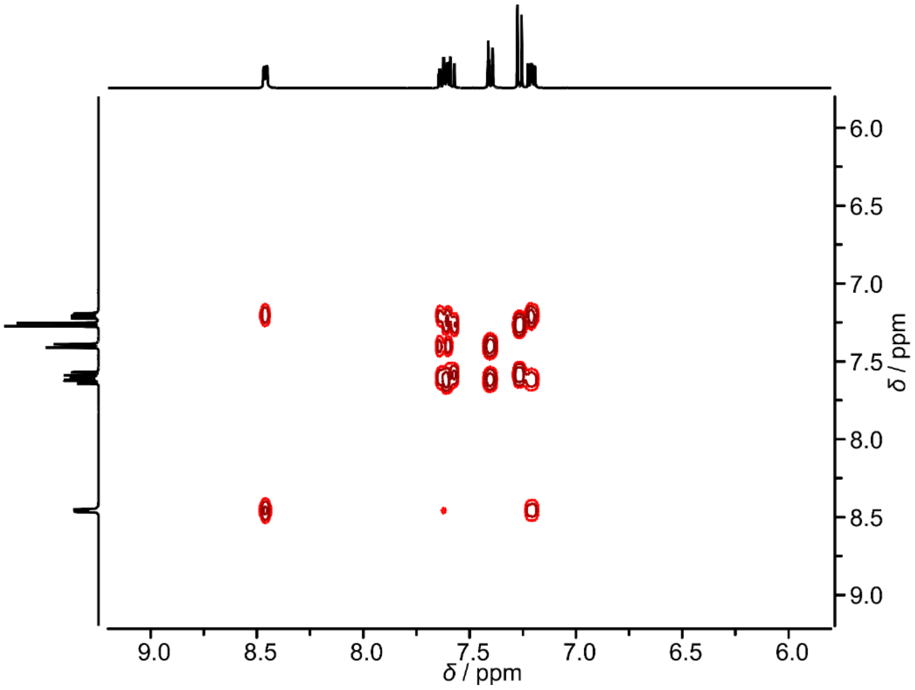

Fig. S13 $^{1}H,^{1}H$ COSY of bptp in CD_3CN.

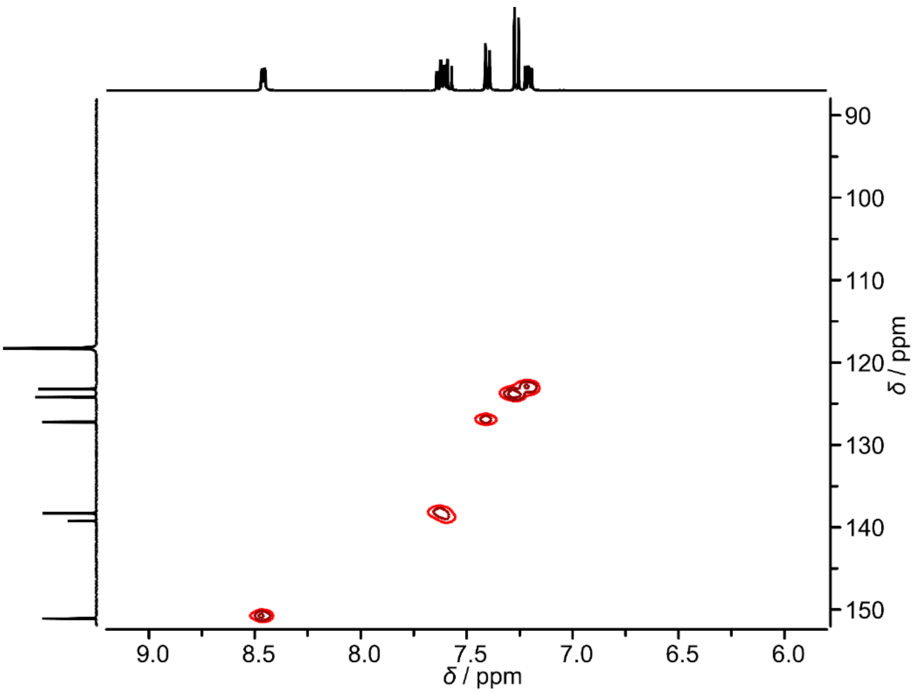

Fig. S14 $^{13}C,^{1}H$ HSQC of bptp in CD_3CN.

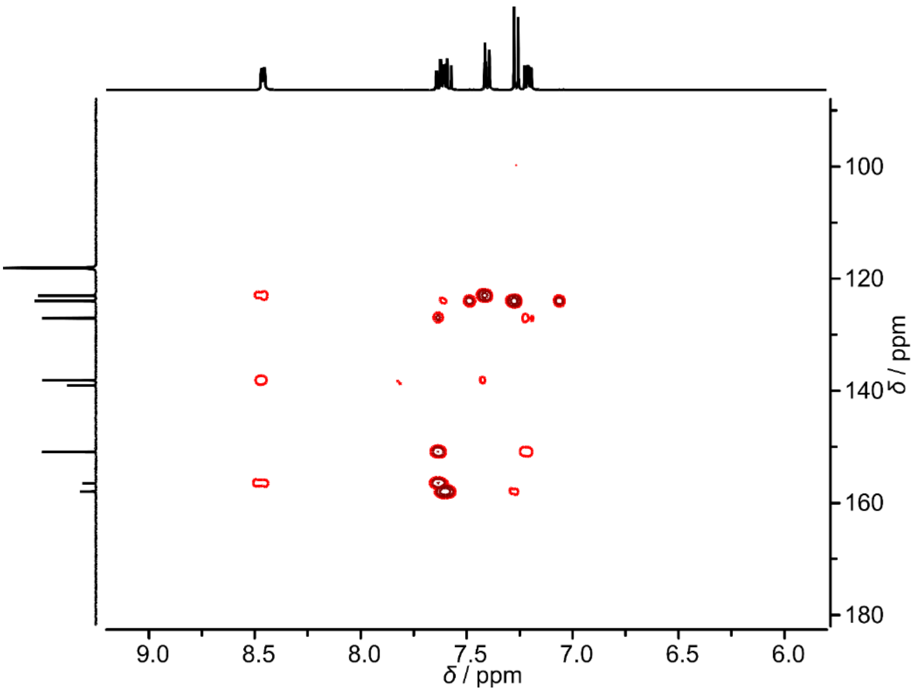

Fig. S15 $^{13}C,^{1}H$ HMBC of bptp in CD_3CN.

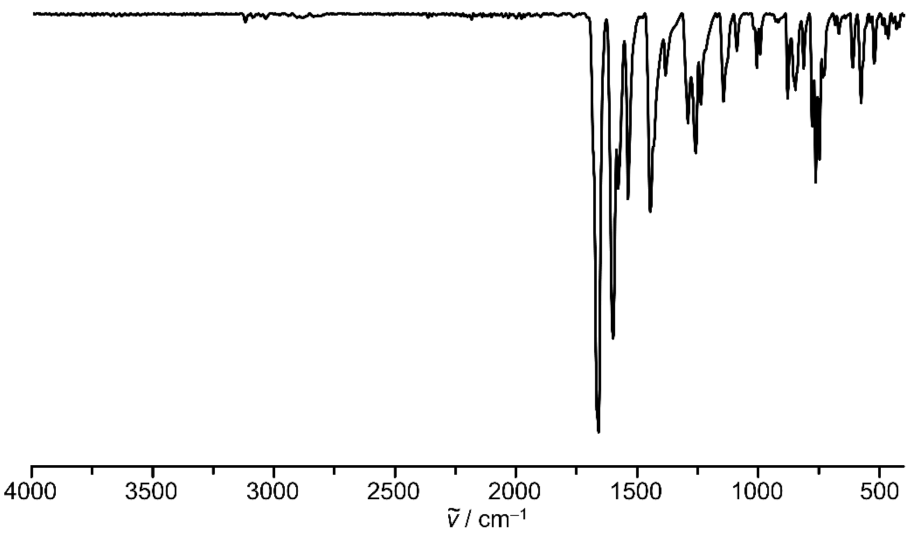

Fig. S16 ATR-IR spectrum of 2,6-bis(2-oxopyridin-1(2H)-yl)pyridine.

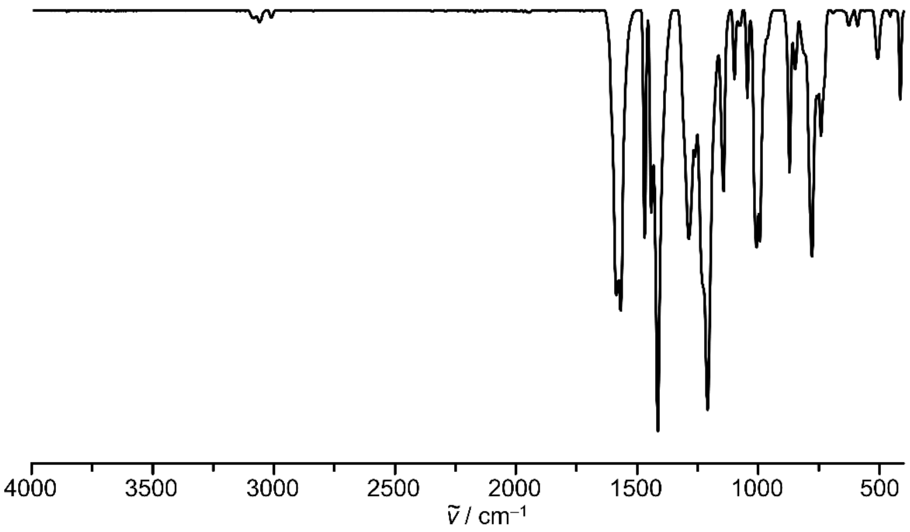

Fig. S17 ATR-IR spectrum of bpop.

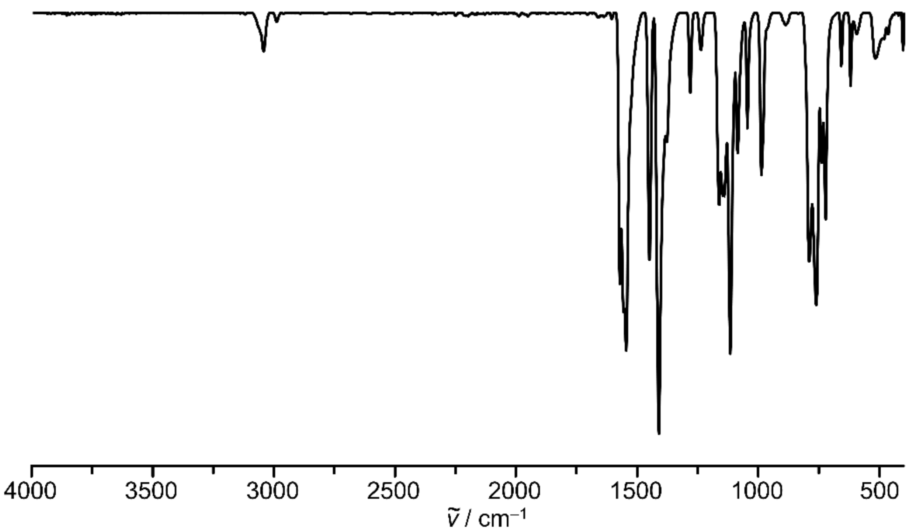

Fig. S18 ATR-IR spectrum of bptp.

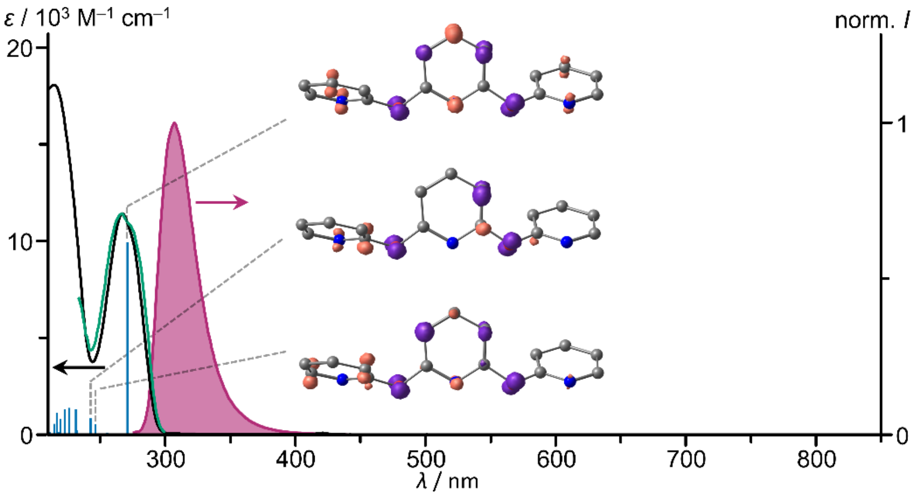

Fig. S19 UV/VIS/NIR absorption spectrum (black), excitation spectrum (λ_{em} = 307 nm, green) and emission spectrum (λ_{exc} = 267 nm, purple) of bpop in deaerated acetonitrile at room temperature, TD-DFT calculated oscillator strengths (blue) and difference electron densities (isosurface value 0.01 a.u.) of three low energy transitions.

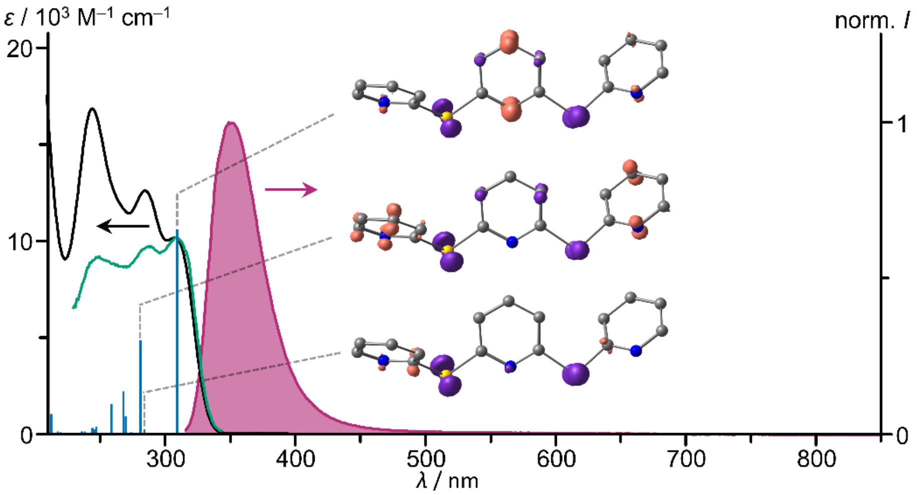

Fig. S20 UV/VIS/NIR absorption spectrum (black), excitation spectrum (λ_{em} = 351 nm, green) and emission spectrum (λ_{exc} = 308 nm, purple) of bptp in deaerated acetonitrile at room temperature, TD-DFT calculated oscillator strengths (blue) and difference electron densities (isosurface value 0.01 a.u.) of three low energy transitions.

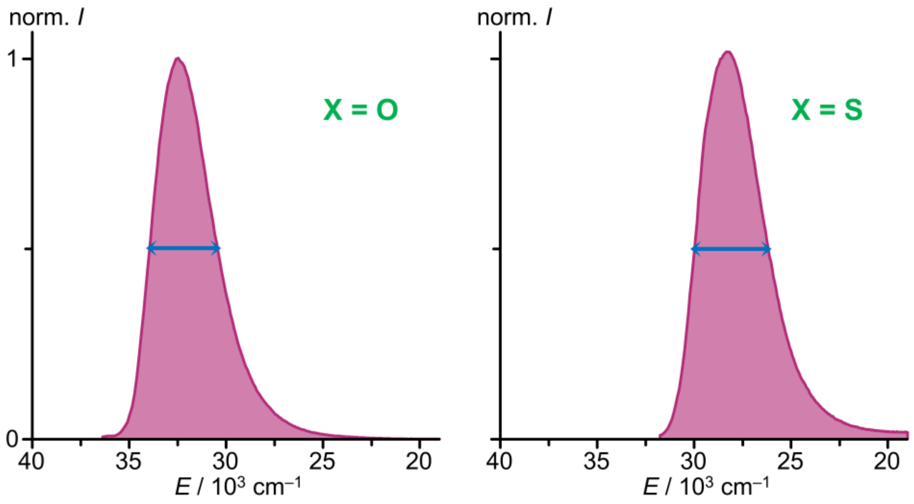

Fig. S21 Normalised emission spectra (purple) of the ligands bpop (λ_{exc} = 267 nm) and bptp (λ_{exc} = 308 nm) in acetonitrile and illustration of the full width at half maximum (FWHM).

Analytical data of Cr compounds

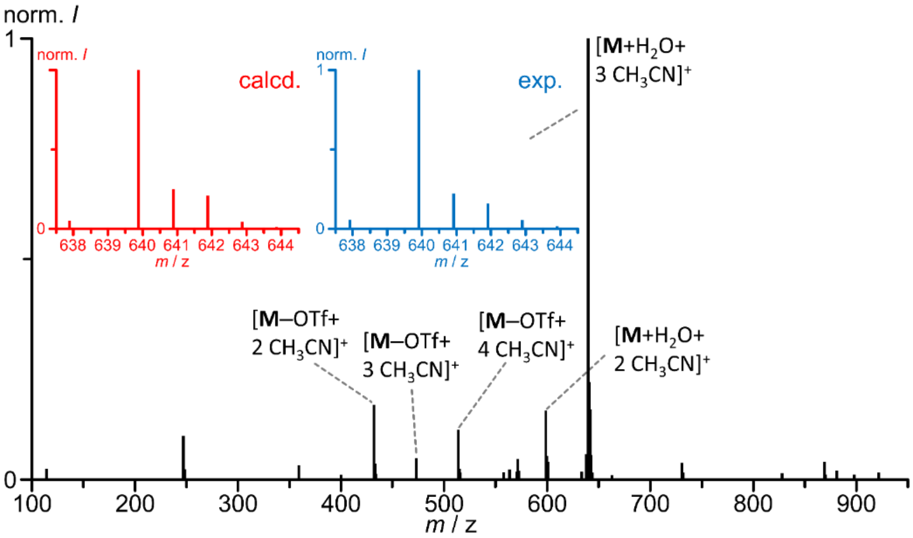

Fig. S22 ESI$^+$ mass spectrum of Cr(OTf)$_3$ in CH_3CN with insets of the experimentally found (blue) and calculated (red) isotope pattern for [M+H_2O+3 CH_3CN]$^+$ ($C_9H_{11}CrF_9N_3O_{10}S_3$).

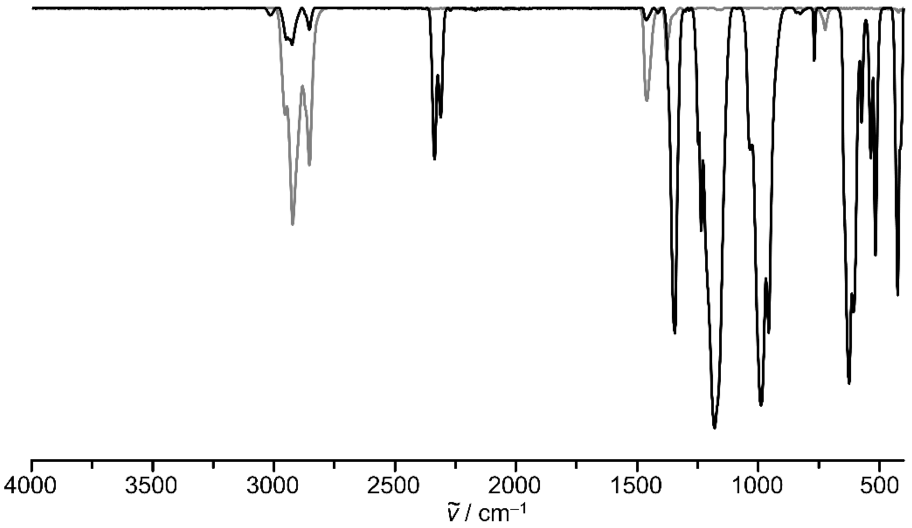

Fig. S23 ATR-IR spectrum of Cr(OTf)$_3$ as suspension in nujol (black) and ATR-IR spectrum of nujol (grey).

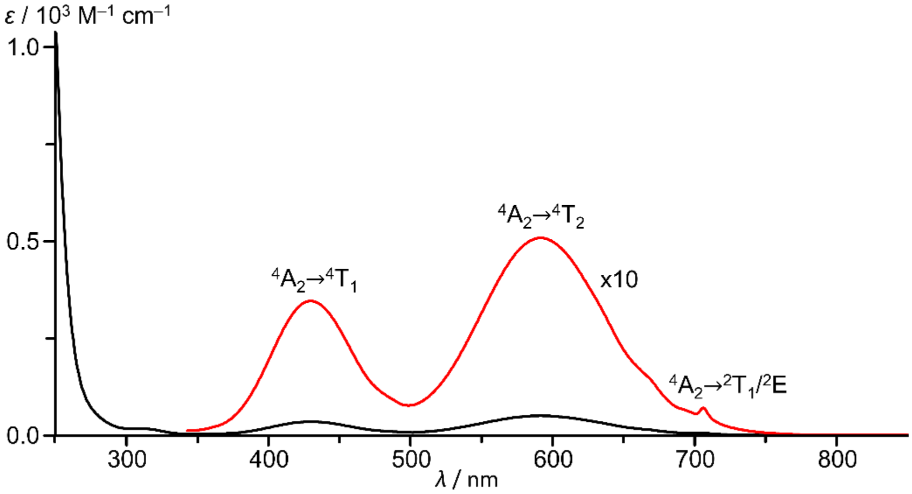

Fig. S24 UV/VIS/NIR absorption spectrum (black) of $Cr(OTf)_3$ in dry acetonitrile at room temperature and zoomed region of the ligand field bands (red).

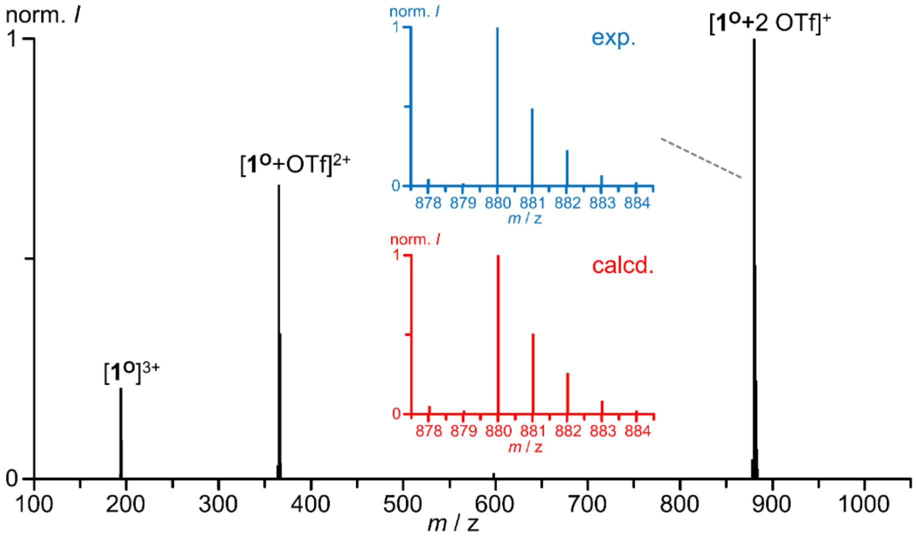

Fig. S25 ESI$^+$ mass spectrum of **[1^O][OTf]$_3$** in CH_3CN with insets of the experimentally found (blue) and calculated (red) isotope pattern for [**1**^{O}+2 OTf]$^+$ ($C_{32}H_{22}CrF_6N_6O_{10}S_2$).

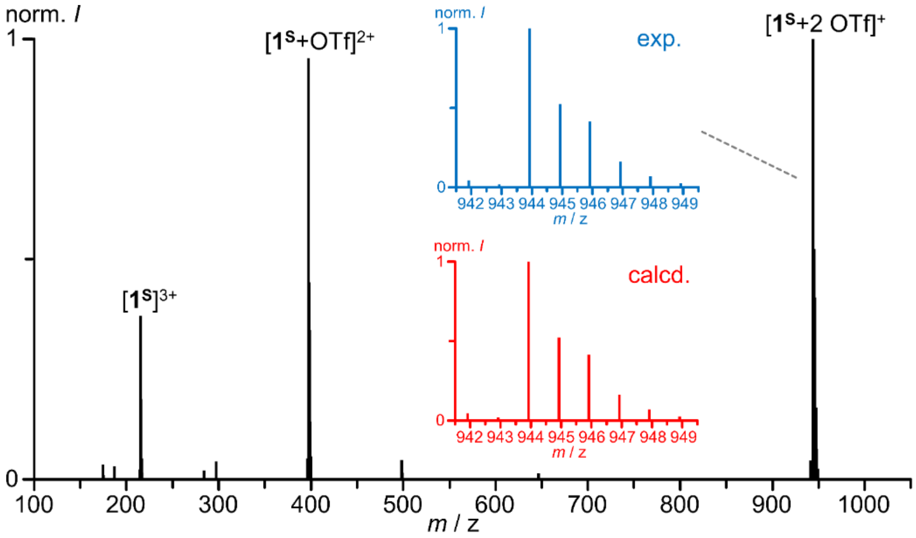

Fig. S26 ESI$^+$ mass spectrum of **[1^S][OTf]$_3$** in CH_3CN with insets of the experimentally found (blue) and calculated (red) isotope pattern for [**1**^{S}+2 OTf]$^+$ ($C_{32}H_{22}CrF_6N_6O_6S_6$).

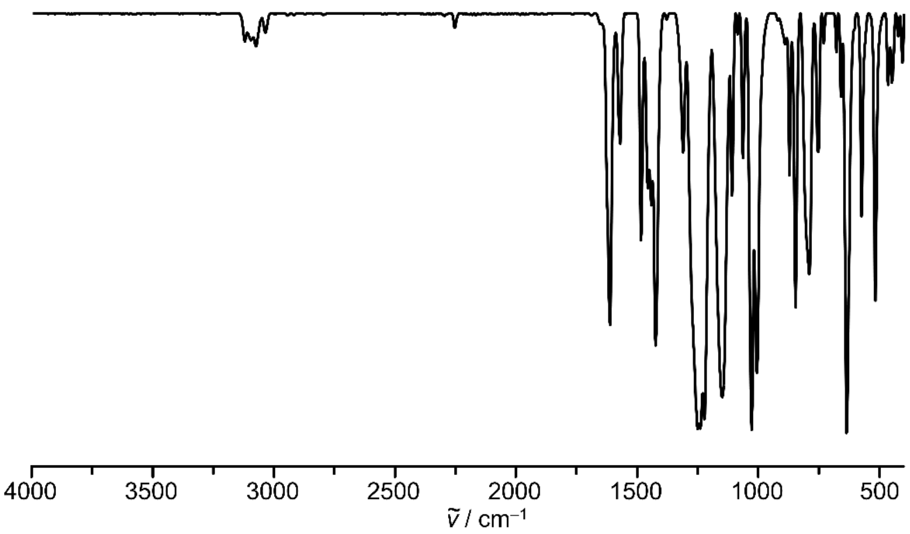

Fig. S27 ATR-IR spectrum of **[1[O]][OTf]₃**.

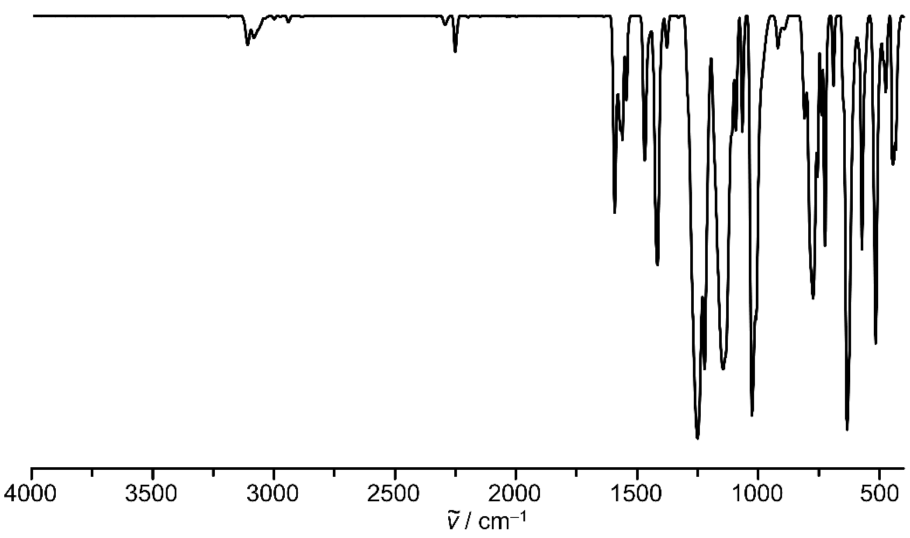

Fig. S28 ATR-IR spectrum of **[1[S]][OTf]₃**.

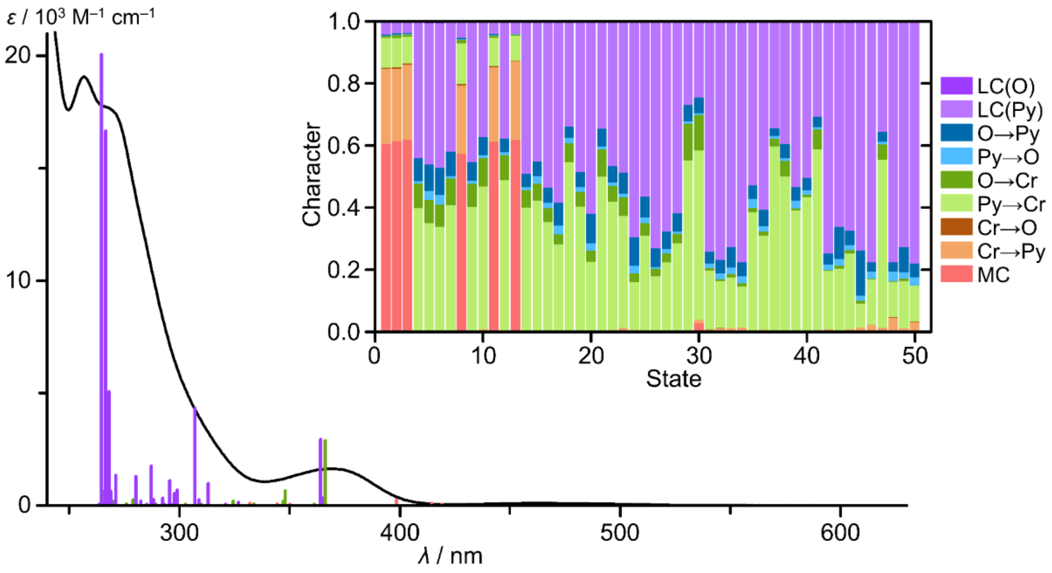

Fig. S29 UV/VIS absorption spectrum of **[1^{O}][OTf]$_3$** in CH_3CN (black) with TD-DFT calculated oscillator strengths of **[1^{O}]$^{3+}$** coloured according to the most dominant character of the corresponding transition (red: MC, green: LMCT, purple: LC). Inset: Charge transfer number analysis of the 50 lowest energy spin-allowed transitions calculated by Loewdin population analysis with the complex cation **[1^{O}]$^{3+}$** fragmented into chromium (Cr), the pyridine system (Py) and the oxygen atoms (O).

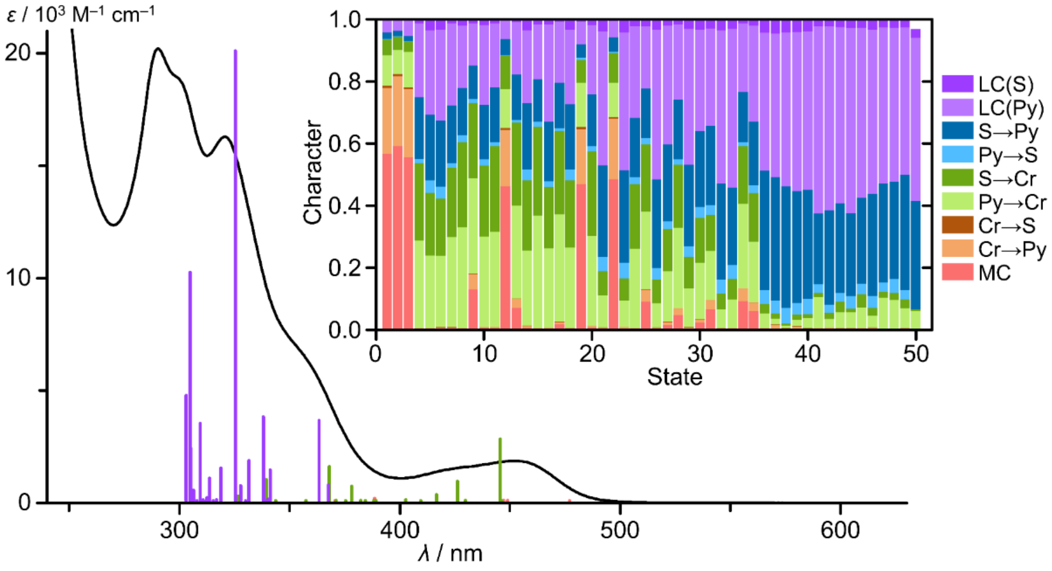

Fig. S30 UV/VIS absorption spectrum of **[1^{S}][OTf]$_3$** in CH_3CN (black) with TD-DFT calculated oscillator strengths of **[1^{S}]$^{3+}$** coloured according to the most dominant character of the corresponding transition (red: MC, green: LMCT, purple: LC). Inset: Charge transfer number analysis of the 50 lowest energy spin-allowed transitions calculated by Loewdin population analysis with the complex cation **[1^{S}]$^{3+}$** fragmented into chromium (Cr), the pyridine system (Py) and the sulphur atoms (S).

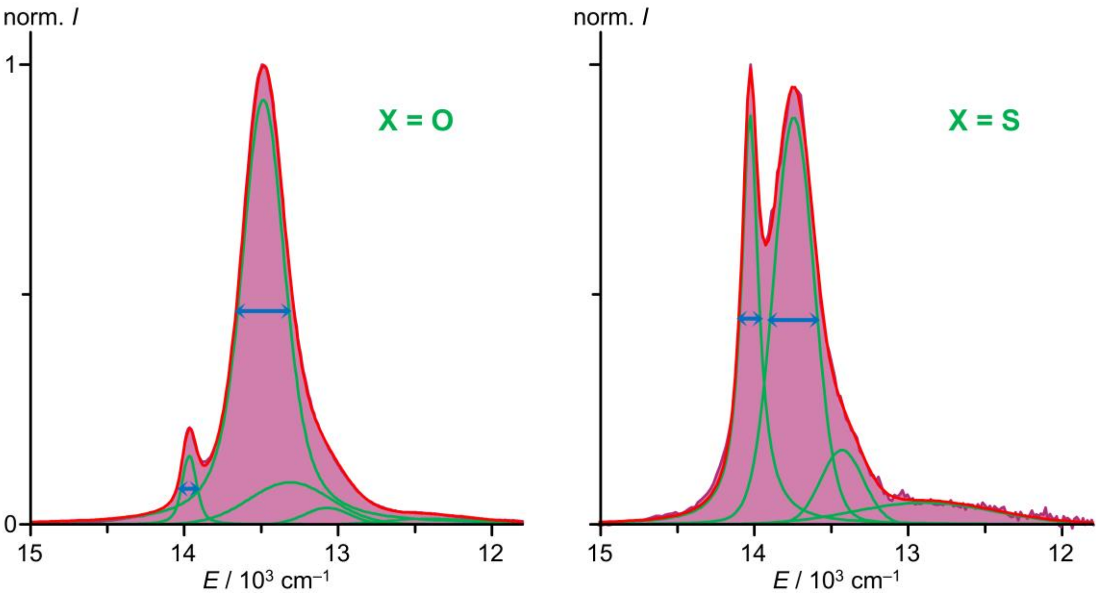

Fig. S31 Normalised emission spectra (purple) of the complexes **[1^{O}]$^{3+}$** (λ_{exc} = 450 nm) and **[1^{S}]$^{3+}$** (λ_{exc} = 450 nm) in acetonitrile at 293 K fitted with Voigt functions (green) in a sum fit (red) used for determining the full width at half maximum (FWHM).

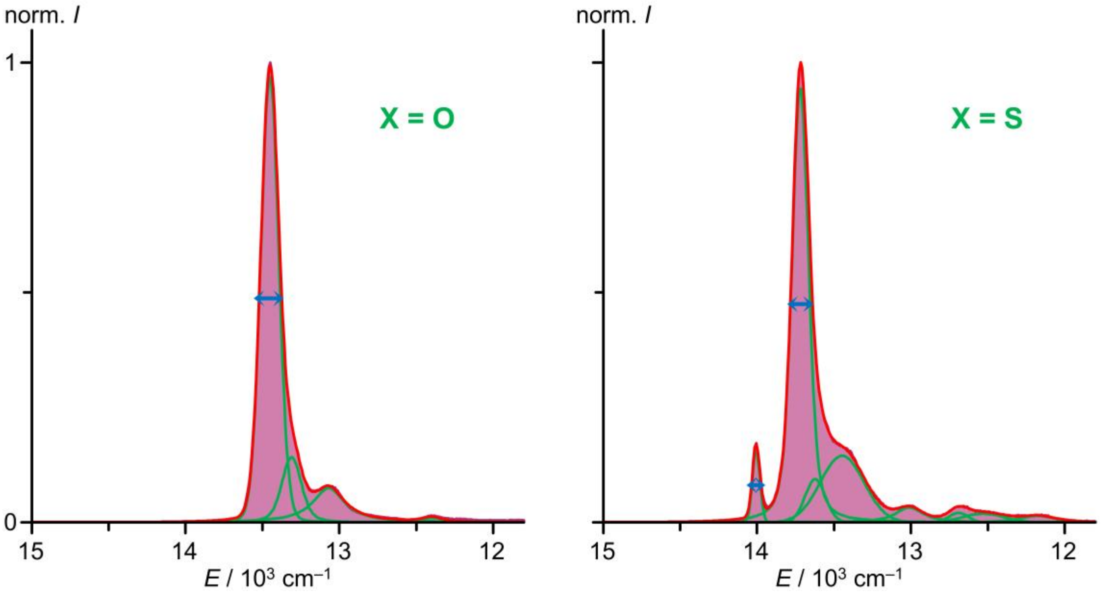

Fig. S32 Normalised emission spectra (purple) of the complexes **[1^{O}]$^{3+}$** (λ_{exc} = 450 nm) and **[1^{S}]$^{3+}$** (λ_{exc} = 450 nm) in ethanol/methanol (3:2) at 77 K fitted with Voigt functions (green) in a sum fit (red) used for determining the full width at half maximum (FWHM).

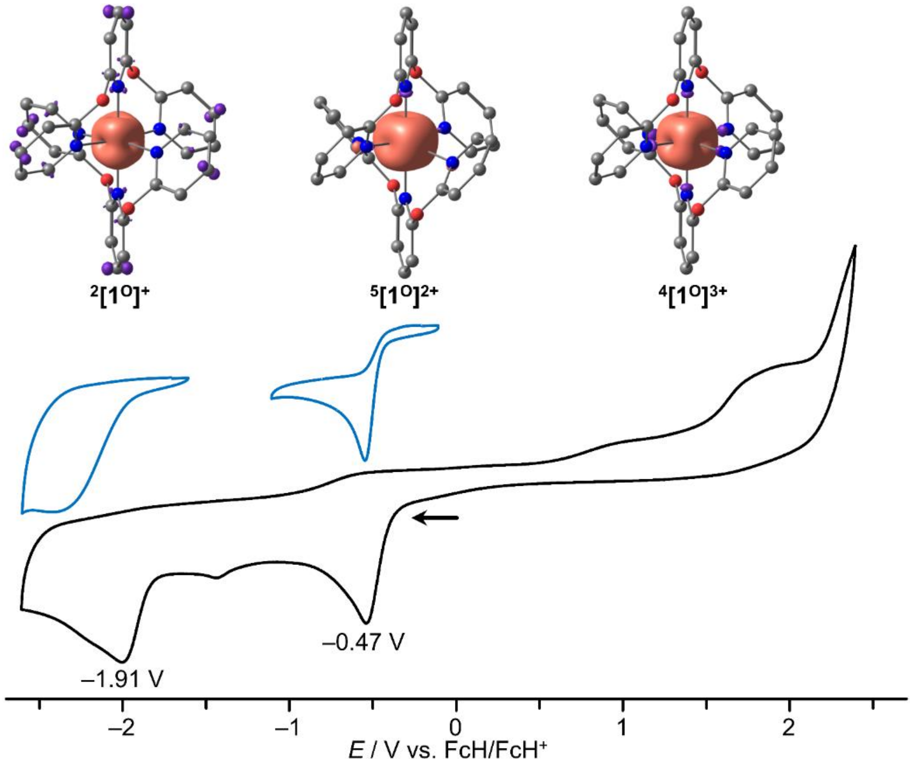

Fig. S33 Cyclic voltammograms of 3 mM **[1^O][OTf]₃** in a 100 mM solution of [$^{n}Bu_4N$][PF_6] in CH_3CN over the full range (peak potentials E_p against FcH^+/FcH) and in the range of the reduction waves and calculated spin densities (contour value: 0.01 a.u.) of the thermodynamically most stable reduced species.

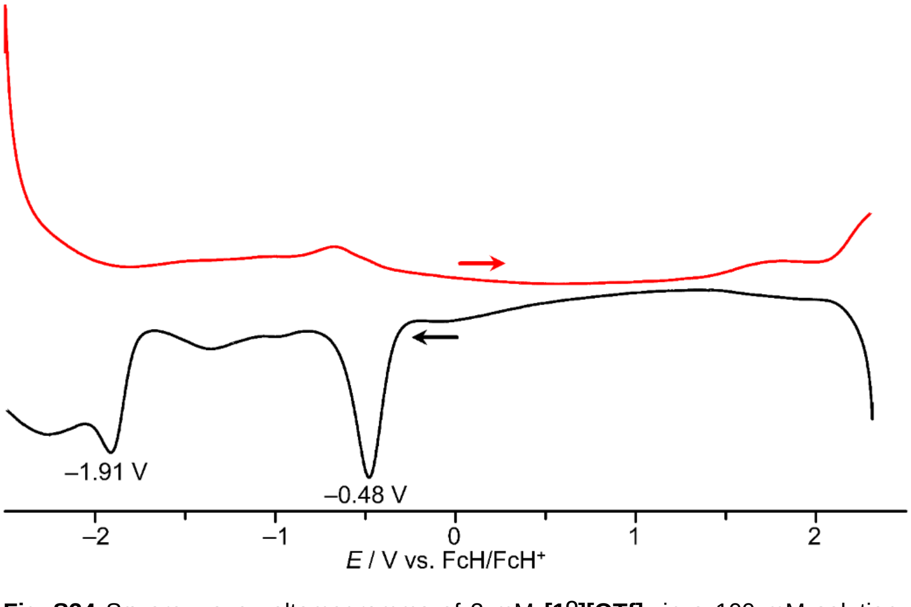

Fig. S34 Square wave voltamogramms of 3 mM **[1^{O}][OTf]$_3$** in a 100 mM solution of [$^{n}Bu_4N$][PF_6] in CH_3CN (peak potentials E_p against FcH^+/FcH).

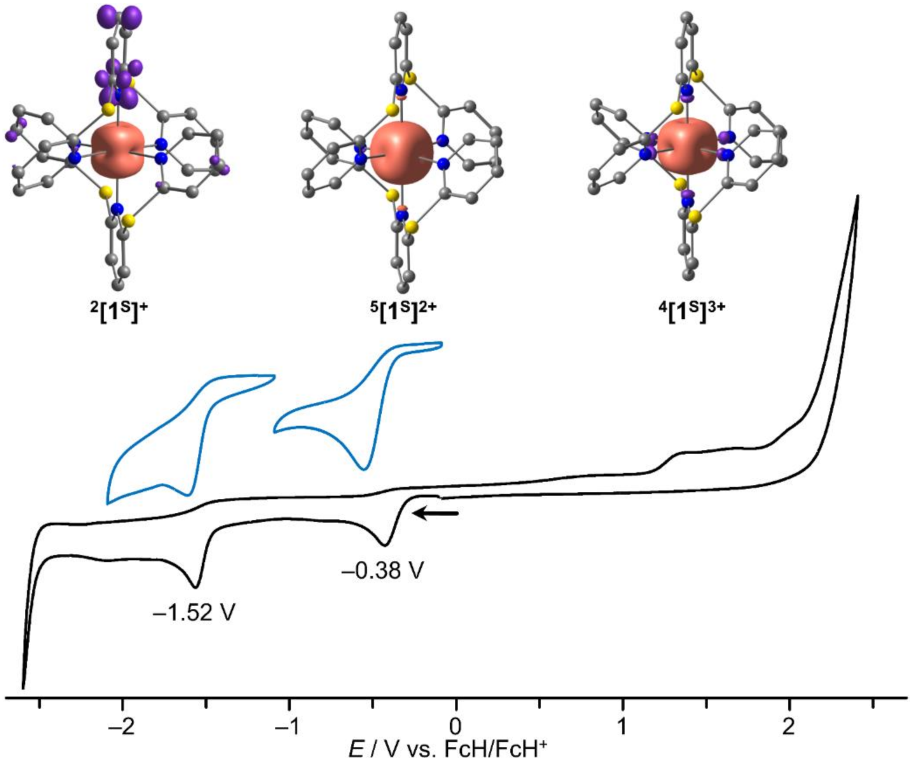

Fig. S35 Cyclic voltammograms of 3 mM **[1^S][OTf]$_3$** in a 100 mM solution of [^{n}Bu$_4$N][PF$_6$] in CH_3CN over the full range (peak potentials E_p against FcH$^+$/FcH) and in the range of the reduction waves and calculated spin densities (contour value: 0.01 a.u.) of the thermodynamically most stable reduced species.

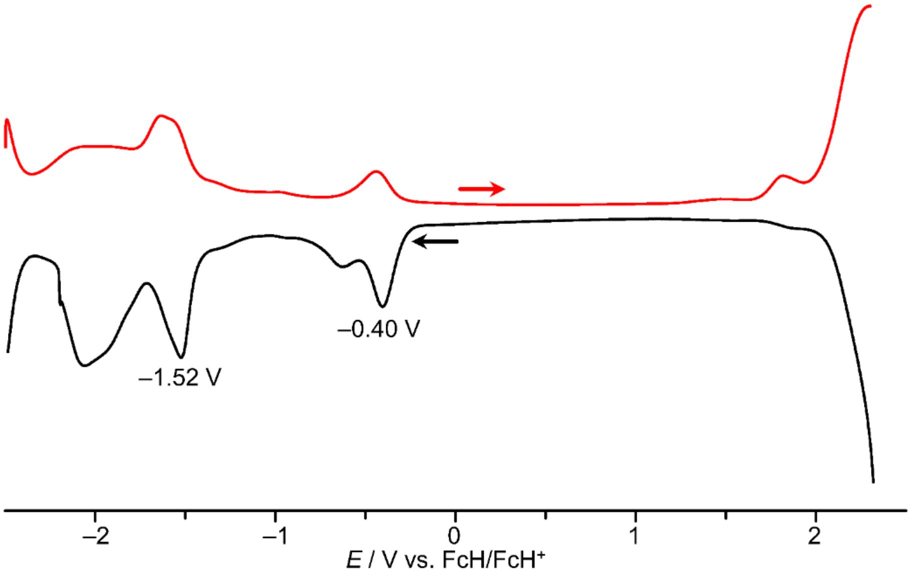

Fig. S36 Square wave voltamogramms of 3 mM **[1^S][OTf]$_3$** in a 100 mM solution of [^{n}Bu$_4$N][PF$_6$] in CH_3CN (peak potentials E_p against FcH$^+$/FcH).

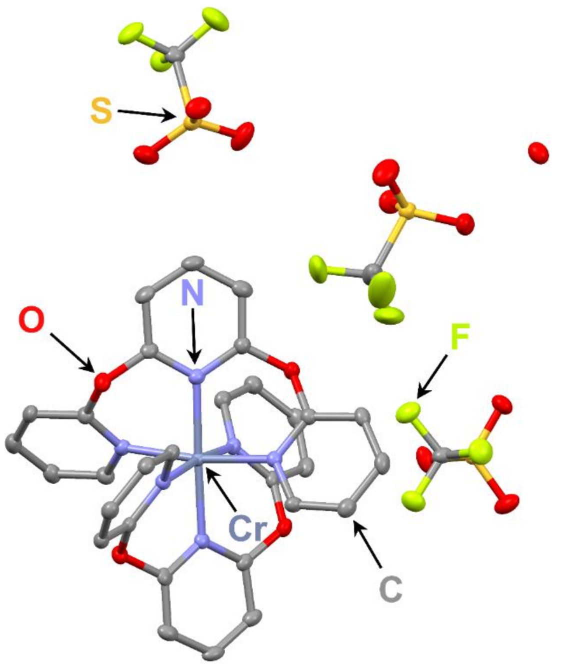

Fig. S37 Molecular structure of **[1^{O}][OTf]$_3$xH$_2$O** in the solid state. Hydrogen atoms omitted, thermal ellipsoids at 50% probability level.

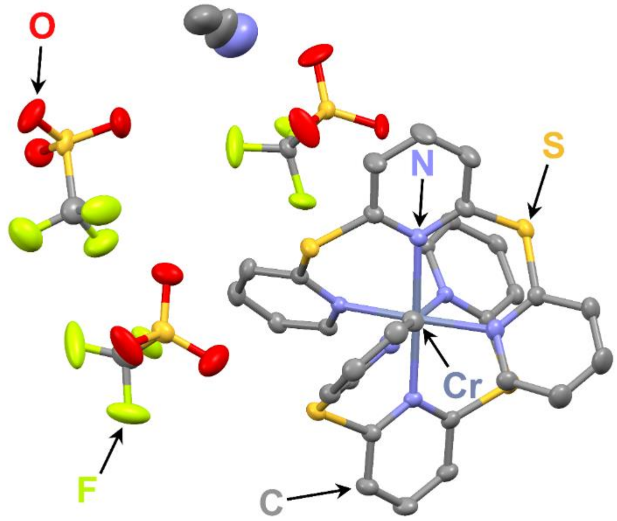

Fig. S38 Molecular structure of **[1^{S}][OTf]$_3$xCH$_3$CN** in the solid state. Hydrogen atoms omitted, thermal ellipsoids at 50% probability level.

Table S1 Structure parameters of the complex series **[1^X]$^{3+}$** from crystal structure determination (XRD) and DFT geometry optimisation with an exemplary structure illustration.[43,44] Bond lengths are given in Å and angles in °.

Complex	Method	Parameter				Graphical illustration
		d (Cr–N_c^1)	d (Cr–N_c^2)			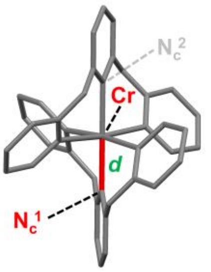
**[1^S]$^{3+}$**	XRD	2.1006(16)	2.0868(16)			
	DFT	*2.1400*	*2.1399*			
**[1^O]$^{3+}$**	XRD	2.0391(28)	2.0377(28)			
	DFT	*2.0510*	*2.0507*			
**[1^{NMe}]$^{3+}$**	XRD	2.0405(18)	2.0358(18)			
	DFT	*2.0463*	*2.0460*			
**[1^{CH2}]$^{3+}$**	XRD	2.0771(13)	2.0599(13)			
	DFT	*2.1127*	*2.1119*			
		d (Cr–N_t^1)	d (Cr–N_t^2)	d (Cr–N_t^3)	d (Cr–N_t^4)	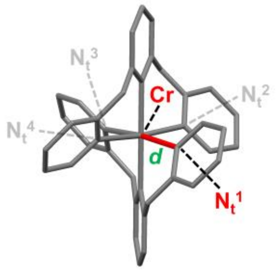
**[1^S]$^{3+}$**	XRD	2.0852(17)	2.0785(16)	2.0723(16)	2.0639(16)	
	DFT	*2.1178*	*2.1180*	*2.1178*	*2.1180*	
**[1^O]$^{3+}$**	XRD	2.0601(26)	2.0479(31)	2.0380(27)	2.0419(31)	
	DFT	*2.0707*	*2.0705*	*2.0707*	*2.0705*	
**[1^{NMe}]$^{3+}$**	XRD	2.0530(18)	2.0418(19)	2.0414(18)	2.0309(18)	
	DFT	*2.0580*	*2.0576*	*2.0578*	*2.0575*	
**[1^{CH2}]$^{3+}$**	XRD	2.0886(13)	2.0755(13)	2.0703(13)	2.0691(13)	
	DFT	*2.1128*	*2.1112*	*2.1100*	*2.1128*	
		$\measuredangle$ ($N_c^1CrN_c^2$)	α ($N_t^1CrN_t^3$)	α ($N_t^2CrN_t^4$)		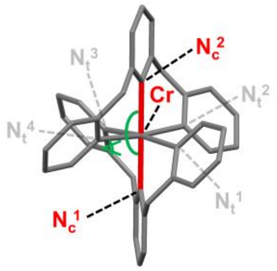
**[1^S]$^{3+}$**	XRD	178.83(7)	177.38(7)	178.15(7)		
	DFT	*179.99*	*178.65*	*178.69*		
**[1^O]$^{3+}$**	XRD	178.24(12)	172.30(12)	172.82(12)		
	DFT	*180.00*	*173.99*	*173.94*		
**[1^{NMe}]$^{3+}$**	XRD	179.90(9)	171.98(7)	171.78(7)		
	DFT	*179.88*	*172.97*	*173.06*		
**[1^{CH2}]$^{3+}$**	XRD	178.58(4)	177.47(4)	177.65(4)		
	DFT	*179.72*	*176.56*	*176.74*		
		β ($N_t^1CrN_t^2$)	$\measuredangle$ ($N_t^2CrN_t^3$)	β ($N_t^3CrN_t^4$)	$\measuredangle$ ($N_t^4CrN_t^1$)	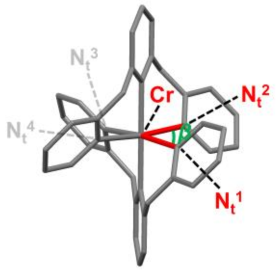
**[1^S]$^{3+}$**	XRD	85.70(7)	96.75(7)	84.97(6)	92.59(7)	
	DFT	*85.58*	*94.44*	*85.58*	*94.43*	
**[1^O]$^{3+}$**	XRD	89.63(11)	90.65(11)	89.79(11)	90.90(11)	
	DFT	*90.06*	*90.27*	*90.06*	*90.25*	
**[1^{NMe}]$^{3+}$**	XRD	90.44(7)	91.27(7)	89.08(7)	90.35(7)	
	DFT	*88.99*	*91.53*	*88.93*	*91.40*	
**[1^{CH2}]$^{3+}$**	XRD	85.08(5)	93.04(5)	84.63(5)	97.25(5)	
	DFT	*84.82*	*95.02*	*84.86*	*95.49*	

Complex	Method	Parameter				Graphical illustration
		∡ ($N_c^1CrN_t^1$)	∡ ($N_c^1CrN_t^2$)	∡ ($N_c^1CrN_t^3$)	∡ ($N_c^1CrN_t^4$)	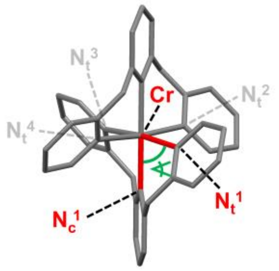
$[1^S]^{3+}$	XRD	89.30(7)	89.42(6)	89.81(6)	91.26(6)	
	DFT	*89.33*	*90.65*	*89.33*	*90.66*	
$[1^O]^{3+}$	XRD	86.00(11)	94.62(12)	86.31(11)	92.56(12)	
	DFT	*86.99*	*93.03*	*87.00*	*93.03*	
$[1^{NMe}]^{3+}$	XRD	86.00(7)	93.90(7)	86.07(7)	94.32(8)	
	DFT	*86.50*	*93.39*	*86.47*	*93.56*	
$[1^{CH2}]^{3+}$	XRD	89.69(5)	89.11(5)	88.59(5)	91.20(5)	
	DFT	*88.26*	*91.65*	*88.31*	*91.60*	
		∡ ($N_c^2CrN_t^1$)	∡ ($N_c^2CrN_t^2$)	∡ ($N_c^2CrN_t^3$)	∡ ($N_c^2CrN_t^4$)	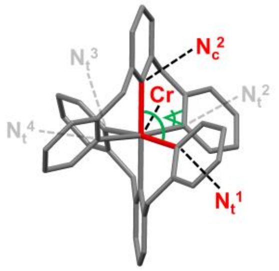
$[1^S]^{3+}$	XRD	91.44(7)	89.73(6)	89.50(6)	89.61(6)	
	DFT	*90.68*	*89.35*	*90.67*	*89.35*	
$[1^O]^{3+}$	XRD	92.78(11)	86.63(11)	94.92(11)	86.19(12)	
	DFT	*93.01*	*86.97*	*93.00*	*86.97*	
$[1^{NMe}]^{3+}$	XRD	94.08(7)	86.04(7)	93.85(7)	85.74(8)	
	DFT	*93.56*	*86.52*	*93.46*	*86.54*	
$[1^{CH2}]^{3+}$	XRD	90.68(5)	89.55(5)	91.00(5)	90.11(5)	
	DFT	*92.02*	*88.34*	*91.41*	*88.41*	
		a ($C^1–X^1$)	*a* ($C^2–X^1$)	*a* ($C^3–X^2$)	*a* ($C^4–X^2$)	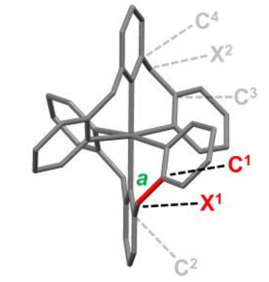
$[1^S]^{3+}$	XRD	1.7916(27)	1.7686(21)	1.7637(19)	1.7661(20)	
	DFT	*1.7642*	*1.7697*	*1.7642*	*1.7695*	
$[1^O]^{3+}$	XRD	1.3702(41)	1.3777(40)	1.3841(36)	1.3787(42)	
	DFT	*1.3677*	*1.3617*	*1.3677*	*1.3618*	
$[1^{NMe}]^{3+}$	XRD	1.3971(28)	1.4022(29)	1.3866(28)	1.4114(29)	
	DFT	*1.3901*	*1.4009*	*1.3904*	*1.4007*	
$[1^{CH2}]^{3+}$	XRD	1.5014(21)	1.5074(19)	1.5015(19)	1.5092(19)	
	DFT	*1.4999*	*1.5045*	*1.4998*	*1.5044*	
		a ($C^5–X^3$)	*a* ($C^6–X^3$)	*a* ($C^7–X^4$)	*a* ($C^8–X^4$)	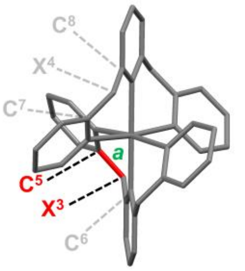
$[1^S]^{3+}$	XRD	1.7634(19)	1.7688(21)	1.7573(19)	1.7589(21)	
	DFT	*1.7642*	*1.7697*	*1.7643*	*1.7695*	
$[1^O]^{3+}$	XRD	1.3736(45)	1.3815(43)	1.3746(44)	1.3687(44)	
	DFT	*1.3677*	*1.3617*	*1.3678*	*1.3618*	
$[1^{NMe}]^{3+}$	XRD	1.3917(28)	1.3961(29)	1.3914(28)	1.4070(29)	
	DFT	*1.3910*	*1.4006*	*1.3906*	*1.4009*	
$[1^{CH2}]^{3+}$	XRD	1.5041(19)	1.5147(18)	1.5051(21)	1.5145(18)	
	DFT	*1.5003*	*1.5049*	*1.4997*	*1.5044*	
		∡ ($C^1X^1C^2$)	∡ ($C^3X^2C^4$)	∡ ($C^5X^3C^6$)	∡ ($C^7X^4C^8$)	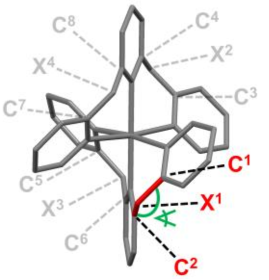
$[1^S]^{3+}$	XRD	103.29(12)	102.83(9)	104.08(9)	104.58(9)	
	DFT	*104.44*	*104.45*	*104.44*	*104.45*	
$[1^O]^{3+}$	XRD	122.10(26)	118.16(26)	119.36(27)	123.34(28)	
	DFT	*122.47*	*122.45*	*122.48*	*122.44*	
$[1^{NMe}]^{3+}$	XRD	122.03(19)	121.39(19)	122.13(19)	122.79(18)	
	DFT	*122.30*	*122.34*	*122.25*	*122.25*	
$[1^{CH2}]^{3+}$	XRD	112.04(11)	117.90(11)	118.65(12)	117.20(11)	
	DFT	*115.14*	*115.33*	*115.13*	*115.00*	

Table S2 Structure parameters of the complex series **[1^{X}]$^{3+}$** from crystal structure determination (XRD) and DFT geometry optimisation each with an exemplary structure illustration.[43,44] The planes Py^{n} (n = 1–6) or CrN_4 were calculated by a least-square fit and contain the corresponding pyridine ring or the meridional coordinating nitrogen atoms and the chromium centre. Angles are given in °.

Complex	Method	Parameter		Graphical illustration
		ϕ (Py^1, Py^2)		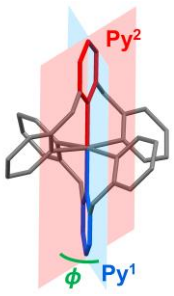
**[1^{S}]$^{3+}$**	XRD	18.47(22)		
	DFT	*17.09*		
**[1^{O}]$^{3+}$**	XRD	21.84(186)		
	DFT	*25.17*		
**[1^{NMe}]$^{3+}$**	XRD	18.31(210)		
	DFT	*18.79*		
**[1^{CH2}]$^{3+}$**	XRD	29.54(20)		
	DFT	*26.07*		
		δ^1 (Py^3, Py^5)	δ^2 (Py^4, Py^6)	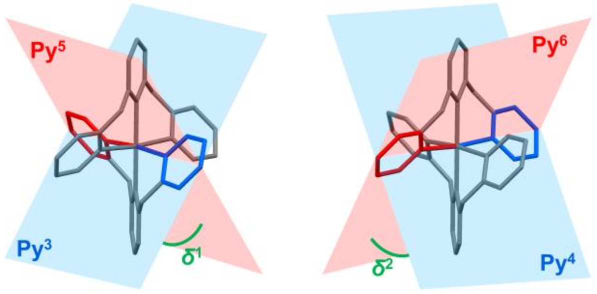
**[1^{S}]$^{3+}$**	XRD	81.68(18)	86.26(17)	
	DFT	*82.03*	*81.85*	
**[1^{O}]$^{3+}$**	XRD	73.51(122)	69.75(235)	
	DFT	*68.41*	*68.56*	
**[1^{NMe}]$^{3+}$**	XRD	79.06(75)	80.63(91)	
	DFT	*78.79*	*78.68*	
**[1^{CH2}]$^{3+}$**	XRD	72.28(32)	71.23(21)	
	DFT	*70.38*	*69.89*	
		$\measuredangle$(Py^3, CrN_4)	$\measuredangle$(Py^4, CrN_4)	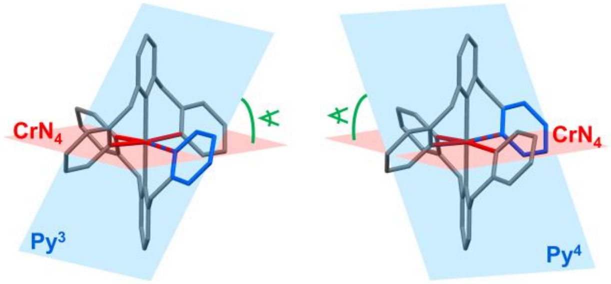
**[1^{S}]$^{3+}$**	XRD	49.48(18)	47.39(21)	
	DFT	*48.99*	*49.07*	
**[1^{O}]$^{3+}$**	XRD	55.42(82)	56.31(180)	
	DFT	*55.79*	*55.73*	
**[1^{NMe}]$^{3+}$**	XRD	49.73(115)	46.82(73)	
	DFT	*50.69*	*50.60*	
**[1^{CH2}]$^{3+}$**	XRD	56.64(16)	53.96(14)	
	DFT	*55.16*	*54.87*	
		$\measuredangle$(Py^5, CrN_4)	$\measuredangle$(Py^6, CrN_4)	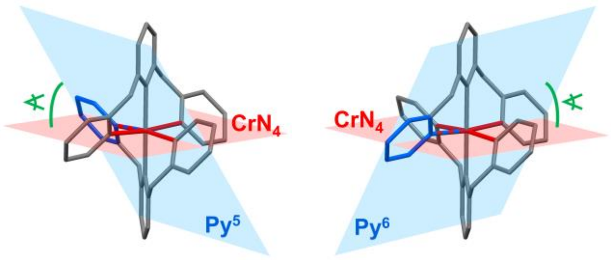
**[1^{S}]$^{3+}$**	XRD	49.13(16)	46.60(17)	
	DFT	*48.98*	*49.07*	
**[1^{O}]$^{3+}$**	XRD	51.07(126)	54.03(218)	
	DFT	*55.80*	*55.72*	
**[1^{NMe}]$^{3+}$**	XRD	51.27(104)	52.58(85)	
	DFT	*50.53*	*50.72*	
**[1^{CH2}]$^{3+}$**	XRD	52.28(29)	55.39(20)	
	DFT	*54.47*	*55.24*	

Table S3 TD-DFT calculated oscillator strengths and difference electron densities of the ten lowest energy spin-allowed transitions of **[1^{O}]$^{3+}$**.

#	λ / nm	oscillator strength	character	from	to	difference electron density (purple: density loss, orange: density gain)
1	419.0	0.0000032	MC (61%) + MLCT (24%)	t_{2g} (d_{xy})	e_g ($d_{x^2-y^2}$)	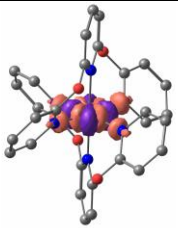
2	414.3	0.0002689	MC (62%) + MLCT (24%)	t_{2g} (d_{xz}+d_{yz})’	e_g (d_{z^2})	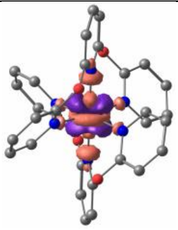
3	398.4	0.0009169	MC (62%) + MLCT (25%)	t_{2g} (d_{xz}+d_{yz})’ + t_{2g} (d_{xz}+d_{yz})’’	e_g (d_{z^2})	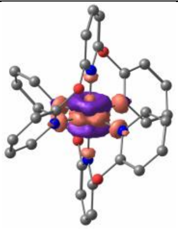
4	366.2	0.0131411	LMCT (48%) + LC (32%)	p (O) and π (Py_t)	t_{2g} (d_{xy}) and π (Py_t)	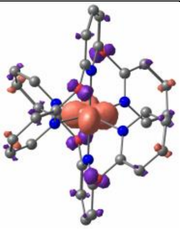
5	364.7	0.0013474	LMCT (42%) + LC (32%)	p (O) and π (Py_c)	t_{2g} (d_{xz}+d_{yz})’ and π (Py_c)	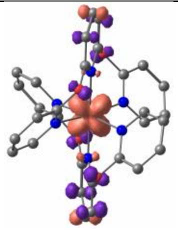
6	364.1	0.0134968	LMCT (41%) + LC (34%)	p (O) and π (Py_c)	t_{2g} (d_{xz}+d_{yz})’ and π (Py_c)	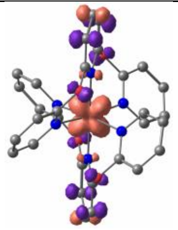
7	361.2	0.0000006	LMCT (49%) + ILCT (26%)	p (O) and π (Py_c)	t_{2g} (d_{xy}) and π (Py_t)	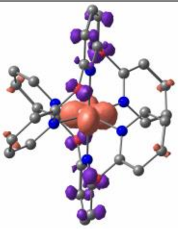

#	λ / nm	oscillator strength	character	from	to	difference electron density (purple: density loss, orange: density gain)
8	350.2	0.0000000	MC (57%) + MLCT (23%)	t_{2g} (d_{xy})	e_g (d_{z^2})	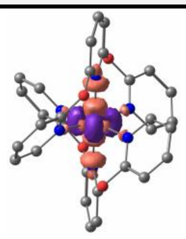
9	348.0	0.0028086	LMCT (47%) + LC (36%)	p (O)	t_{2g} (d_{xz}+d_{yz})''	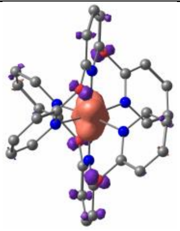
10	347.2	0.0006875	LMCT (55%) + LC (27%)	p (O)	t_{2g} (d_{xz}+d_{yz})''	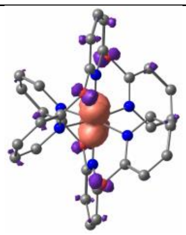

Table S4 TD-DFT calculated oscillator strengths and difference electron densities of the ten lowest energy spin-allowed transitions of **$[1^S]^{3+}$**.

#	λ / nm	oscillator strength	character	from	to	difference electron density (purple: density loss, orange: density gain)
1	477.0	0.0000202	MC (57%) + MLCT (22%)	t_{2g} (d_{xz}+d_{yz})'	e_g (d_{z^2})	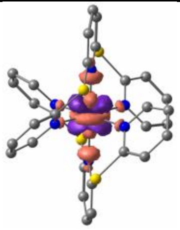
2	448.9	0.0001916	MC (59%) + MLCT (23%)	t_{2g} (d_{xz}+d_{yz})' + t_{2g} (d_{xz}+d_{yz})''	e_g (d_{z^2})	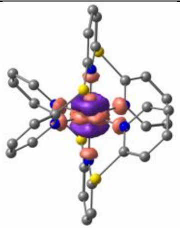
3	447.0	0.0000868	MC (56%) + MLCT (23%)	t_{2g} (d_{xy})	e_g ($d_{x^2-y^2}$)	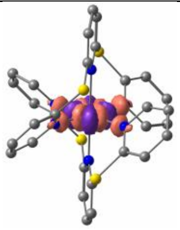

#	λ / nm	oscillator strength	character	from	to	difference electron density (purple: density loss, orange: density gain)
4	446.2	0.0000026	LMCT (53%)<br>+ ILCT (32%)	p (S)<br>and<br>π (Py_c)	t_{2g} (d_{xy})	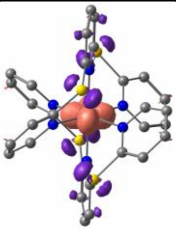
5	445.6	0.0228827	LMCT (44%)<br>+ ILCT (36%)	p (S)<br>and<br>π (Py_c)	t_{2g} (d_{xz}+d_{yz})'<br>and<br>π (Py_c)	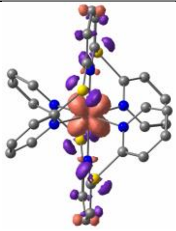
6	429.8	0.0001514	LMCT (41%)<br>+ ILCT (37%)	p (S)<br>and<br>π (Py_c)	t_{2g} (d_{xz}+d_{yz})'<br>+<br>t_{2g} (d_{xz}+d_{yz})''<br>and<br>π (Py_c)	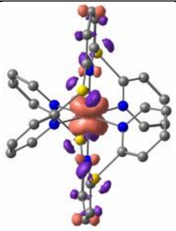
7	426.1	0.0072654	LMCT (51%)<br>+ ILCT (29%)	p (S)	t_{2g} (d_{xy})	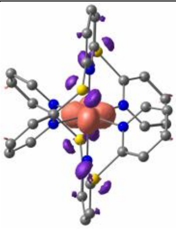
8	416.6	0.0023140	LMCT (60%)<br>+ ILCT (26%)	p (S)<br>and<br>π (Py_c)	t_{2g} (d_{xz}+d_{yz})''	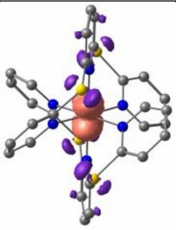
9	409.4	0.0000000	LMCT (55%)<br>+ ILCT (19%)	p (S)	e_g (d_{z^2})<br>and<br>e_g ($d_{x^2-y^2}$)	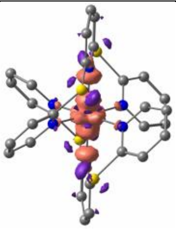
10	402.6	0.0004712	LMCT (52%)<br>+ ILCT (28%)	p (S)	t_{2g} (d_{xz}+d_{yz})''	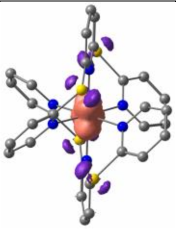

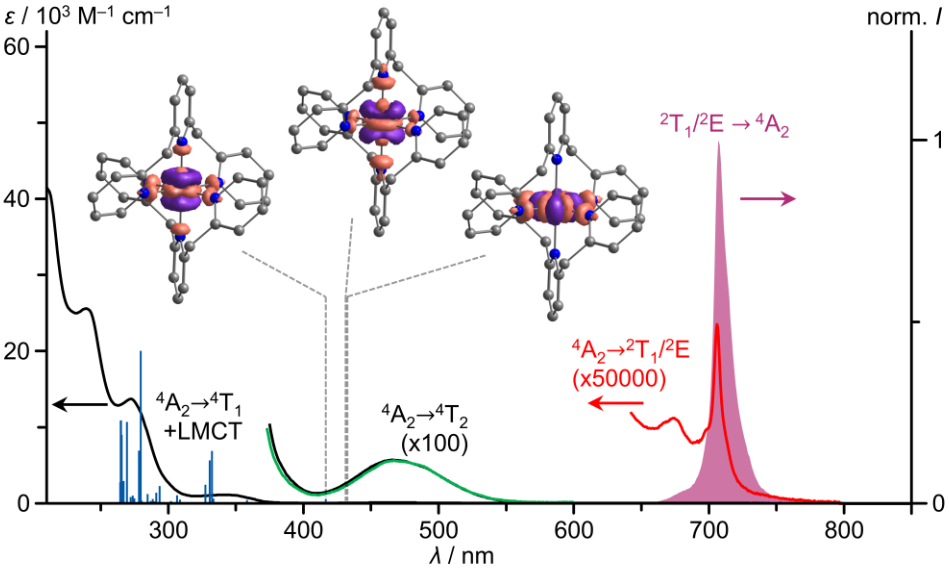

Fig. S39 UV/VIS/NIR absorption spectrum (black, red), excitation spectrum (λ_{em} = 708 nm, green) and emission spectrum (λ_{exc} = 462 nm, purple) of **[1^{CH2}][OTf]$_3$** in deaerated acetonitrile at room temperature, TD-DFT calculated oscillator strengths (blue) and difference electron densities of three low energy transitions of ^{4}MC character of **[1^{CH2}]$^{3+}$**.[43] The regions of the spin forbidden absorption bands, the lowest energy spin allowed absorption band and the excitation spectrum are zoomed.

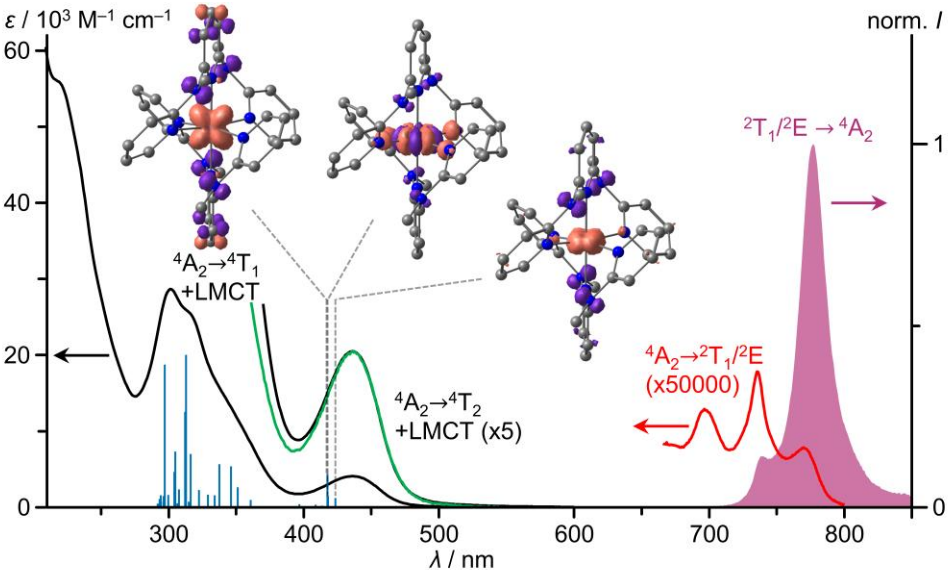

Fig. S40 UV/VIS/NIR absorption spectrum (black, red), excitation spectrum (λ_{em} = 775 nm, green) and emission spectrum (λ_{exc} = 435 nm, purple) of **[1^{NMe}][BF$_4$]$_3$** in deaerated acetonitrile at room temperature, TD-DFT calculated oscillator strengths (blue) and difference electron densities of three low energy transitions of ^{4}LMCT and ^{4}MC character of **[1^{NMe}]$^{3+}$**.[44] The regions of the spin forbidden absorption bands, the lowest energy spin allowed/LMCT absorption band and the excitation spectrum are zoomed.

Table S5 CASSCF(x,y)-SC/NEVPT2 results of the complex series **$[1^X]^{3+}$** depending on the active space with energies in cm^{-1} (bold values indicate the lowest state of each multiplicity).

Complex	(x,y)	$^2E(1)$	$^2E(2)$	$^2T_1(1)$	$^2T_1(2)$	$^2T_1(3)$	$^2T_2(1)$	$^2T_2(2)$	$^2T_2(3)$	$^4T_2(1)$	$^4T_2(2)$	$^4T_2(3)$
**$[1^O]^{3+}$**	(3,5)	16440	17239	**15924**	16849	17181	25217	25641	25674	**22000**	22486	23571
	(3,10)	16196	16991	**15659**	16580	16936	24926	25372	25376	**21614**	22068	23168
	(13,10)	16271	16912	**15760**	16515	16792	24507	25053	25094	**22267**	22638	23787
	(7,12)	15726	16299	**15265**	15915	16206	23861	24310	24334	**22664**	23047	24073
**$[1^S]^{3+}$**	(3,5)	16610	17470	**16325**	17228	17501	25045	25415	25489	**19511**	20638	20667
	(3,10)	16369	17224	**16067**	16950	17266	24786	25110	25185	**19144**	20294	20304
	(13,10)	16536	17154	**16226**	16938	17159	24564	24835	24878	**19762**	20974	21040
	(7,12)	15958	16544	**15725**	16292	16568	23835	24178	24186	**20293**	21372	21393
**$[1^{NMe}]^{3+}$**	(3,5)	16345	17155	**15712**	16897	17002	25205	25414	25779	**22626**	22648	23846
	(3,10)	16091	16902	**15454**	16625	16758	24940	25101	25479	**22233**	22243	23432
	(13,10)	16140	16749	**15460**	16519	16545	24551	24588	25074	**22700**	22818	23928
	(7,12)	15597	16180	**15051**	15928	16011	23823	23984	24361	**23216**	23262	24310
**$[1^{CH2}]^{3+}$**	(3,5)	16562	17407	**16331**	17135	17440	25270	25462	25502	**20914**	21103	22027
	(3,10)	16322	17165	**16069**	16858	17206	25007	25173	25200	**20571**	20692	21636
	(13,10)	16503	17144	**16286**	16894	17125	24814	24899	25014	**21276**	21415	22374
	(7,12)	15882	16454	**15691**	16208	16467	24006	24085	24208	**21727**	21840	22679

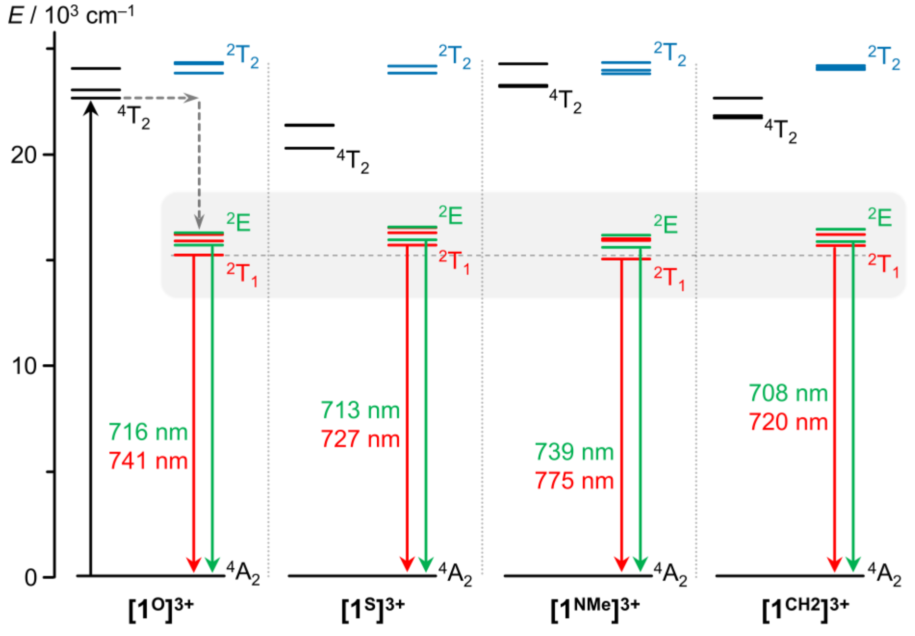

Fig. S41 CASSCF(7,12)-SC-NEVPT2 calculated energy diagram of the metal centred excited states of the **$[1^X]^{3+}$** complex series with experimental observed maxima of the main room temperature emission bands and arrows indicating excitation (black) inter-system-crossing/internal conversion/vibrational cooling (dotted) and emission (red from $^2T_1$, green from ^2E).

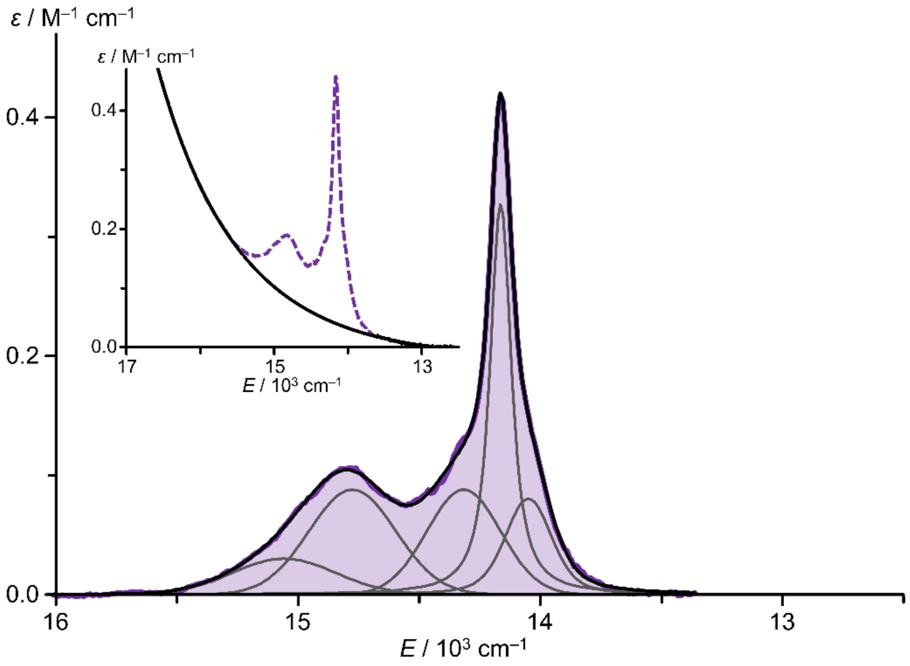

Fig. S42 Baseline corrected NIR absorption spectra of **[1^{CH2}][OTf]$_3$** (purple) in acetonitrile with sum fit (black) consisting of five Voigt functions (grey). Inset: NIR absorption spectra of **[1^{CH2}](OTf)$_3$** (purple, dotted) with exponential fit (black) describing the tail of the spin-allowed transitions used for the baseline correction.

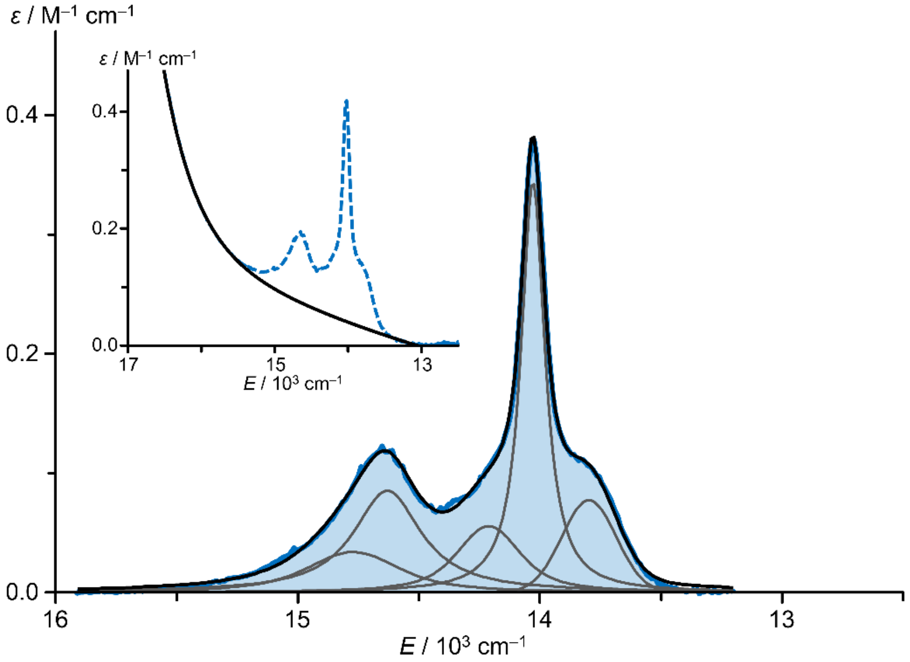

Fig. S43 Baseline corrected NIR absorption spectra of **[1^{S}][OTf]$_3$** (blue) in acetonitrile with sum fit (black) consisting of five Voigt functions (grey). Inset: NIR absorption spectra of **[1^{S}][OTf]$_3$** (blue, dotted) with exponential fit (black) describing the tail of the spin-allowed transitions used for the baseline correction.

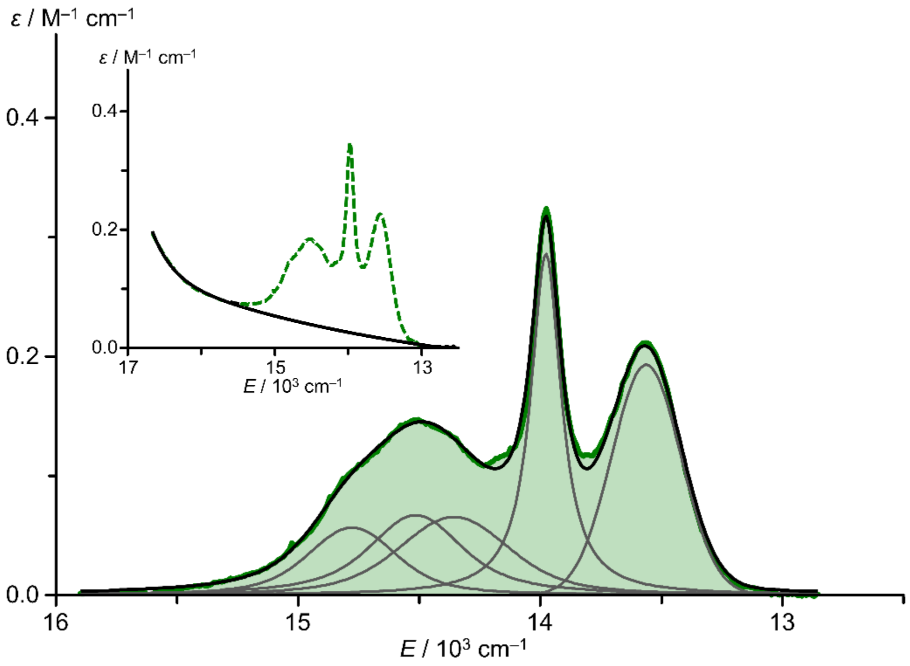

Fig. S44 Baseline corrected NIR absorption spectra of **[1^{O}][OTf]$_3$** (green) in acetonitrile with sum fit (black) consisting of five Voigt functions (grey). Inset: NIR absorption spectra of **[1^{O}][OTf]$_3$** (green, dotted) with exponential fit (black) describing the tail of the spin-allowed transitions used for the baseline correction.

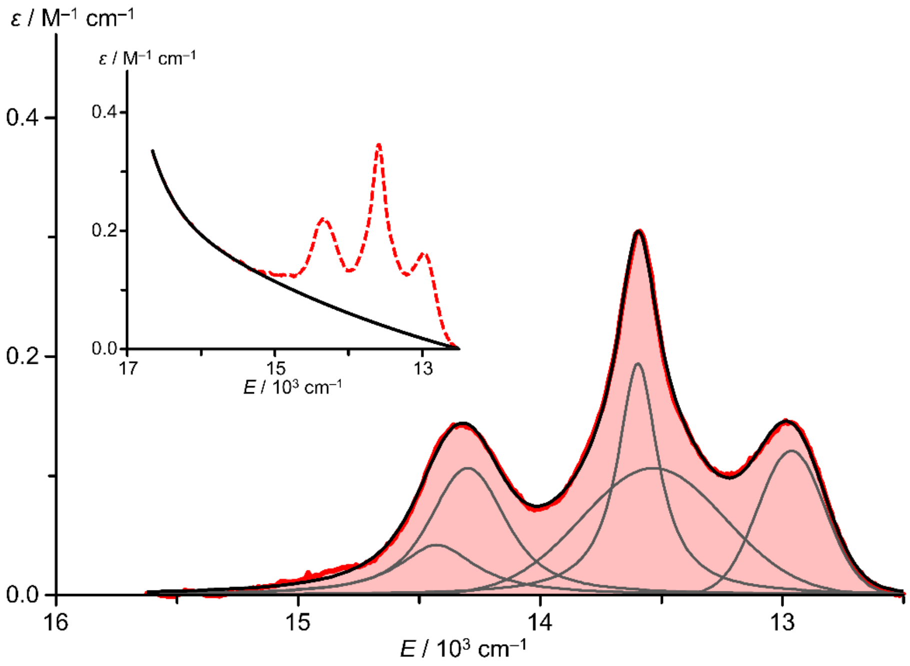

Fig. S45 Baseline corrected NIR absorption spectra of **[1^{NMe}][BF$_4$]$_3$** (red) in acetonitrile with sum fit (black) consisting of five Voigt functions (grey). Inset: NIR absorption spectra of **[1^{NMe}][BF$_4$]$_3$** (red, dotted) with exponential fit (black) describing the tail of the spin-allowed transitions used for the baseline correction.

Computational studies on the doublet energies

Table S6 CASSCF(7,12)-SC/NEVPT2 results of the complex series $[\mathbf{1}^{X}]^{3+}$ at DFT optimised geometries of the four Molecular Rubies with energies in cm^{-1} (bold values indicate the lowest state of each multiplicity).

$[\mathbf{1}^{X}]^{3+}$	Geo.	$^2E(1)$	$^2E(2)$	$^2T_1(1)$	$^2T_1(2)$	$^2T_1(3)$	$^2T_2(1)$	$^2T_2(2)$	$^2T_2(3)$	$^4T_2(1)$	$^4T_2(2)$	$^4T_2(3)$
$[\mathbf{1}^{O}]^{3+}$	$[\mathbf{1}^{O}]^{3+}$	15726	16299	**15265**	15915	16206	23861	24310	24334	**22664**	23047	24073
	$[\mathbf{1}^{S}]^{3+}$	16089	16678	**15944**	16396	16735	23926	24100	24290	**19584**	20500	20824
	$[\mathbf{1}^{NMe}]^{3+}$	15650	16215	**15131**	15892	16170	23923	24004	24336	**23139**	23235	24299
	$[\mathbf{1}^{CH2}]^{3+}$	16000	16574	**15838**	16258	16593	24129	24136	24196	**20907**	21266	21770
$[\mathbf{1}^{S}]^{3+}$	$[\mathbf{1}^{O}]^{3+}$	15378	15920	**14183**	15436	15580	23569	23951	24622	**23222**	23249	24567
	$[\mathbf{1}^{S}]^{3+}$	15958	16544	**15725**	16292	16568	23835	24178	24186	**20293**	21372	21393
	$[\mathbf{1}^{NMe}]^{3+}$	15313	15868	**14050**	15392	15467	23558	23841	24666	**23312**	23748	24691
	$[\mathbf{1}^{CH2}]^{3+}$	15759	16325	**15433**	16064	16290	23834	23988	24064	**21506**	22016	22594
$[\mathbf{1}^{NMe}]^{3+}$	$[\mathbf{1}^{O}]^{3+}$	15669	16257	**15224**	15983	16115	23796	24172	24324	**22674**	23213	24130
	$[\mathbf{1}^{S}]^{3+}$	16040	16622	**15882**	16374	16677	23892	24149	24253	**20119**	20981	21047
	$[\mathbf{1}^{NMe}]^{3+}$	15597	16180	**15051**	15928	16011	23823	23984	24361	**23216**	23262	24310
	$[\mathbf{1}^{CH2}]^{3+}$	15958	16535	**15796**	16293	16555	24041	24132	24145	**21141**	21344	21982
$[\mathbf{1}^{CH2}]^{3+}$	$[\mathbf{1}^{O}]^{3+}$	15599	16159	**15078**	15832	16035	23746	24159	24327	**23045**	23916	24780
	$[\mathbf{1}^{S}]^{3+}$	16006	16585	**15868**	16376	16633	23868	24185	24256	**20689**	21169	21534
	$[\mathbf{1}^{NMe}]^{3+}$	15522	16088	**14933**	15799	15936	23780	23927	24342	**23529**	24048	24975
	$[\mathbf{1}^{CH2}]^{3+}$	15882	16454	**15691**	16208	16467	24006	24085	24208	**21727**	21840	22679

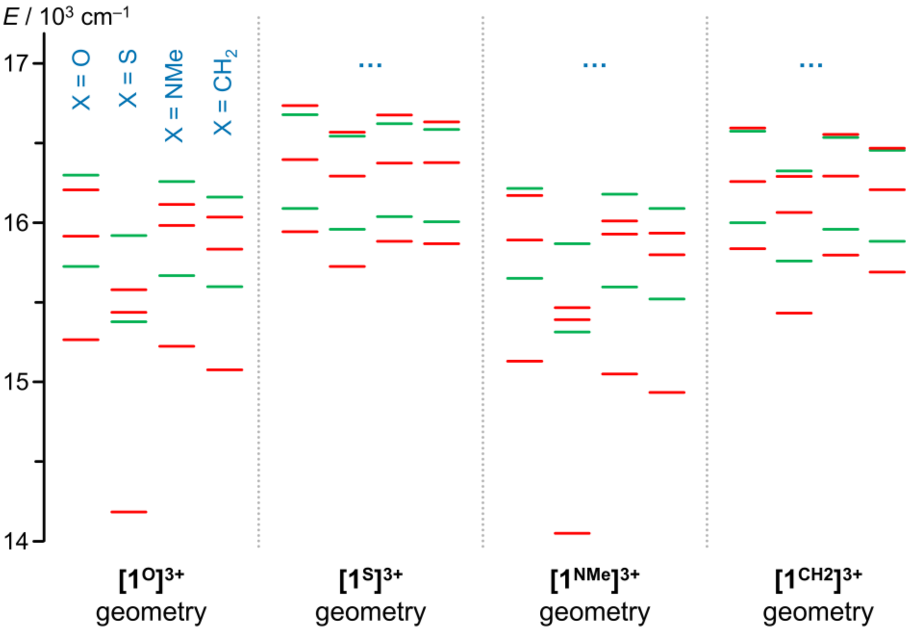

Fig. S46 CASSCF(7,12)-SC-NEVPT2 calculated $^2T_1$ (red) and ^2E energies (green) of $[\mathbf{1}^{X}]^{3+}$ (X = O, S, NMe, CH_2) at DFT-optimised geometries of $[\mathbf{1}^{X}]^{3+}$ (X = O, S, NMe, CH_2) – X substitution at fixed geometry.

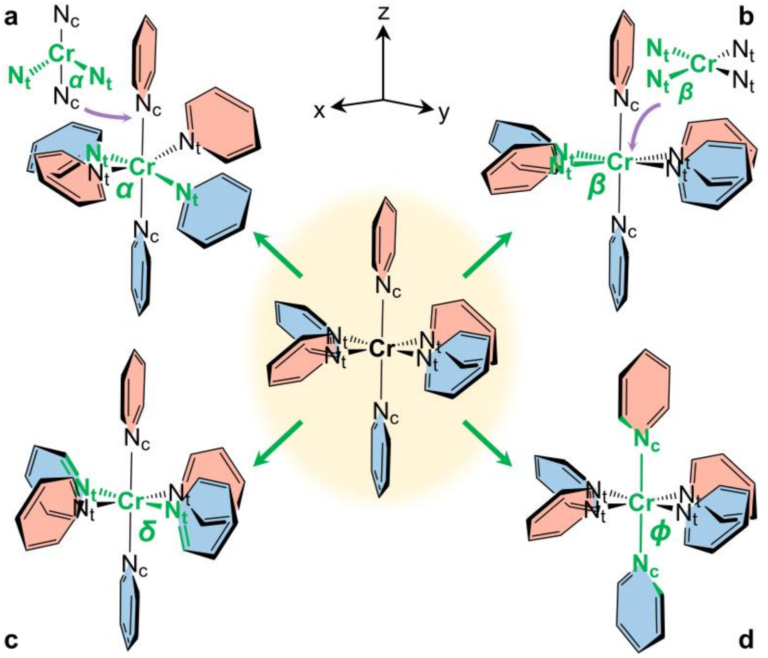

Fig. S47 Parameters α, β, δ and ϕ used for characterising the structure of the Molecular Rubies **$[1^X]^{3+}$** (X = S, O, NMe, CH_2) illustrated on the reference $[Cr(py)_6]^{3+}$. The structures demonstrate, which structural modifications were performed on the reference system $[Cr(py)_6]^{3+}$ to investigate their influence on the doublet energies: Compression of the angle α to yield N_t distortion within the xz/yz plane (a); compression of the angle β to yield N_t distortion within the xy plane (b); torsion δ of the terminal pyridine (c); torsion ϕ of the central pyridines (d).

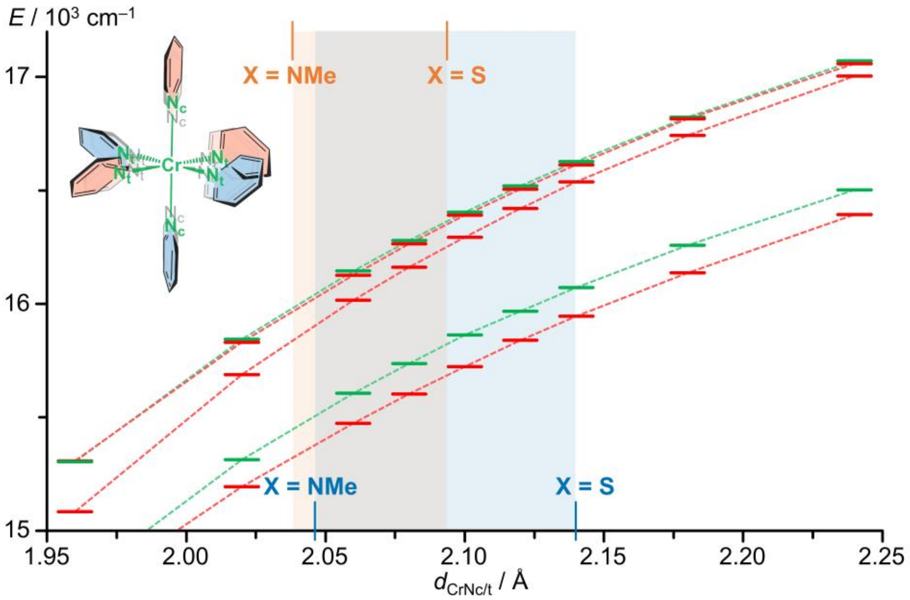

Fig. S48 CASSCF(7,12)-SC-NEVPT2 results of the model $[Cr(py)_6]^{3+}$ showing the dependence of the $^2T_1$ (red) and ^2E energies (green) on the Cr–$N_{c/t}$ elongation in local O_h symmetry including structures illustrating the geometry modification. The corresponding parameter for **$[1^X]^{3+}$** falls within the blue and orange shaded regions according to CASSCF(7,12)-SC-NEVPT2 calculations and the crystal structures.

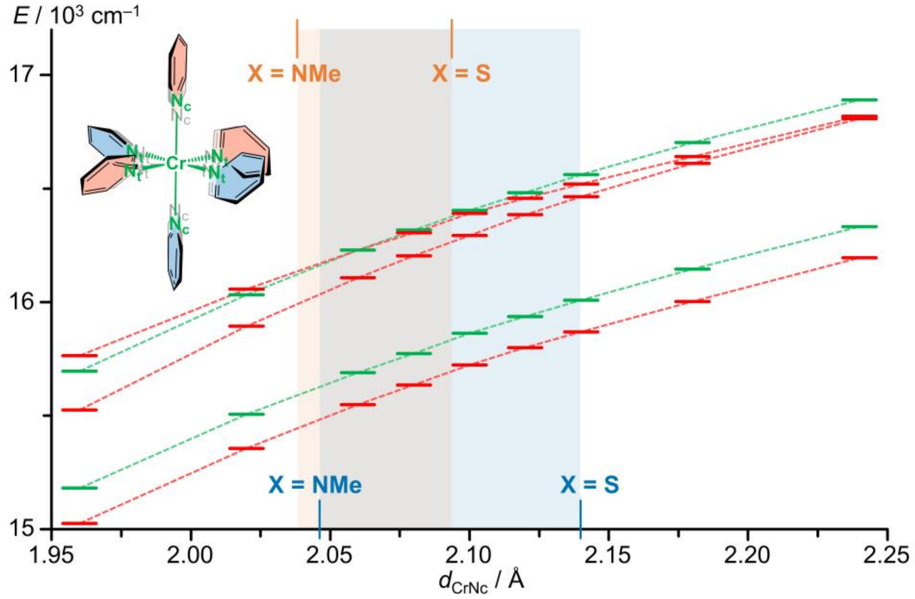

Fig. S49 CASSCF(7,12)-SC-NEVPT2 results of the model $[Cr(py)_6]^{3+}$ showing the dependence of the $^2T_1$ (red) and ^2E energies (green) on the Cr–$N_{c/t}$ elongation in local D_{4h} symmetry including structures illustrating the geometry modification. The corresponding parameter for **$[1^X]^{3+}$** falls within the blue and orange shaded regions according to CASSCF(7,12)-SC-NEVPT2 calculations and the crystal structures.

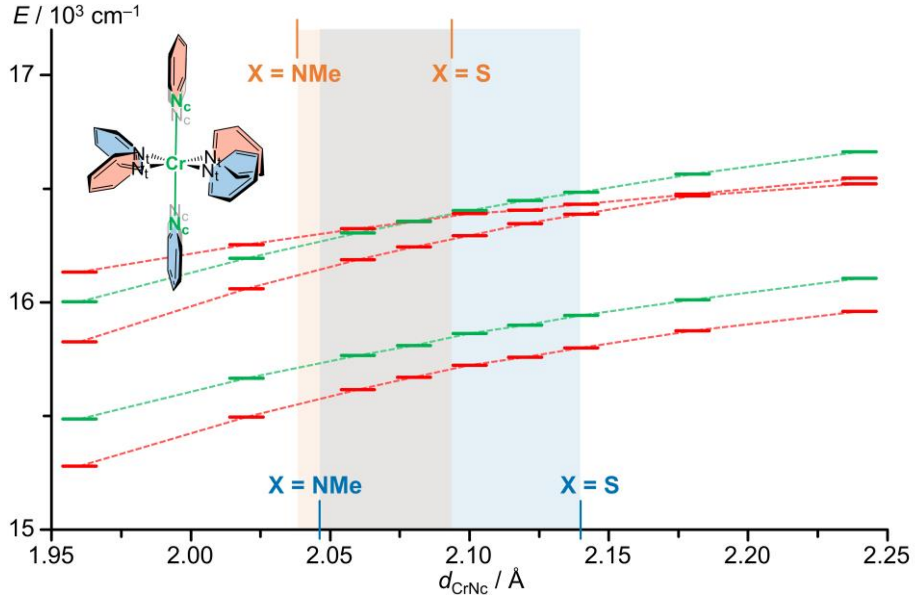

Fig. S50 CASSCF(7,12)-SC-NEVPT2 results of the model $[Cr(py)_6]^{3+}$ showing the dependence of the $^2T_1$ (red) and ^2E energies (green) on the Cr–N_c elongation including structures illustrating the geometry modification. The corresponding parameter for **$[1^X]^{3+}$** falls within the blue and orange shaded regions according to CASSCF(7,12)-SC-NEVPT2 calculations and the crystal structures.

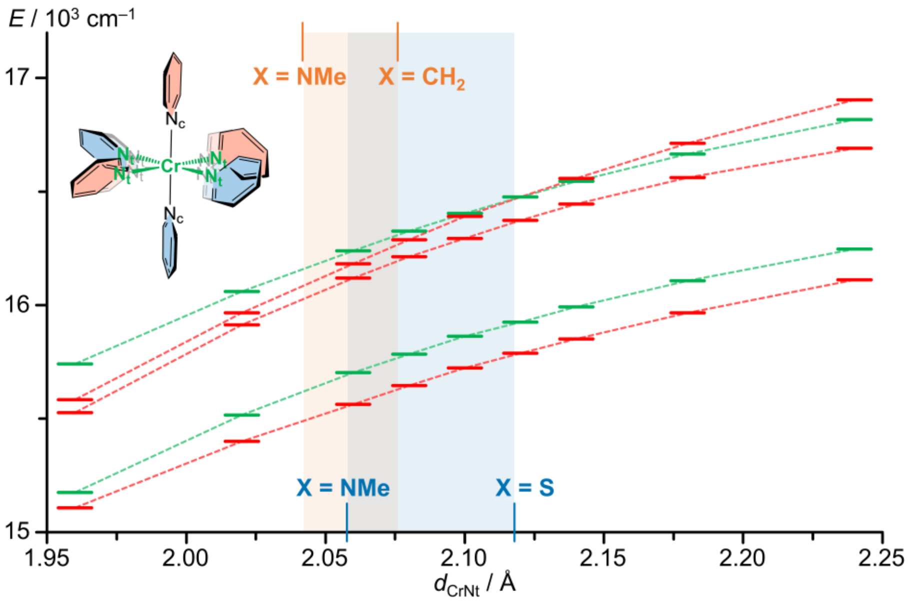

Fig. S51 CASSCF(7,12)-SC-NEVPT2 results of the model $[Cr(py)_6]^{3+}$ showing the dependence of the $^2T_1$ (red) and ^2E energies (green) on the Cr–N_t elongation including structures illustrating the geometry modification. The corresponding parameter for $\mathbf{[1^X]^{3+}}$ falls within the blue and orange shaded regions according to CASSCF(7,12)-SC-NEVPT2 calculations and the crystal structures.

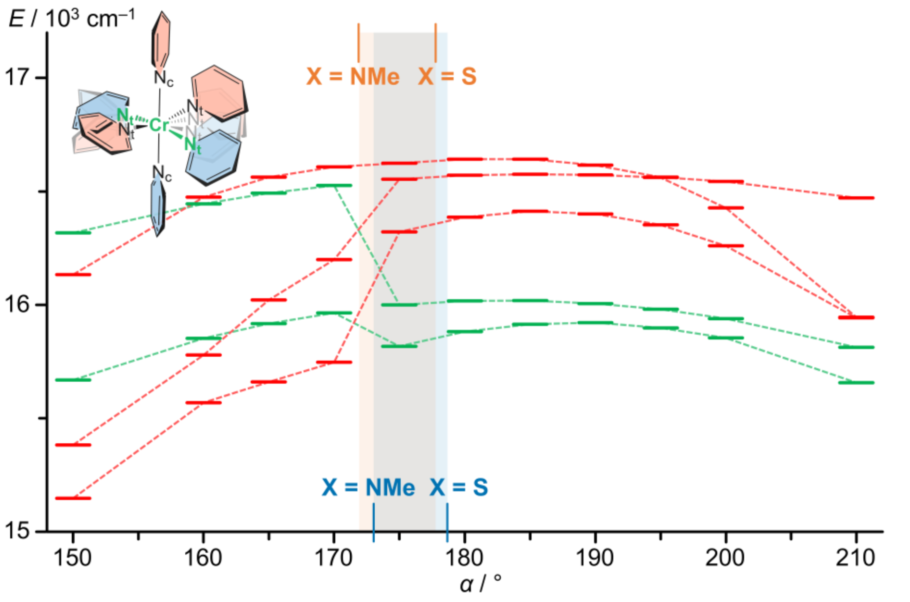

Fig. S52 CASSCF(7,12)-SC-NEVPT2 results of the model $[Cr(py)_6]^{3+}$ showing the dependence of the $^2T_1$ (red) and ^2E energies (green) on the N_t distortion within the xz/yz plane including structures illustrating the geometry modification. The corresponding parameter for $\mathbf{[1^X]^{3+}}$ falls within the blue and orange shaded regions according to CASSCF(7,12)-SC-NEVPT2 calculations and the crystal structures.

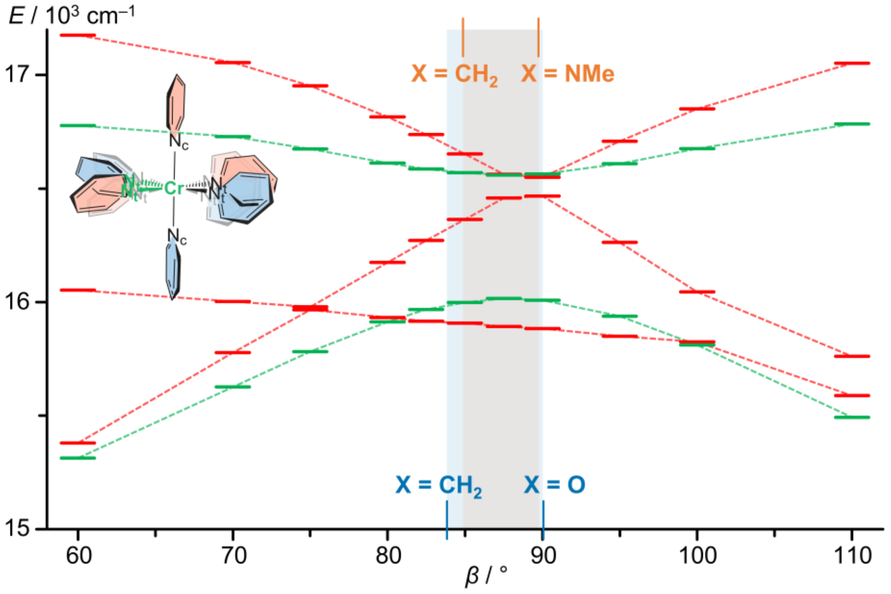

Fig. S53 CASSCF(7,12)-SC-NEVPT2 results of the model $[Cr(py)_6]^{3+}$ showing the dependence of the $^2T_1$ (red) and ^2E energies (green) on the N_t distortion within the xy plane including structures illustrating the geometry modification. The corresponding parameter for $\mathbf{[1^X]^{3+}}$ falls within the blue and orange shaded regions according to CASSCF(7,12)-SC-NEVPT2 calculations and the crystal structures.

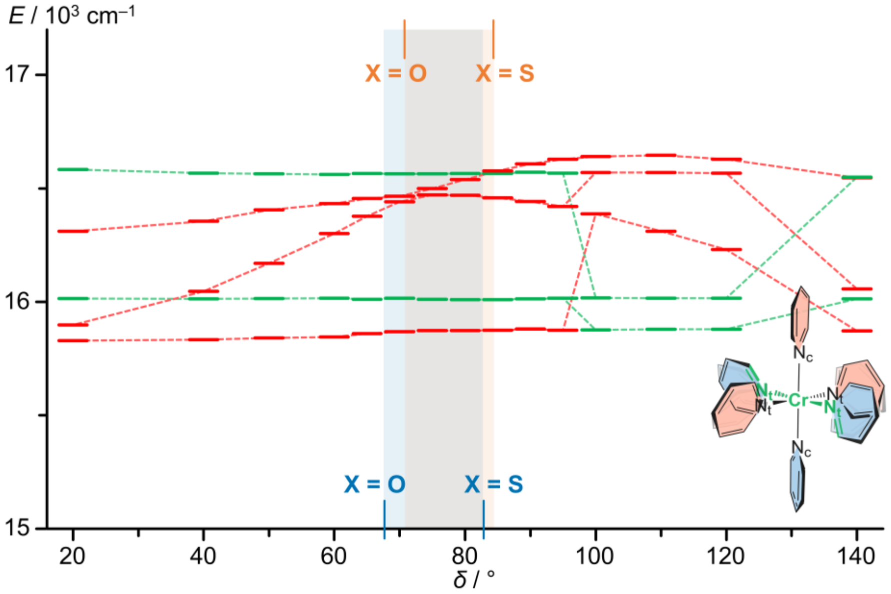

Fig. S54 CASSCF(7,12)-SC-NEVPT2 results of the model $[Cr(py)_6]^{3+}$ showing the dependence of the $^2T_1$ (red) and ^2E energies (green) on the torsion of the terminal pyridines ($\phi = 19°$) including structures illustrating the geometry modification. The corresponding parameter for $\mathbf{[1^X]^{3+}}$ falls within the blue and orange shaded regions according to CASSCF(7,12)-SC-NEVPT2 calculations and the crystal structures.

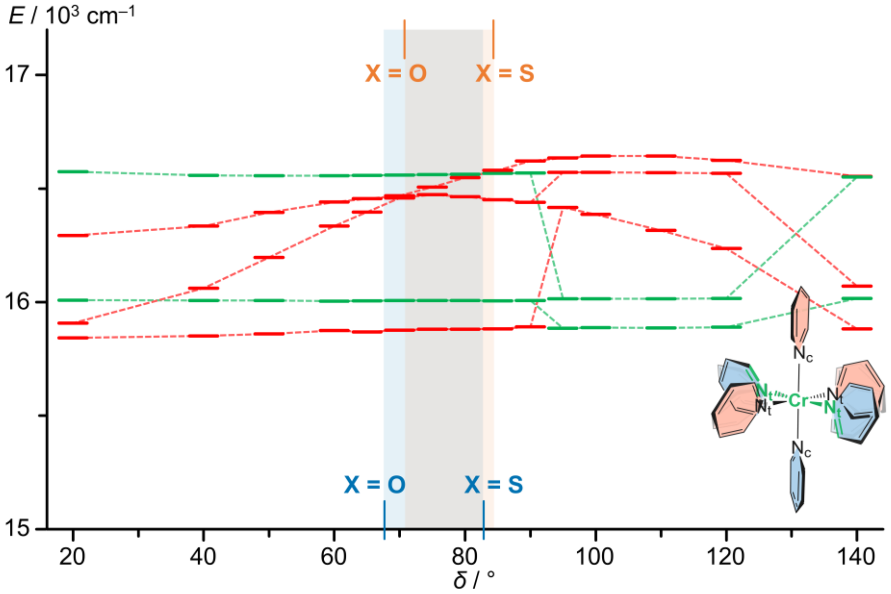

Fig. S55 CASSCF(7,12)-SC-NEVPT2 results of the model $[Cr(py)_6]^{3+}$ showing the dependence of the $^2T_1$ (red) and ^2E energies (green) on the torsion of the terminal pyridines ($\phi = 26°$) including structures illustrating the geometry modification. The corresponding parameter for **$[1^X]^{3+}$** falls within the blue and orange shaded regions according to CASSCF(7,12)-SC-NEVPT2 calculations and the crystal structures.

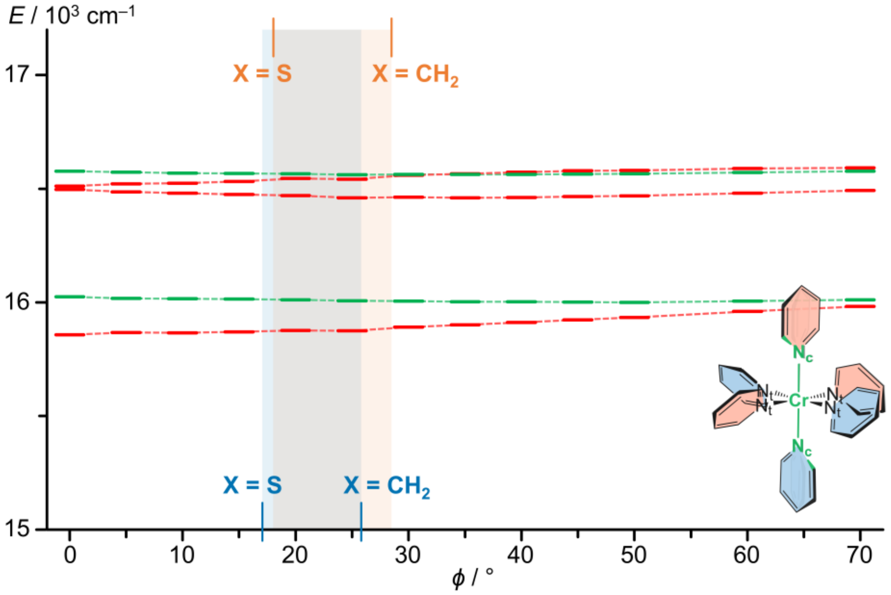

Fig. S56 CASSCF(7,12)-SC-NEVPT2 results of the model $[Cr(py)_6]^{3+}$ showing the dependence of the $^2T_1$ (red) and ^2E energies (green) on the torsion of the central pyridines ($\delta = 80°$) including structures illustrating the geometry modification. The corresponding parameter for **$[1^X]^{3+}$** falls within the blue and orange shaded regions according to CASSCF(7,12)-SC-NEVPT2 calculations and the crystal structures.

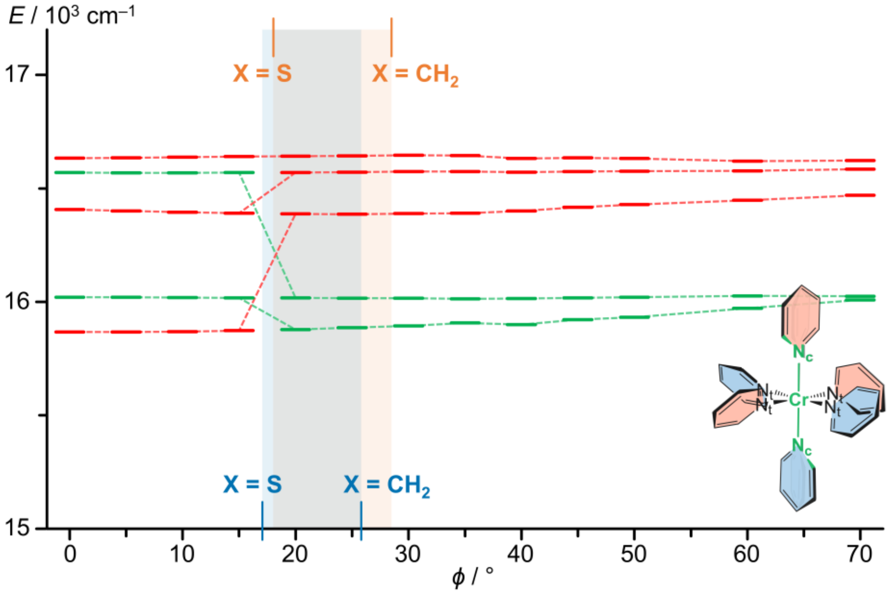

Fig. S57 CASSCF(7,12)-SC-NEVPT2 results of the model $[Cr(py)_6]^{3+}$ showing the dependence of the $^2T_1$ (red) and ^2E energies (green) on the torsion of the central pyridines (δ = 100°) including structure illustrating the geometry modification. The corresponding parameter for **[1^X]$^{3+}$** falls within the blue and orange shaded regions according to CASSCF(7,12)-SC-NEVPT2 calculations and the crystal structures.

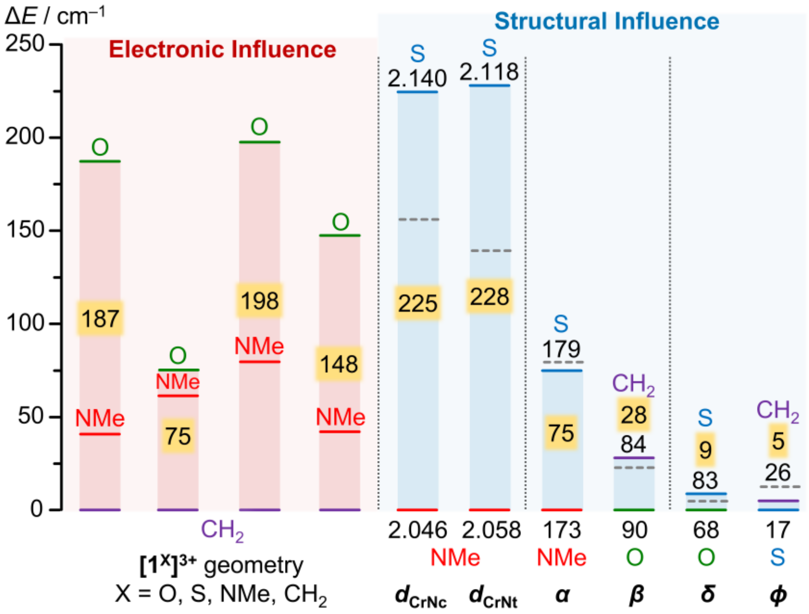

Fig. S58 Electronic and structural impact on the relative CASSCF(7,12)-SC-NEVPT2 energies of the lowest doublet states. The bridging groups X are indicated at the top and bottom of each column define the constitution at minimal and maximal energy and are described by bond lengths (in Å) and angles (in °). The maximum energy shifts ΔE highlighted in yellow are given in cm^{-1}. Left: **[1^X]$^{3+}$** (X = O: green; X = NMe: red; X = CH_2: purple). Right: $[Cr(py)_6]^{3+}$ with structural parameters taken from of the geometries of **[1^X]$^{3+}$** with X = O, S, NMe, CH_2 (green, blue, red, purple).

Computational studies on the quartet states

Table S7 Bond lengths of TD-DFT optimised geometries of quartet states of the complex series **$[1^X]^{3+}$** (bold values indicate largest bond elongations) with single point energy differences between the excited quartet states ^{4}ES and the lowest optimised doublet state ^{2}E. Distances are given in Å and energies in eV. The colour code corresponds to the different tridentate ligands.

character	Cr–N5 (N_t)	Cr–N2 (N_c)	Cr–N4 (N_t)	Cr–N7 (N_t)	Cr–N3 (N_c)	Cr–N6 (N_t)	ΔE (^{4}ES–^{2}E)
**$[1^O]^{3+}$**							
$^4A_2$	2.071	2.051	2.071	2.071	2.051	2.071	
$^4T_2(1)$	2.049	**2.107**	2.049	2.095	**2.493**	2.094	1.28
$^4T_2(2)$	2.076	**2.239**	2.076	2.077	**2.241**	2.077	1.39
**$[1^S]^{3+}$**							
$^4A_2$	2.118	2.140	2.118	2.118	2.140	2.118	
$^4T_2(1)$	2.106	**2.421**	2.109	2.073	**2.214**	2.075	1.17
$^4T_2(2)$	2.106	**2.364**	2.105	2.078	**2.238**	2.080	1.17
$^4T_2(3)$	2.086	**2.265**	2.086	2.100	**2.311**	2.100	1.17
$^4T_2(4)$	2.095	**2.290**	2.095	2.091	**2.289**	2.091	1.17
^{4}LMCT(1)	2.123	**2.356**	2.123	2.149	**2.462**	2.149	1.20
^{4}LMCT(2)	**2.405**	2.200	**2.403**	2.132	2.115	2.134	1.16
**$[1^{NMe}]^{3+}$**							
$^4A_2$	2.058	2.046	2.058	2.058	2.046	2.058	
$^4T_2$	2.036	2.061	2.036	2.138	**2.316**	2.138	1.30
^{4}LMCT(1)	2.043	2.059	2.044	2.052	2.045	2.052	1.76
^{4}LMCT(2)	**2.310**	2.087	**2.301**	2.152	2.046	2.112	1.76
**$[1^{CH2}]^{3+}$**							
$^4A_2$	2.113	2.112	2.111	2.110	2.113	2.113	
$^4T_2$	2.068	**2.161**	2.068	2.121	**2.546**	2.121	1.54

fs-Transient absorption spectroscopy

In order to obtain information on the ultrafast processes after light excitation, we measured femtosecond TA spectra of **[1^O]$^{3+}$** and **[1^S]$^{3+}$**. As the dd absorptions of **[1^O]$^{3+}$** are too weak for obtaining meaningful TA data, TA spectra of **[1^O]$^{3+}$** were recorded in CH_3CN with ^{4}LMCT excitation at 343 nm (Fig. S59–S61). Global analysis with a consecutive kinetic model yields two time constants. The initially observed broad excited state absorption (ESA) in the red to NIR spectral region decays with a time constant of τ_1 = 1.0 ps to a spectrally similar component with a lifetime of τ_2 = 47 ps. The spectral similarity of the two components decaying with τ_1 and τ_2 to the final long-lived state indicates that they belong to similar electronic states, possibly LMCT states. The ultrafast process with the time constant τ_1 likely corresponds to internal conversion (IC) and vibrational cooling (VC) on the ^{2}LMCT manifold. Finally, a blue-shifted signal that does not decay on the timescale of the experiment (8 ns) is obtained. ISC appears to be faster the time resolution of our experiment (200 fs). Due to the higher absorption coefficient of the dd/LMCT band of **[1^S]$^{3+}$**, TA spectra of **[1^S]$^{3+}$** in water were obtained with excitation at 515 nm. The initial TA spectrum features a broad ESA in the green to red spectral region that decays to a long-lived component within τ_1 = 1.2 ps (Fig. S62–S64). A small spectral shift is associated with a time constant of τ_2 = 90 ps yielding the non-decaying component, i.e. the characteristics of the long-lived SF states. The TA spectra are qualitatively similar to those of **[1^{NMe}]$^{3+}$**. Hence, we assign the ultrafast component τ_1 to ISC from the quartet to the doublet states, which is convoluted with IC/VC within the doublet manifold. The longer component τ_2 is assigned to reorganization of the solvent cage around the doublet states. The final spectrum that does not decay on the time scale of the experiment corresponds to the emissive SF states.

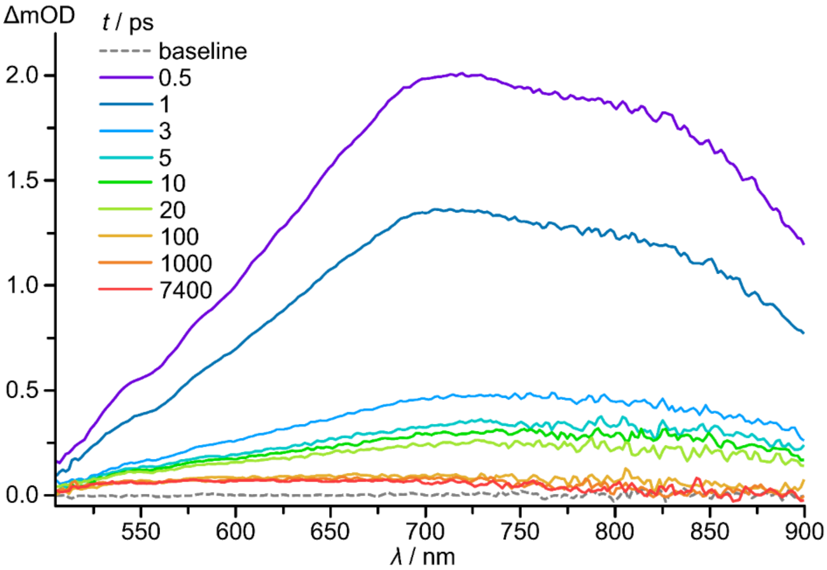

Fig. S59 Transient absorption spectra of **[1^O][OTf]$_3$** in acetonitrile after pulsed excitation at 343 nm at various time delays (fluence: 70 µJ cm^{-2}, repetition rate: 1 kHz).

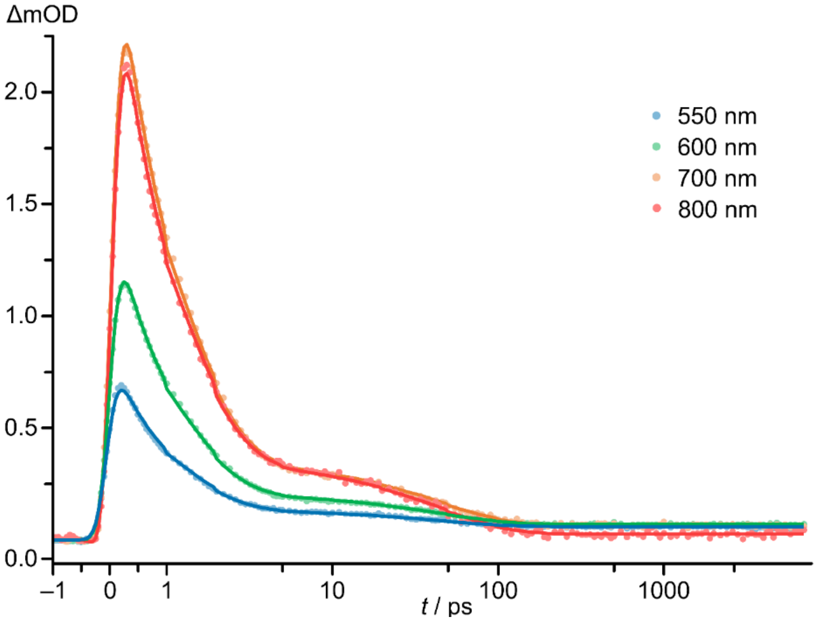

Fig. S60 Decay traces of excited state absorptions of **[1^O][OTf]₃** in acetonitrile (coloured symbols) at different wavelengths with global fit (solid lines).

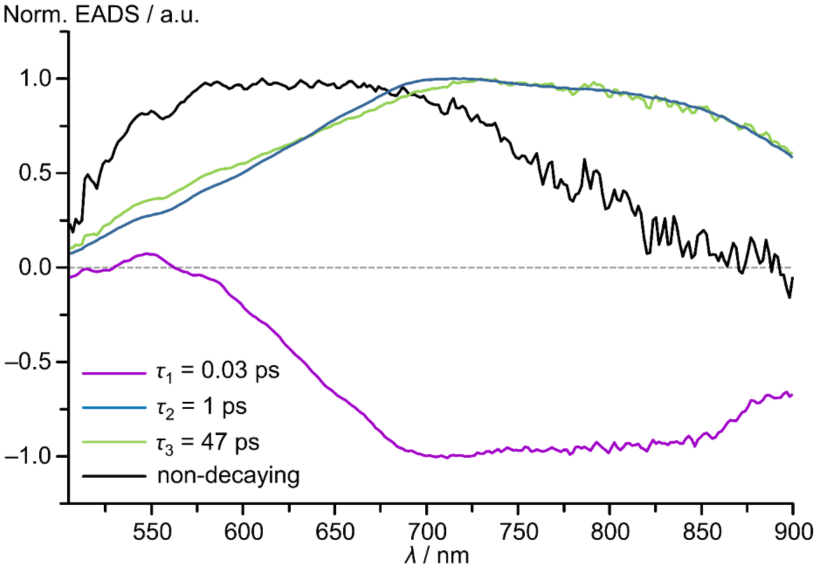

Fig. S61 Normalised evolution associated difference spectra obtained from a global analysis of transient absorption data of **[1^O][OTf]₃** (acetonitrile, λ_{exc} = 343 nm, fluence: 70 µJ cm^{-2}, repetition rate: 1 kHz).

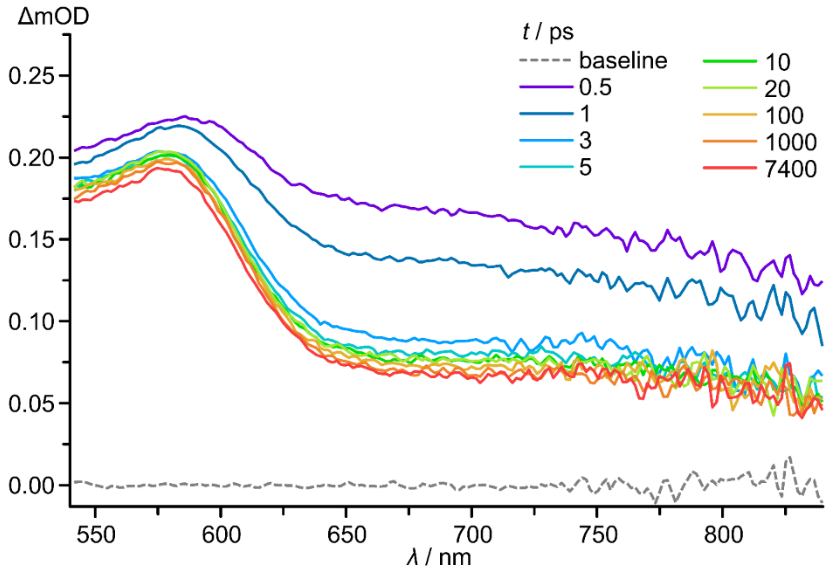

Fig. S62 Transient absorption spectra of **[1^S][OTf]$_3$** in water after pulsed excitation at 515 nm at various time delays (fluence: 3 mJ cm^{-2}, repetition rate: 1 kHz).

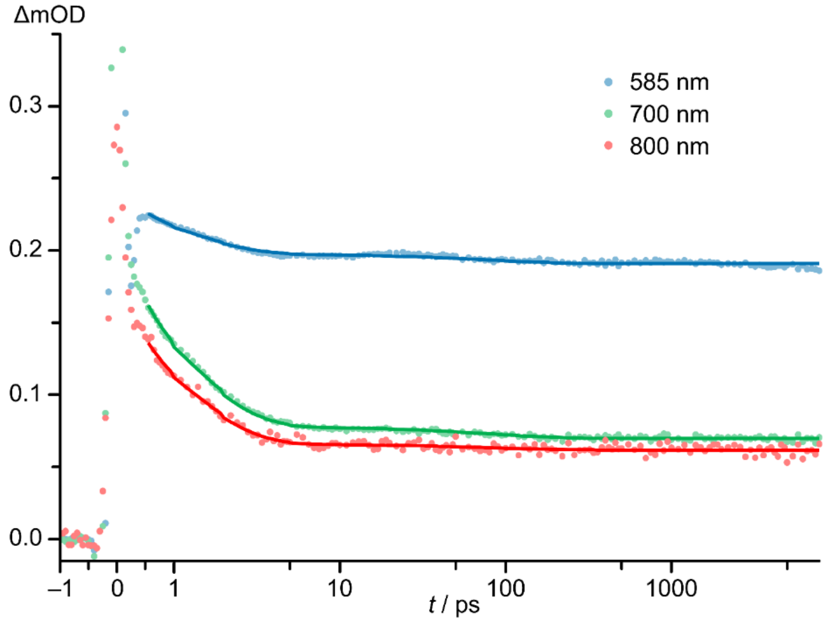

Fig. S63 Decay traces of excited state absorptions of **[1^S][OTf]$_3$** in water (coloured symbols) at different wavelengths with global fit (solid lines).

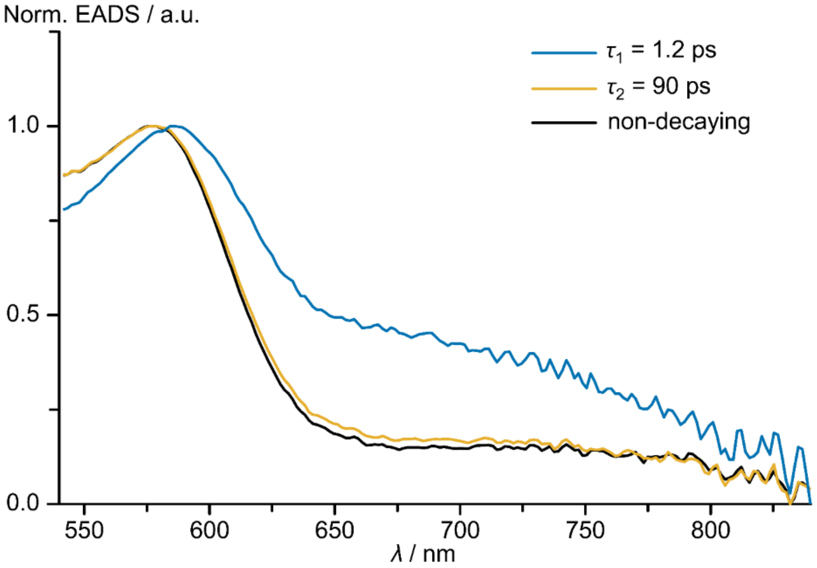

Fig. S64 Normalised evolution associated difference spectra obtained from a global analysis of transient absorption data of **[1^{S}][OTf]$_3$** (water, λ_{exc} = 515 nm, fluence: 3 mJ cm^{-2}, repetition rate: 1 kHz).

Variable-temperature emission spectroscopy

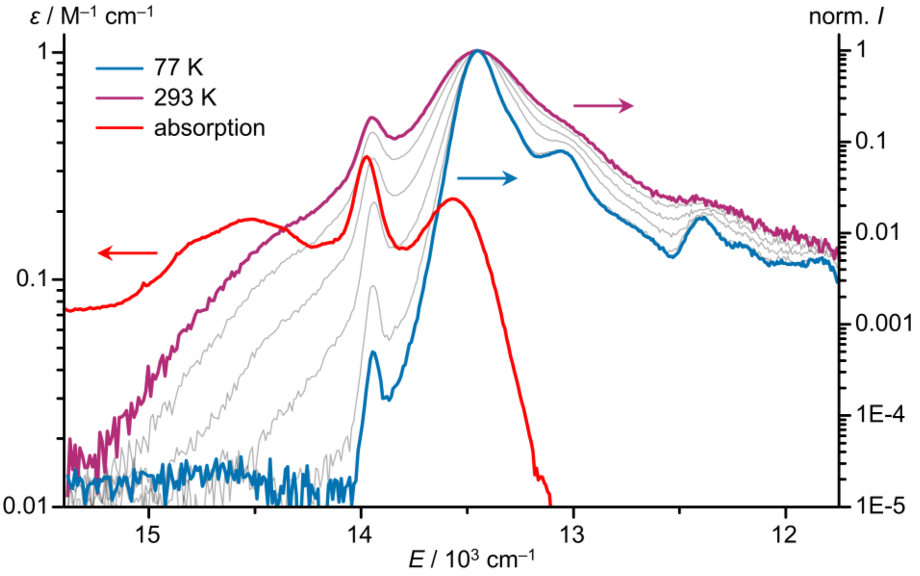

Fig. S65 Spin- and symmetry forbidden absorption band (logarithmic scale) of an acetonitrile solution (red) and temperature dependent emission spectra (λ_{exc} = 450 nm, logarithmic scale) of a (frozen) solution of **[1^{O}][OTf]$_3$** in deaerated ethanol/methanol (3:2) at 77 K (blue), 293 K (purple) and temperatures between 77 und 293 K (grey).

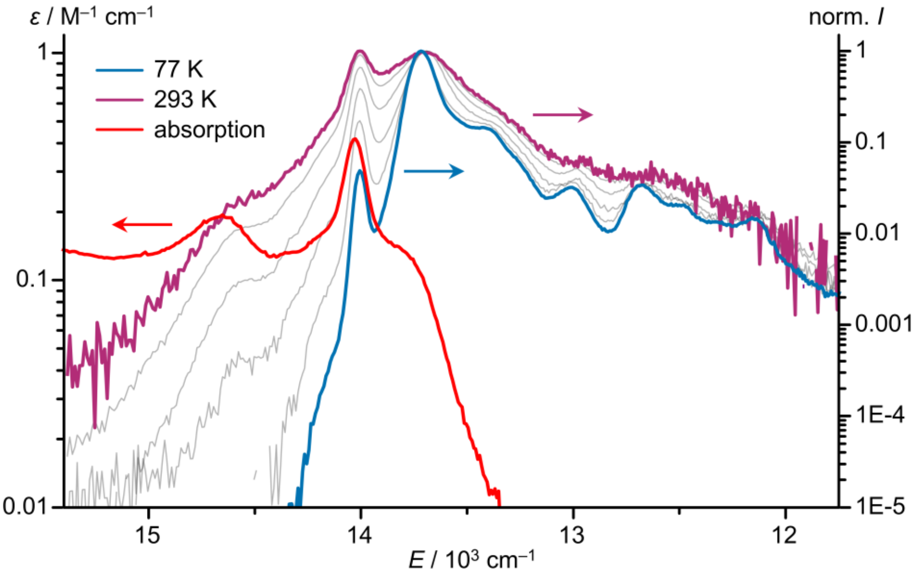

Fig. S66 Spin- and symmetry forbidden absorption band (logarithmic scale) of an acetonitrile solution (red) and temperature dependent emission spectra (λ_{exc} = 450 nm, logarithmic scale) of a (frozen) solution of **[1^{S}][OTf]$_3$** in deaerated ethanol/methanol (3:2) at 77 K (blue), 293 K (purple) and temperatures between 77 and 293 K (grey).

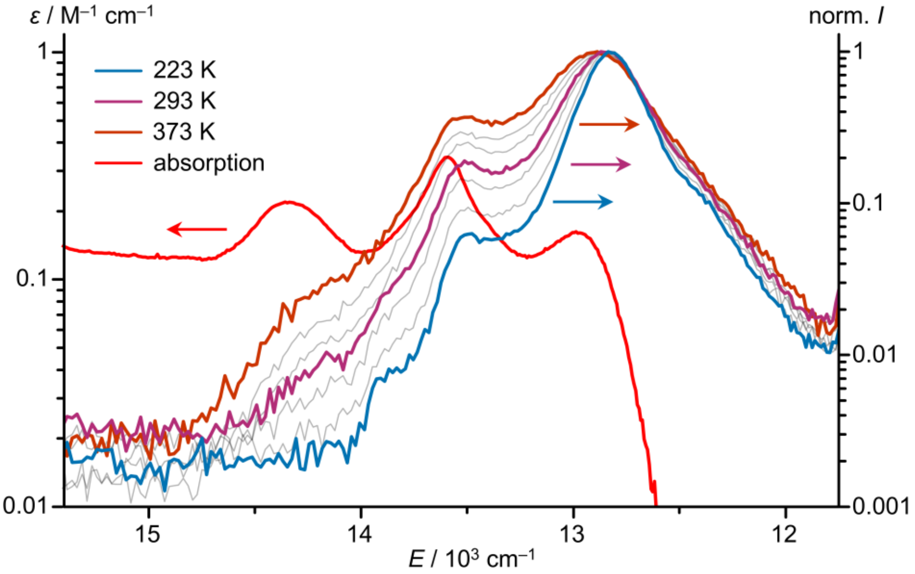

Fig. S67 Spin- and symmetry forbidden absorption band (logarithmic scale) of an acetonitrile solution (red) and temperature dependent emission spectra (λ_{exc} = 436 nm, logarithmic scale) of a solution of **[1^{NMe}][BF$_4$]$_3$** in deaerated ethylene glycol/water (2:1) at 223 K (blue), 293 K (purple), 373 K (brown) and temperatures between 223 and 373 K (grey).[44]

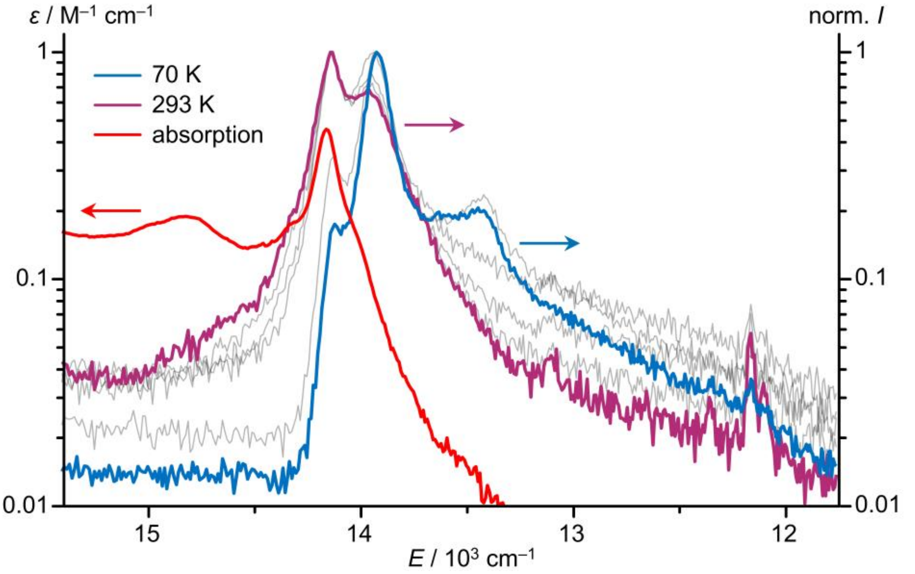

Fig. S68 Spin- and symmetry forbidden absorption band (logarithmic scale) of an acetonitrile solution (red) and temperature dependent emission spectra (λ_{exc} = 355 nm, logarithmic scale) of **[1^{CH2}][OTf]$_3$** in KBr at 70 K (blue), 293 K (purple) and temperatures between 70 and 293 K (grey).[43]

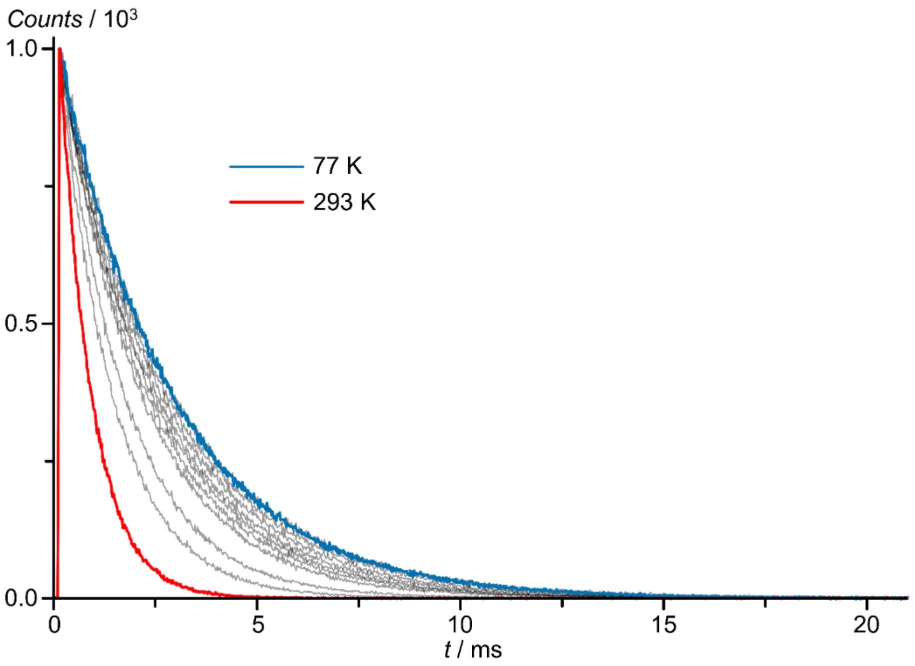

Fig. S69 Normalised temperature dependent emission decay traces recorded at 742 nm following excitation (λ_{exc} = 450 nm) of a 0.33 mM solution of **[1^{O}][OTf]$_3$** in deaerated ethanol/methanol (3:2).

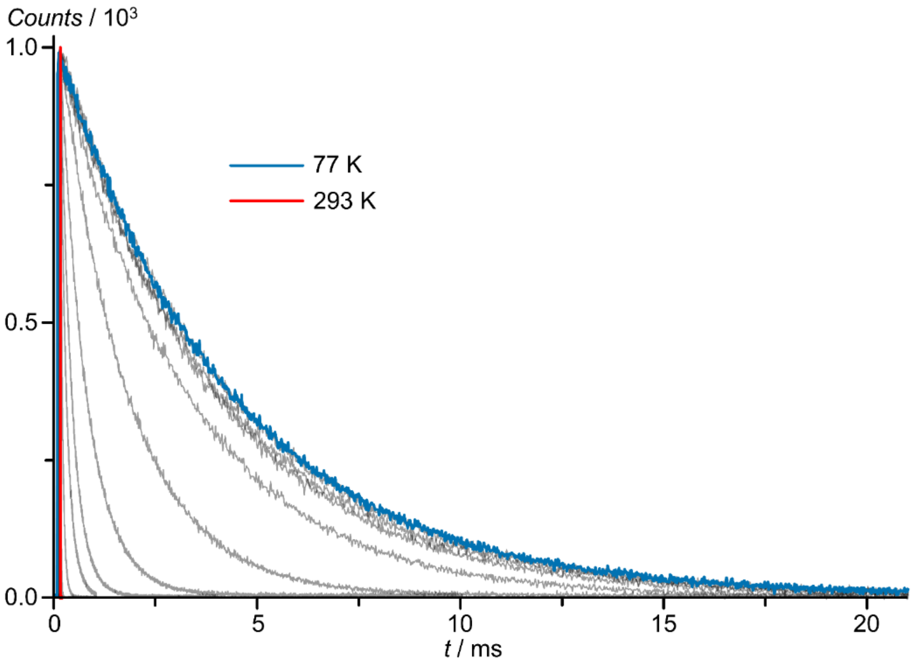

Fig. S70 Normalised temperature dependent emission decay traces recorded at 729 nm following excitation (λ_{exc} = 450 nm) of a 0.13 mM solution of **[1^{S}][OTf]$_3$** in deaerated ethanol/methanol (3:2).

Photolysis experiments

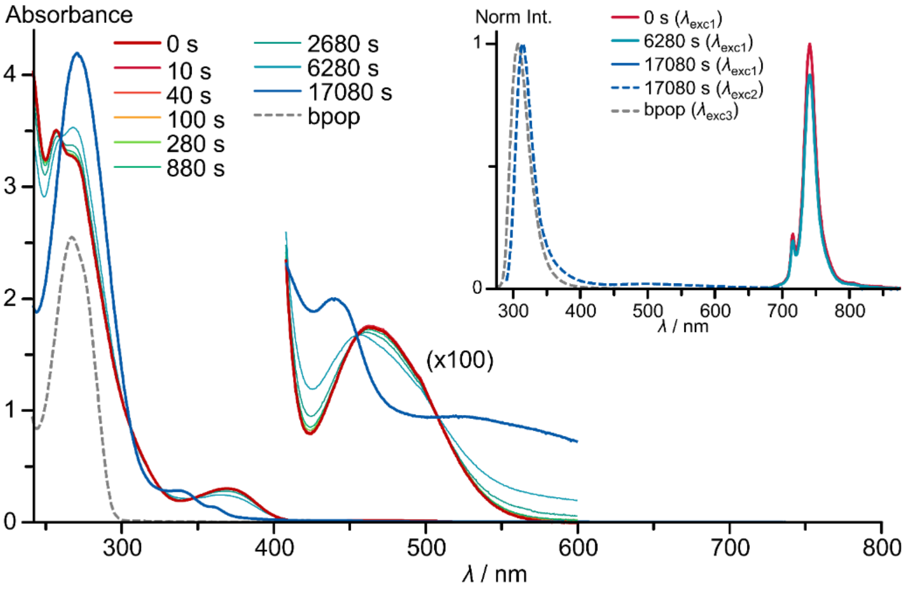

Fig. S71 UV/VIS/NIR absorption (with zoom) and normalised emission spectra of 0.205 mM **[1^{O}][OTf]$_3$** in deaerated acetonitrile after different UHP-LED irradiation time periods (λ_{exc} = 460 nm) with an output power of 1.1 W and UV/VIS/NIR absorption and normalised emission spectra of bpop in acetonitrile (λ_{exc1} = 462 nm, λ_{exc2} = 270 nm, λ_{exc3} = 267 nm).

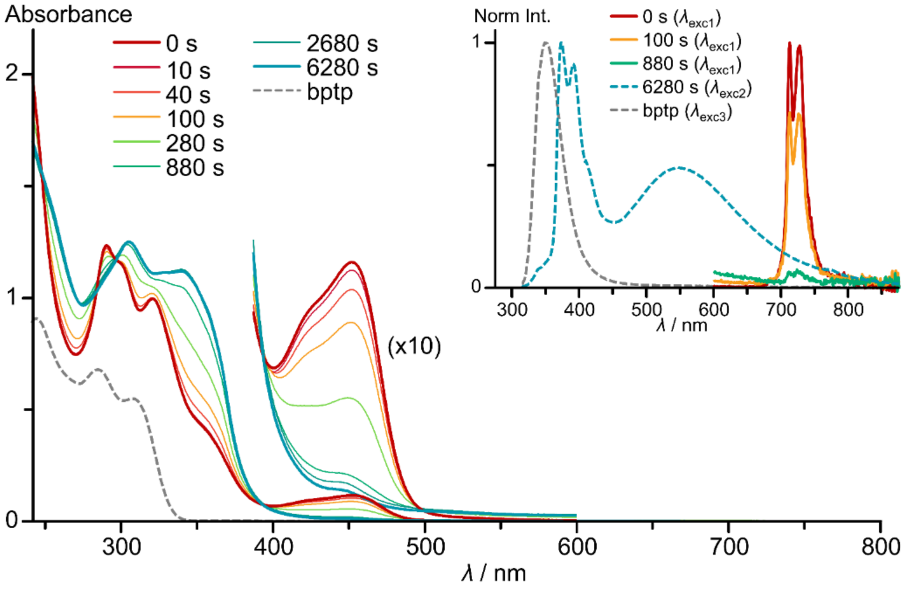

Fig. S72 UV/VIS/NIR absorption (with zoom) and normalised emission spectra of 0.061 mM **[1^{S}][OTf]$_3$** in deaerated acetonitrile after different UHP-LED irradiation time periods (λ_{exc} = 460 nm) with an output power of 1.1 W and UV/VIS/NIR absorption and normalised emission spectra of bptp in acetonitrile (λ_{exc1} = 452 nm, λ_{exc2} = 308 nm, λ_{exc3} = 308 nm).

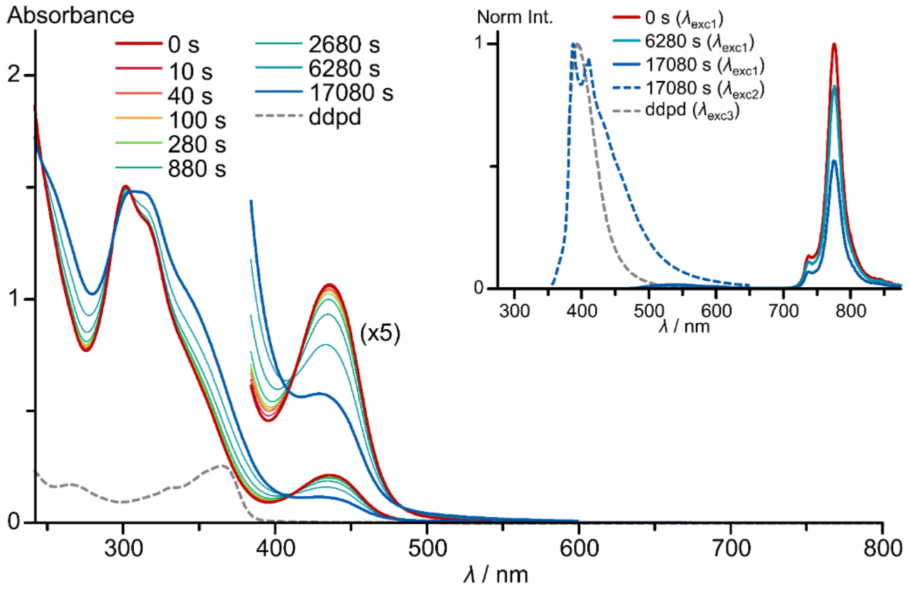

Fig. S73 UV/VIS/NIR absorption (with zoom) and normalised emission spectra of 0.066 mM **[1^{NMe}][OTf]$_3$** in deaerated acetonitrile after different UHP-LED irradiation time periods (λ_{exc} = 460 nm) with an output power of 1.1 W and UV/VIS/NIR absorption and normalised emission spectra of ddpd in dichloromethane (λ_{exc1} = 436 nm, λ_{exc2} = 345 nm, λ_{exc3} = 369 nm).[44]

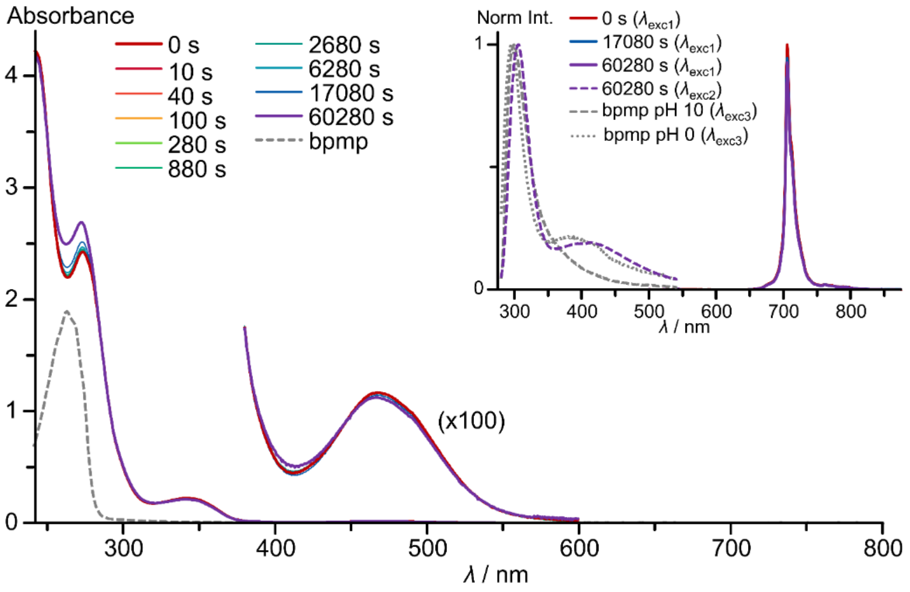

Fig. S74 UV/VIS/NIR absorption (with zoom) and normalised emission spectra of 0.177 mM **[1^{CH2}][OTf]$_3$** in deaerated acetonitrile after different UHP-LED irradiation time periods (λ_{exc} = 460 nm) with an output power of 1.1 W and UV/VIS/NIR absorption and normalised emission spectra of bpmp in water at pH = 0 and pH = 10 (λ_{exc1} = 467 nm, λ_{exc2} = 270 nm, λ_{exc3} = 266 nm).[43]

Quenching Experiments

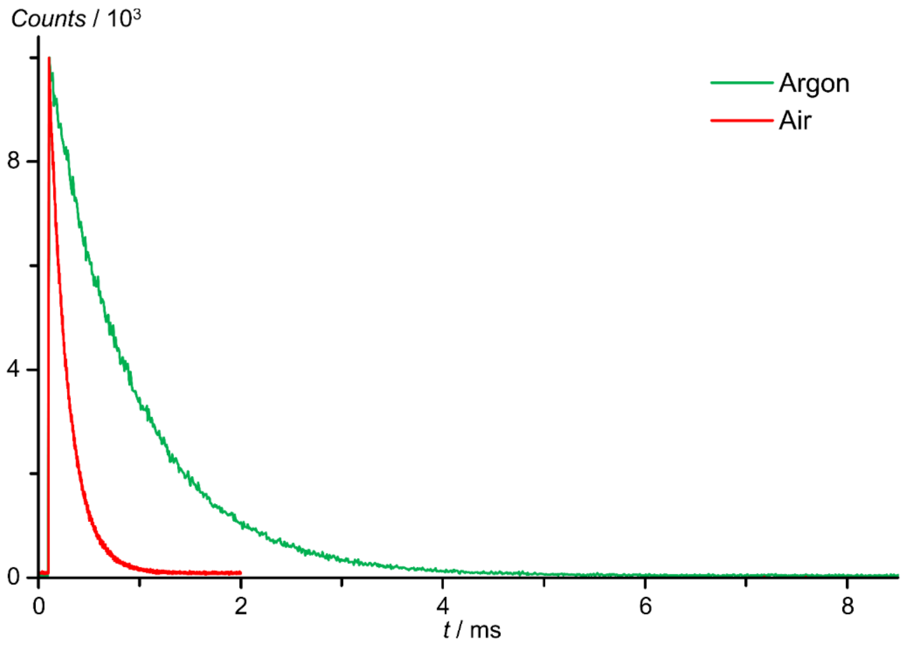

Fig. S75 Emission decay traces recorded at 742 nm following excitation (λ_{exc} = 450 nm) of a solution of **[1^{O}][OTf]$_3$** in deaerated acetonitrile (green) and air saturated acetonitrile (red).

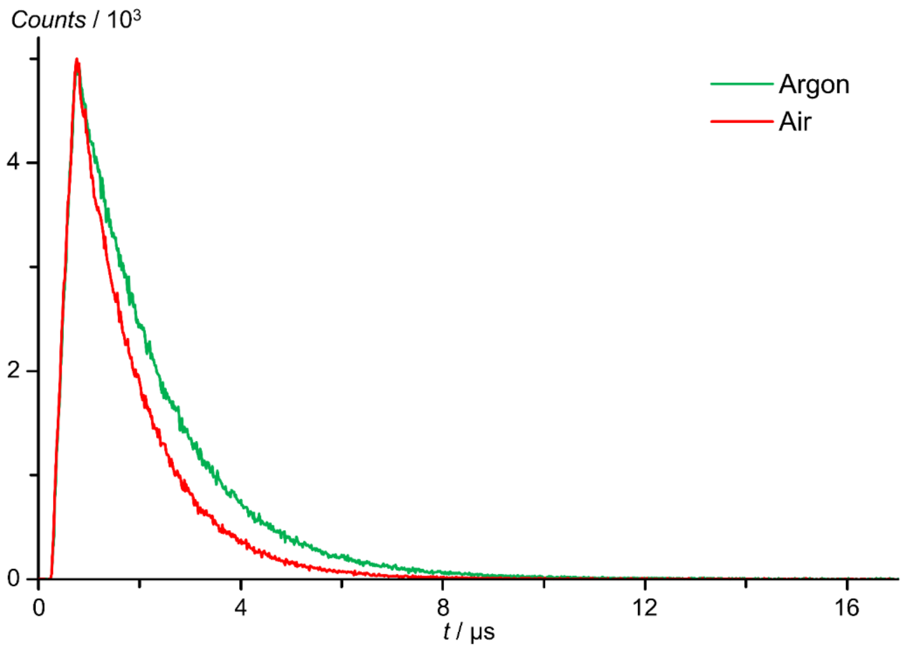

Fig. S76 Emission decay traces recorded at 729 nm following excitation (λ_{exc} = 450 nm) of a solution of **[1^{S}][OTf]$_3$** in deaerated acetonitrile (green) and air saturated acetonitrile (red).

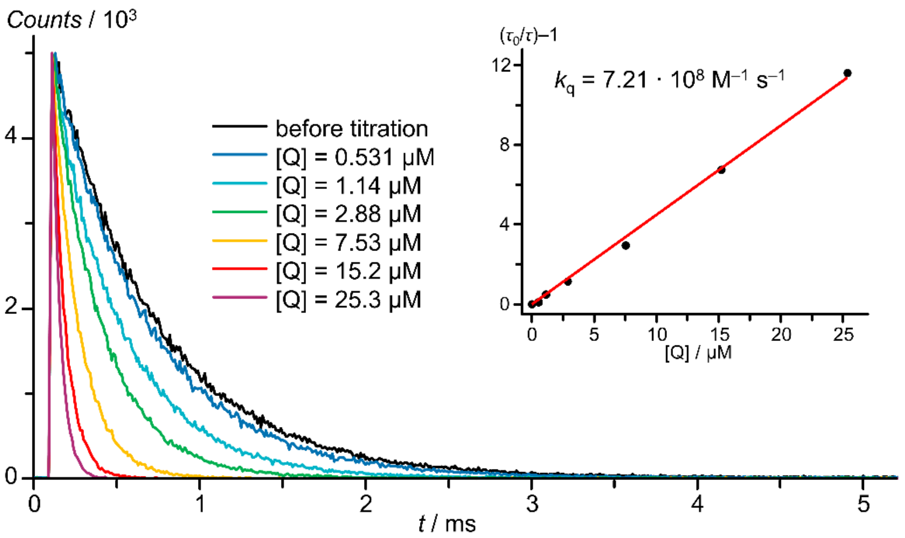

Fig. S77 Quencher concentration dependent emission decay traces recorded at 742 nm following excitation (λ_{exc} = 450 nm) of a solution of **[1^{O}][OTf]$_3$** in deaerated acetonitrile for Stern-Volmer analysis with anthracene and the corresponding Stern-Volmer plot with linear fit (red line).

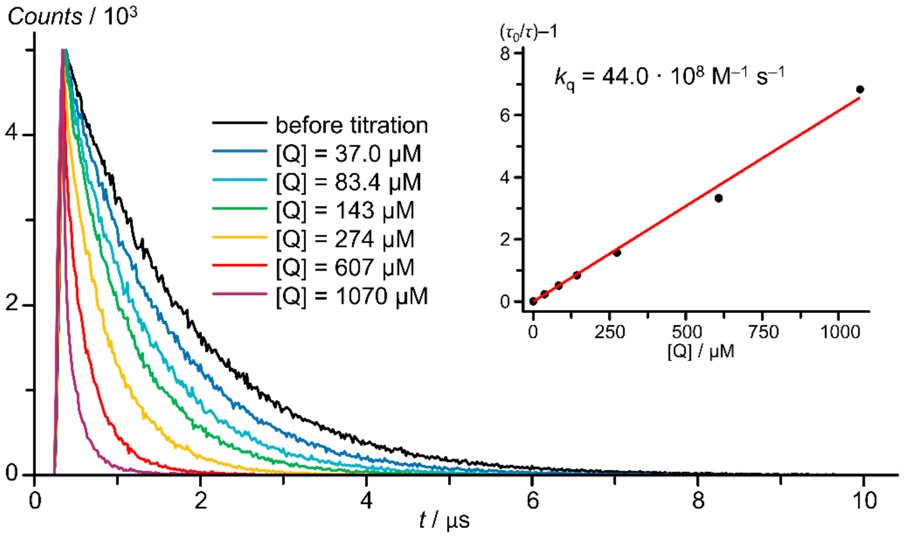

Fig. S78 Quencher concentration dependent emission decay traces recorded at 729 nm following excitation (λ_{exc} = 450 nm) of a solution of **[1^{S}][OTf]$_3$** in deaerated acetonitrile for Stern-Volmer analysis with anthracene and the corresponding Stern-Volmer plot with linear fit (red line).

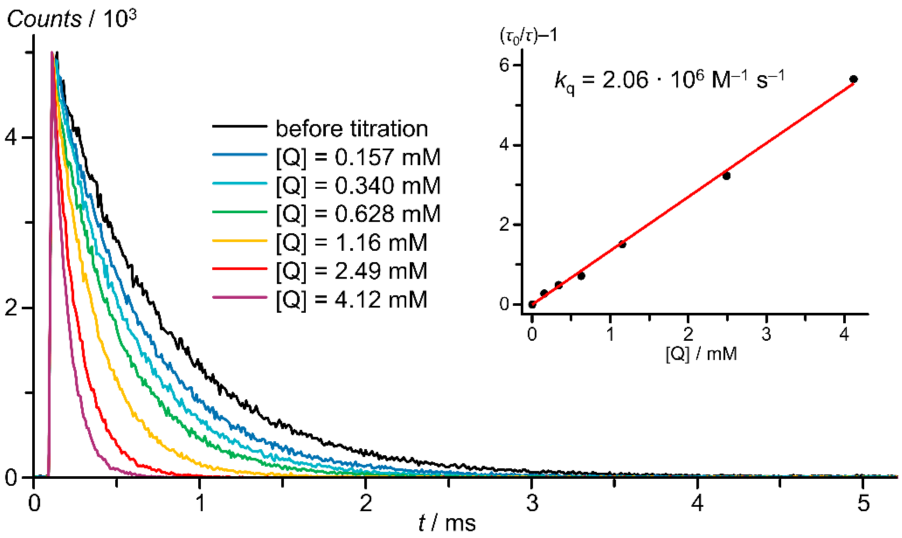

Fig. S79 Quencher concentration dependent emission decay traces recorded at 742 nm following excitation (λ_{exc} = 450 nm) of a solution of **[1^{O}][OTf]$_3$** in deaerated acetonitrile for Stern-Volmer analysis with trans-stilbene and the corresponding Stern-Volmer plot with linear fit (red line).

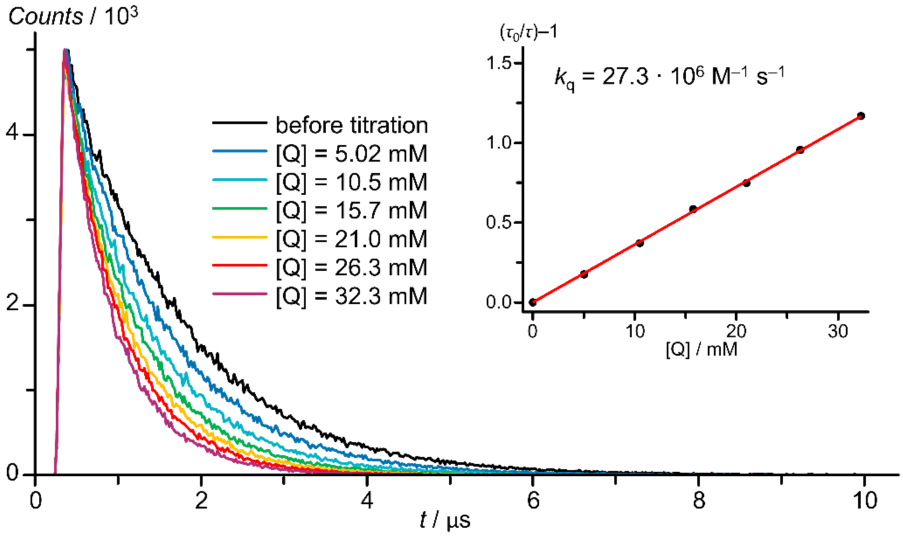

Fig. S80 Quencher concentration dependent emission decay traces recorded at 729 nm following excitation (λ_{exc} = 450 nm) of a solution of **[1^{S}][OTf]$_3$** in deaerated acetonitrile for Stern-Volmer analysis with trans-stilbene and the corresponding Stern-Volmer plot with linear fit (red line).

References

[1] H. E. Gottlieb, V. Kotlyar and A. Nudelman, *J. Org. Chem.*, 1997, **62**, 7512–7515.

[2] F. Barigelletti, D. Sandrini, M. Maestri, V. Balzani, A. von Zelewsky, L. Chassot, P. Jolliet and U. Maeder, *Inorg. Chem.*, 1988, **27**, 3644–3647.

[3] C. Müller, T. Pascher, A. Eriksson, P. Chabera and J. Uhlig, *J. Phys. Chem. A.*, 2022, **126**, 4087–4099.

[4] J. R. Lakowicz, *Principles of Fluorescence Spectroscopy,* Springer, New York, 2010.

[5] F. Neese, *WIREs Comp. Mol. Sci.*, 2012, **2**, 73–78.

[6] F. Neese, *WIREs Comp. Mol. Sci.*, 2022, **12**, 12753.

[7] C. Lee, W. Yang and R. G. Parr, *Phys. Rev. B: Condens. Matter Mater. Phys.*, 1988, **37**, 785–789.

[8] B. Miehlich, A. Savin, H. Stoll and H. Preuss, *Chem. Phys. Lett.*, 1989, **157**, 200–206.

[9] A. D. Becke, *J. Chem. Phys.*, 1993, **98**, 5648–5652.

[10] F. Weigend and R. Ahlrichs, *Phys. Chem. Chem. Phys.*, 2005, **7**, 3297–3305.

[11] F. Neese, F. Wennmohs, A. Hansen and U. Becker, *Chem. Phys.*, 2009, **356**, 98–109.

[12] R. Izsák and F. Neese, *J. Chem. Phys.*, 2011, **135**, 144105.

[13] F. Weigend, *Phys. Chem. Chem. Phys.*, 2006, **8**, 1057–1065.

[14] D. A. Pantazis and F. Neese, *J. Chem. Theory Comput.*, 2009, **5**, 2229–2238.

[15] D. A. Pantazis, X.-Y. Chen, C. R. Landis and F. Neese, *J. Chem. Theory Comput.*, 2008, **4**, 908–919.

[16] D. A. Pantazis and F. Neese, *Theor. Chem. Acc.*, 2012, **131**, 89.

[17] D. A. Pantazis and F. Neese, *J. Chem. Theory Comput.*, 2011, **7**, 677–684.

[18] C. van Wüllen, *J. Chem. Phys.*, 1998, **109**, 392–399.

[19] E. van Lenthe, E. J. Baerends and J. G. Snijders, *J. Chem. Phys.*, 1993, **99**, 4597–4610.

[20] S. Miertuš, E. Scrocco and J. Tomasi, *Chem. Phys.*, 1981, **55**, 117–129.

[21] V. Barone and M. Cossi, *J. Phys. Chem. A*, 1998, **102**, 1995–2001.

[22] S. Grimme, J. Antony, S. Ehrlich and H. Krieg, *J. Chem. Phys.*, 2010, **132**, 154104.

[23] S. Grimme, S. Ehrlich and L. Goerigk, *J. Comput. Chem.*, 2011, **32**, 1456–1465.

[24] F. Plasser, *J. Chem. Phys.*, 2020, **152**, 84108.

[25] B. O. Roos, P. R. Taylor and P. E. M. Sigbahn, *Chem. Phys.*, 1980, **48**, 157–173.

[26] P. E. M. Siegbahn, J. Almlöf, A. Heiberg and B. O. Roos, *J. Chem. Phys.*, 1981, **74**, 2384–2396.

[27] C. Angeli, R. Cimiraglia, S. Evangelisti, T. Leininger and J.-P. Malrieu, *J. Chem. Phys.*, 2001, **114**, 10252–10264.

[28] C. Angeli, R. Cimiraglia and J.-P. Malrieu, *Chem. Phys. Lett.*, 2001, **350**, 297–305.

[29] C. Angeli, R. Cimiraglia and J.-P. Malrieu, *J. Chem. Phys.*, 2002, **117**, 9138–9153.

[30] K. Pierloot, *Int. J. Quantum Chem.*, 2011, **111**, 3291–3301.

[31] STOE & Cie, X-Area, STOE & Cie GmbH, Darmstadt, Germany.

[32] R. H. Blessing, *Acta Crystallogr. A* 1995, **51**, 33–38.

[33] A. L. Spek, *Acta Crystallogr. D* 2009, **65**, 148–155.

[34] J. Koziskova, F. Hahn, J. Richter and J. Kožíšek, *Acta Chim. Slovaca* 2016, **9**, 136–140.

[35] STOE & Cie, X-Area LANA, STOE & Cie GmbH, Darmstadt, Germany.

[36] G. M. Sheldrick, *Acta Crystallogr., Sect. A: Found. Adv.*, 2015, **71**, 3–8.

[37] G. M. Sheldrick, *Acta Crystallogr., Sect. C: Struct. Chem.*, 2015, **71**, 3–8.

[38] G. M. Sheldrick, *Acta Crystallogr., Sect. A: Found. Crystallogr.*, 2008, **64**, 112–122.

[39] C. B. Hübschle, G. M. Sheldrick and B. Dittrich, *J. Appl. Crystallogr.*, 2011, **44**, 1281–1284.

[40] P.-S. Wang, C.-K. Liang and M.-k. Leung, *Tetrahedron*, 2005, **61**, 2931–2939.

[41] S. Balamurugan, S. Ganesan, S. Kamaraj, V. Mathew, J. Kim, N. Arumugam and A. I. Almansour, *Optical Materials*, 2022, **125**, 112082.

[42] R. D. Köhn, A. G. N. Coxon, S. Chunawat, C. Heron, S. Mihan, C. L. Lyall, S. B. Reeksting and G. Kociok-Köhn, *Polyhedron*, 2020, **185**, 114572.

[43] F. Reichenauer, C. Wang, C. Förster, P. Boden, N. Ugur, R. Báez-Cruz, J. Kalmbach, L. M. Carrella, E. Rentschler, C. Ramanan, G. Niedner-Schatteburg, M. Gerhards, M. Seitz, U. Resch-Genger and K. Heinze, *J. Am. Chem. Soc.*, 2021, **143**, 11843–11855.

[44] S. Otto, M. Grabolle, C. Förster, C. Kreitner, U. Resch-Genger, K. Heinze, *Angew. Chem. Int. Ed. Engl.*, 2015, **54**, 11572–11576.
